# Diagnostic Performance of ChatGPT-4o in Analyzing Oral Mucosal Lesions: A Comparative Study with Experts

**DOI:** 10.3390/medicina61081379

**Published:** 2025-07-30

**Authors:** Luigi Angelo Vaira, Jerome R. Lechien, Antonino Maniaci, Andrea De Vito, Miguel Mayo-Yáñez, Stefania Troise, Giuseppe Consorti, Carlos M. Chiesa-Estomba, Giovanni Cammaroto, Thomas Radulesco, Arianna di Stadio, Alessandro Tel, Andrea Frosolini, Guido Gabriele, Giannicola Iannella, Alberto Maria Saibene, Paolo Boscolo-Rizzo, Giovanni Maria Soro, Giovanni Salzano, Giacomo De Riu

**Affiliations:** 1 Maxillofacial Surgery Operative Unit, Department of Medicine, Surgery and Pharmacy, University of Sassari, 07100 Sassari, Italy; gderiu@uniss.it; 2 Department of Surgery, Mons School of Medicine, UMONS Research Institute for Health Sciences and Technology, University of Mons (UMons), 7000 Mons, Belgium; jerome.lechien@umons.ac.be; 3 Department of Otolaryngology-Head Neck Surgery, Elsan Polyclinic of Poitiers, 86000 Poitiers, France; 4 Department of Medicine and Surgery, University of Enna Kore, 94100 Enna, Italy; tnmaniaci29@gmail.com; 5 Infectious Diseases Operative Unit, Department of Medicine, Surgery and Pharmacy, University of Sassari, 07100 Sassari, Italy; andreadevitoaho@gmail.com; 6 Otorhinolaryngology, Head and Neck Surgery Department, Complexo Hospitalario Universitario A Coruña (CHUAC), 15006 A Coruña, Spain; 7 Head and Neck Section, Department of Neurosciences, Reproductive and Odontostomatological Science, Federico II University of Naples, 80138 Naples, Italy; stefy.troise@gmail.com (S.T.); giovannisalzanomd@gmail.com (G.S.); 8 Division of Maxillofacial Surgery, Department of Neurological Sciences, Marche University Hospitals—Umberto I, 60126 Ancona, Italy; giuseppe.consorti@ospedaliriuniti.marche.it; 9 Department of Biomedical Sciences and Public Health, Polytechnic University of Marche, 60126 Ancona, Italy; 10 Department of Otorhinolaryngology-Head & Neck Surgery, Hospital Universitario Donostia, 20014 San Sebastian, Spain; chiesaestomba86@gmail.com; 11 ENT Department, Morgagni Pierantoni Hospital, AUSL Romagna, 47121 Forlì, Italy; giovanni.cammaroto@hotmail.com; 12 ENT-HNS Department, Aix Marseille University, APHM, CNRS, IUSTI, La Conception University Hospital, 13001 Marseille, France; thomas.radulesco@ap-hm.fr; 13 Otolaryngology Unit, GF Ingrassia Department, University of Catania, 95128 Catania, Italy; ariannadistadio@hotmail.com; 14 Clinic of Maxillofacial Surgery, Department of Head & Neck Surgery and Neuroscience, University Hospital of Udine, 33100 Udine, Italy; alessandro.tel@icloud.com; 15 Department of Maxillofacial Surgery, University of Siena, 53100 Siena, Italy; andreafrosolini@gmail.com (A.F.); guido.gabriele@unisi.it (G.G.); 16 Department of ‘Organi di Senso’, University “Sapienza”, 00185 Rome, Italy; giannicola.iannella@uniroma1.it; 17 Otolaryngology Unit, Santi Paolo e Carlo Hospital, Department of Health Sciences, University of Milan, 20133 Milan, Italy; alberto.saibene@gmail.com; 18 Department of Medical, Surgical and Health Sciences, Section of Otolaryngology, University of Trieste, 34149 Trieste, Italy; pboscolorizzo@yahoo.it; 19 University of Sassari, 07100 Sassari, Italy; gmsoro@uniss.it

**Keywords:** ChatGPT, artificial intelligence, AI, maxillofacial surgery, otorhinolaryngology, medical image analysis, large language models, oral mucosal lesions, AI-assisted diagnosis, clinical decision support

## Abstract

*Background and Objectives:* this pilot study aimed to evaluate the diagnostic accuracy of ChatGPT-4o in analyzing oral mucosal lesions from clinical images. *Materials and Methods*: a total of 110 clinical images, including 100 pathological lesions and 10 healthy mucosal images, were retrieved from Google Images and analyzed by ChatGPT-4o using a standardized prompt. An expert panel of five clinicians established a reference diagnosis, categorizing lesions as benign or malignant. The AI-generated diagnoses were classified as correct or incorrect and further categorized as plausible or not plausible. The accuracy, sensitivity, specificity, and agreement with the expert panel were analyzed. The Artificial Intelligence Performance Instrument (AIPI) was used to assess the quality of AI-generated recommendations. *Results:* ChatGPT-4o correctly diagnosed 85% of cases. Among the 15 incorrect diagnoses, 10 were deemed plausible by the expert panel. The AI misclassified three malignant lesions as benign but did not categorize any benign lesions as malignant. Sensitivity and specificity were 91.7% and 100%, respectively. The AIPI score averaged 17.6 ± 1.73, indicating strong diagnostic reasoning. The McNemar test showed no significant differences between AI and expert diagnoses (*p* = 0.084). *Conclusions:* In this proof-of-concept pilot study, ChatGPT-4o demonstrated high diagnostic accuracy and strong descriptive capabilities in oral mucosal lesion analysis. A residual 8.3% false-negative rate for malignant lesions underscores the need for specialist oversight; however, the model shows promise as an AI-powered triage aid in settings with limited access to specialized care.

## 1. Introduction

Oral mucosal lesions encompass a broad spectrum of conditions ranging from benign reactive lesions to potentially malignant and malignant disorders [1]. Early and accurate diagnosis of these lesions is essential for effective management, as delayed detection of malignant transformations significantly impacts patient outcomes [2]. Traditionally, diagnosis relies on clinical examination by experienced specialists, often supplemented by histopathological analysis. However, access to specialized healthcare professionals remains a challenge, particularly in remote and underserved areas, necessitating the development of alternative diagnostic approaches [3]. Moreover, even in settings with access to specialized care, oral cavity lesions are frequently encountered by non-specialist healthcare providers due to the area’s easy accessibility during routine clinical examination performed by general practitioners and dental procedures [4,5].

Artificial intelligence (AI) has recently emerged as a promising adjuvant tool in medical diagnostics [6,7,8,9]. In oral medicine, AI-driven image recognition systems have been explored for lesion classification, showing potential in aiding early diagnosis [10,11]. However, most AI-based models developed to date rely on traditional supervised machine learning (ML) approaches, which require extensive datasets for training and significant computational resources [12]. These models demand substantial effort to curate and label data, limiting their scalability and real-world applicability.

In contrast, large language models (LLMs) represent a paradigm shift in AI utilization. Unlike supervised ML algorithms that require labor-intensive data training, LLMs are pre-trained on vast datasets and autonomously learn to recognize patterns, enabling them to function across a wide range of tasks, including medical image and video analysis. This advancement in AI could present a unique opportunity for application in complex diagnostic scenarios, potentially offering a readily available and widely accessible alternative to conventional machine-learning-based models.

Over the last twelve months, several next-generation vision-language models (VLMs) have been released. Proprietary systems such as Google Gemini 2.5 Pro and Flash, Anthropic Claude-3 Opus Vision, and OpenAI’s own GPT-4o-V have demonstrated competitive or superior performance on benchmarks such as the NEJM Image Challenge, radiology case series, and dermatology image sets [13,14,15]. Concurrently, open-source initiatives (e.g., R-LLaVA, KG-LLaVA, and CXR-LLaVA) are rapidly closing the gap, achieving state-of-the-art results on Med-VQA and chest-X-ray interpretation tasks [16,17]. This rapid progress underscores the strategic importance of evaluating LLM-based diagnostic tools in real-world clinical scenarios.

The performance of LLMs in answering theoretical questions and handling clinical scenarios in head and neck surgery has already been investigated by several authors, with promising results [18,19,20]. Studies suggest that these models can provide relevant insights and assist in decision-making processes [21,22,23,24,25,26] within specialized medical domains, although their diagnostic accuracy in image-based tasks remains an open question.

AI systems that generate diagnostic outputs for patient care are now explicitly regulated. Under Article 6 of the 2024 EU AI Act, such systems are classified as high-risk, triggering mandatory requirements for risk management, transparency, human oversight and post-market monitoring. In the United States, the FDA’s December 2024 final guidance on Predetermined Change Control Plans for AI/ML-enabled Software as a Medical Device (SaMD) outlines a lifecycle approach that allows performance-improving model updates while preserving safety and effectiveness. International standards such as ISO/IEC TR 24028:2020 [27] provide complementary technical guidance on trustworthiness (robustness, privacy, accountability). These frameworks informed the safety guardrails used in the present study and are discussed in depth below.

Despite these advancements, the clinical reliability of LLMs for medical image recognition remains unexplored, likely due to the fact that image analysis capabilities have only been introduced in the most recent versions. In this pilot study, we aim to assess the diagnostic accuracy of ChatGPT-4o in recognizing oral mucosal lesions from clinical images, comparing its performance with a panel of expert clinicians. Importantly, we evaluate ChatGPT in its untrained, publicly available version, without fine-tuning for specific medical tasks. By analyzing the concordance between AI-generated predictions and expert diagnoses, we seek to determine the feasibility and potential clinical utility of LLM-assisted diagnostic tools in oral pathology.

## 2. Materials and Methods

This study is part of an international collaboration involving members of the Italian Society of Maxillofacial Surgery and the Young Otolaryngologists of the International Federation of Otorhinolaryngological Societies (YO-IFOS), established in January 2023 to investigate the applications of conversational artificial intelligence in head and neck surgery. The study involved 17 researchers from 4 countries. Since no human subjects were included, ethical committee approval was not required, and the study was conducted in accordance with the principles of the Declaration of Helsinki. National privacy legislation (Regulation EU 2016/679 and Legislative Decree 101/2018) classifies intra-oral photographs as biometric data. Our institutional ethics committee advised that uploading such images to an external cloud-based AI service would require explicit patient consent for cross-border data transfer and a data-protection impact assessment. Because these prerequisites could not be fulfilled within the timeline of this exploratory project, we opted to use images already in the public domain, thereby avoiding additional privacy risk. Consequently, the present work should be regarded as a proof-of-concept pilot study guiding a forthcoming, ethics-approved clinical validation.

### 2.1. Image Selection and Dataset Construction

Clinical images were retrieved from Google Images (Google LLC, Mountain View, CA, USA) through an online search conducted on 28 January 2025. Two researchers screened and selected 100 images of oral mucosal lesions and 10 images of healthy oral mucosa. The search was performed using the query “oral mucosal lesions”, while ensuring that only high-resolution images were considered by applying the corresponding search filter. Images published before June 2024 have been excluded.

Images had to meet all of the following inclusion criteria: (i) in-focus color photograph; (ii) lesion (or normal mucosa) fully visible with ≤25% background occlusion; (iii) resolution ≥ 800 × 600 px; (iv) no arrows, labels, stains or grids. Exclusion criteria were low-resolution or motion-blurred images; extreme lighting/contrast filters; intra-operative photographs; images published before June 2024; and duplicates detected by Google Lens. Screening proceeded in three rounds (title-thumbnail, full-size inspection, duplicate check) carried out independently by the two investigators, with disagreements resolved by discussion.

To ensure objectivity, each selected image was renamed with a sequential numerical identifier, preventing any indication of its original source. The same methodology was applied to the selection of images of healthy oral mucosa. To limit potential data leakage, each candidate image underwent a Google Lens reverse-image search; any photograph with a visually identical replica indexed before June 2024 was excluded.

### 2.2. AI-Based Image Assessment

The 100 pathological images and 10 images of healthy mucosa were uploaded into ChatGPT-4o on 13 and 14 February 2025. Each image was analyzed in a separate chat session to prevent contextual learning effects. The AI model used was a publicly available, untrained version of ChatGPT-4o (OpenAI, San Francisco, CA, USA), with no fine-tuning for medical tasks. The ChatGPT-4o instance was used with the built-in browsing functionality disabled, ensuring that the model had no real-time internet access and therefore could not retrieve or match the study images online.

A standardized SMART prompt [28] was used to ensure consistency in AI responses [Table 1].

The prompt simulated a specialist consultation, instructing the AI to act as a leading expert in oral pathology. Specifically, the AI was asked to analyze the image, determine whether a mucosal lesion was present, provide the most probable diagnosis, explicitly state when no lesion was detected, and propose an appropriate diagnostic and therapeutic plan. All AI-generated responses were collected and stored for further analysis [Table 2].

### 2.3. Expert Panel Evaluation

To establish a reference standard, the 110 images (100 pathological and 10 healthy) were collegially evaluated by a panel of five expert clinicians, consisting of two maxillofacial surgeons and three otorhinolaryngologists, all with more than 20 years of experience in oral pathology and head and neck oncology. The panel assessed the images and reached a consensus on the most probable diagnosis, which was recorded as the reference standard. Panelists deliberated together and were instructed to reach unanimous consensus for each image; individual interim ratings were therefore not retained. The 100 pathological images were further classified by the expert panel as benign or malignant based on histopathological or well-established clinical criteria. This classification was then used as the gold standard for evaluating ChatGPT-4o’s ability to correctly identify malignant lesions as malignant and benign lesions as benign.

The AI-generated diagnoses were compared to the expert consensus diagnoses and classified as correct when they matched the expert assessment or incorrect when they differed. For incorrect diagnoses, the expert panel further categorized the AI response as plausible, when the suggested diagnosis was a reasonable differential, or not plausible, when the diagnosis was deemed clinically implausible. Because the consensus approach yields a single “ground-truth” label per case, inter-rater reliability statistics such as Cohen’s or Fleiss’ κ were not applicable to this dataset.

Additionally, the AI-generated diagnostic and therapeutic recommendations were evaluated using the Artificial Intelligence Performance Instrument (AIPI) [29], a validated tool designed to assess the performance of AI models in clinical decision-making. The AIPI score evaluates different aspects of clinical reasoning, including the accuracy of patient history interpretation, symptom assessment, differential diagnosis, selection of additional examinations, and treatment planning. The total score ranges from 0 to 20.

### 2.4. Statistical Analysis

Statistical analyses were performed using Jamovi 2.6.25 (The Jamovi Project, Sydney, Australia). The accuracy of the AI model was calculated as the percentage of correct diagnoses relative to the total number of cases. Continuous variables are reported as the mean ± standard deviation. Marginal homogeneity between AI and expert binary classifications (malignant vs. benign) was assessed with the exact McNemar test because one discordant cell contained zero cases. The sensitivity, specificity, positive predictive value (PPV), and negative predictive value (NPV) of ChatGPT-4o were calculated to assess its ability to differentiate between benign and malignant lesions. A *p*-value < 0.05 was considered statistically significant for all comparisons.

## 3. Results

ChatGPT-4o correctly identified the normal mucosa in 3 out of 10 cases [Supplementary File S1]. In the remaining seven cases, it mistakenly identified the presence of a lesion. Specifically, it diagnosed frictional keratosis in two cases, lingual varices in two cases, amalgam tattoo in one case, nicotine stomatitis in one case, and inflammatory papillary hyperplasia in one case [Supplementary File S2].

In the pathological lesion group [Supplementary File S3], ChatGPT-4o provided a correct diagnosis in 85% of cases (85 out of 100). Among the 15 incorrect diagnoses, the expert panel judged 10 as plausible and 5 as not plausible [Supplementary File S4]. In all cases, the correct diagnosis, although not identified as the most probable, was included in the AI-generated differential diagnoses.

The errors involved the misclassification of a palatal salivary gland adenoma as a torus palatinus, an oral leukoplakia as lichen planus, and a hereditary hemorrhagic telangiectasia as a herpetic infection. Additionally, two cases of oral melanoma were misdiagnosed as squamous cell carcinoma, while erythroplakia was mistaken for erosive lichen planus. Mandibular torus was misclassified as oral submucous fibrosis in one case and as pyogenic granuloma in another. Similarly, an alveolar exostosis was identified as gingival hyperplasia, a traumatic ulcer as lichen planus, and squamous cell carcinoma was mistaken for medication-related osteonecrosis of the jaws in two instances. Another squamous cell carcinoma was classified as erythroleukoplakia, an aphthous ulcer was misinterpreted as a mucocele, and erythroplakia was confused with contact mucositis.

ChatGPT-4o classified three malignant lesions as benign, while no benign lesions were misclassified as malignant. An exact McNemar test comparing paired benign/malignant decisions (0 AI-positive/expert-negative vs. 3 AI-negative/expert-positive) yielded *p* = 0.083 (two-sided). This value is close to the conventional 0.05 threshold, but with only three discordant pairs the test has very low power (~22% for a 75% vs. 25% imbalance) and should be interpreted with caution.

ChatGPT-4o demonstrated a sensitivity of 91.7% and a specificity of 100%, with an overall accuracy of 97.0% in distinguishing benign from malignant lesions. The 95% confidence intervals were 77.5–98.2% for sensitivity and 95.1–100% for specificity. The PPV was 100%, while the negative NPV was 95.5%. The post-test probability of disease when ChatGPT-4o predicted malignancy was 100%, whereas the post-test probability of health when the AI classified a lesion as benign was 95.5%. The positive likelihood ratio was infinite, while the negative likelihood ratio was 0.0833, further indicating the strong discriminatory power of ChatGPT-4o in this task.

Finally, the mean AIPI score was 17.6 ± 1.73, reflecting a consistently high level of diagnostic and therapeutic appropriateness in the AI-generated responses.

## 4. Discussion

This study provides preliminary evidence that ChatGPT-4o has the potential to assist in the diagnosis of oral mucosal lesions through image analysis. The AI demonstrated high accuracy in differentiating between benign and malignant lesions, with a sensitivity of 91.7% and a specificity of 100%. The McNemar test indicated no significant difference between ChatGPT-4o and expert clinicians, supporting its reliability as a diagnostic aid. Despite these promising results, the system still misclassified three malignant lesions as benign, highlighting the need for further refinement before clinical implementation.

Previous studies on LLMs in medicine have primarily focused on their ability to answer theoretical questions and assist in clinical decision-making based on textual scenarios [12,17,18,19,29,30,31,32,33]. While these investigations have demonstrated promising results, the accuracy of LLMs in direct medical image recognition remains largely unexplored. Unlike traditional AI models trained specifically for image classification using supervised learning, LLMs such as ChatGPT-4o have only recently integrated multimodal capabilities, allowing them to interpret images in addition to processing text-based inputs. This technological advancement has enabled LLMs to extend their applications beyond text-based clinical reasoning to image-based diagnostic tasks, potentially representing a significant paradigm shift in medical AI research. LLMs like ChatGPT, are based in transformers architecture, and nowadays this is the most used architecture in the developing of AI-based image recognition, due to the advantage of image tokenization [34].

To date, only a limited number of studies have evaluated the ability of LLMs with vision capabilities in real-world medical imaging. Busch et al. [35] assessed the diagnostic performance of GPT-4o, showing that it outperformed text-only models in analyzing complex radiological cases. However, its accuracy remained inferior to that of domain-specific AI models, highlighting the challenges of applying LLMs to medical image diagnostics. Another study [16] explored ChatGPT-4 in osteosarcoma detection from X-ray images, demonstrating that while the model could differentiate occupying from non-occupying bone lesions with reasonable accuracy, it exhibited low sensitivity for distinguishing malignant from benign lesions, leading the authors to conclude that LLMs are not yet suitable for independent radiological diagnosis. Additionally, research on laryngoscopic images [36] found that while Google Gemini correctly identified the procedure and gross pathological features, its diagnostic accuracy remained limited, particularly in differentiating similar pathologies. Finally, Chiesa-Estomba et al. [37] found that ChatGPT-4o demonstrated high sensitivity in identifying laryngeal malignancies but exhibited limited specificity and moderate consistency when analyzing clinical fiberoptic videos, indicating a notable reliance on textual rather than visual data interpretation.

These findings align with our study, which confirms that ChatGPT-4o delivers robust descriptive output yet cannot, at present, be deployed as an autonomous diagnostic device. Readiness will require demonstrable progress on four fronts: (i) technical robustness —prospective, multi-center external validation that meets TRIPOD-AI reporting standards and proves ≥ non-expert-clinician accuracy plus adversarial stress-testing [38]; (ii) clinical efficacy and safety—at least one DECIDE-AI stage-2 or CONSORT-AI randomized trial showing non-inferiority to board-certified oral-medicine specialists [39,40]; (iii) regulatory conformity—a Good Machine-Learning Practice dossier including a Predetermined Change Control Plan and, in Europe, successful class IIa/IIb conformity assessment under the MDR and the 2024 EU AI Act high-risk obligations; (iv) workflow and liability integration—SPIRIT-AI-guided usability studies, auditable human-in-the-loop pathways and clear allocation of professional responsibility among developers, clinicians and healthcare organizations.

One of the most intriguing implications of AI-driven diagnostic systems is their potential for large-scale accessibility, particularly in regions with limited access to specialized healthcare. A tool capable of screening oral mucosal lesions using patient-taken photographs could significantly enhance early detection efforts, guiding individuals toward appropriate medical evaluation. In this study, non-standardized images were deliberately used, varying in terms of lighting, angle, and camera position, to simulate real-world scenarios where patients themselves might capture and upload images for assessment. The ability of ChatGPT-4o to analyze such heterogeneous inputs and still achieve high diagnostic accuracy is noteworthy and suggests that LLM-based AI could play a role in patient-driven screening programs.

From a regulatory standpoint, vision–language diagnostic models such as ChatGPT-4o are now classified as high-risk systems under the 2024 EU Artificial Intelligence Act [41] and, when deployed clinically in Europe, must also comply with the Medical Device Regulation (MDR). In the United States, the FDA’s December 2024 guidance for AI/ML-enabled Software as a Medical Device introduces the concept of a Predetermined Change Control Plan, allowing adaptive model updates provided that performance boundaries and re-validation triggers are predefined [42]. Both frameworks mandate transparency, continuous human oversight and rigorous post-market monitoring.

Ethically, four issues warrant particular attention: (i) bias mitigation—training data should include diverse mucosal and skin phototypes to avoid misdiagnosis in under-represented groups; (ii) explainability—clinicians need clinically intelligible saliency maps or textual rationales to scrutinize each output; (iii) privacy and GDPR compliance—intra-oral photographs constitute biometric data and thus require explicit informed consent and robust de-identification; (iv) allocation of liability—AI outputs must augment, not replace, specialist judgment, and responsibility should be clearly apportioned among developers, clinicians, and healthcare institutions.

Beyond classification, ChatGPT-4o demonstrated exceptional lesion description capabilities. Even in cases where the final diagnosis was incorrect, the AI provided detailed and precise descriptions, recognizing key morphological features such as location, color, shape, ulceration, heterogeneity, and infiltrative characteristics suggestive of malignancy. This descriptive ability is particularly valuable, as it indicates that AI systems may assist clinicians not only in classification but also in lesion characterization, potentially aiding in clinical decision-making.

A particularly reassuring finding was that the correct diagnosis was always included in the differential diagnosis, even when not listed as the most probable one. Additionally, all differential diagnoses proposed by ChatGPT-4o were clinically plausible, demonstrating that the model is capable of generating meaningful diagnostic hypotheses that align with real-world clinical reasoning. Furthermore, the diagnostic and therapeutic pathway proposed by the AI was consistently precise, complete, and aligned with standard medical guidelines, as reflected by the mean AIPI score of 17.6 ± 1.73.

However, three malignant lesions were misclassified as benign, indicating that while specificity was optimal, some malignant conditions were underestimated by the AI. This finding aligns with previous concerns regarding AI-based models potentially prioritizing common benign conditions over critical malignant lesions [37]. An 8.3% false-negative rate would correspond to nearly one missed malignancy in every twelve cases—a clinically critical issue in oral oncology. Even a diagnostic delay of 3 months can reduce 5-year survival in oral squamous cell carcinoma (OSCC) by 7–10%, while also increasing the likelihood of advanced-stage presentation, need for multimodal treatment, and significant morbidity [43,44]. Unlike false positives, which mainly cause anxiety and additional diagnostic steps, false negatives may lead to catastrophic consequences such as treatment delay, disease progression, and reduced survival rates. For these reasons, any clinical deployment of ChatGPT-4o should prioritize sensitivity over specificity, integrate mandatory human-in-the-loop verification, and embed safety-net mechanisms (e.g., urgent biopsy referral for any persistent or suspicious lesion regardless of AI output, re-evaluation triggers after 2–4 weeks). In its current form, ChatGPT-4o could be deployed only within a human-in-the-loop triage workflow that includes (i) sensitivity-oriented thresholding and confidence flags, with every “benign/low-confidence” output automatically routed to specialist review; (ii) predefined escalation rules (e.g., urgent biopsy referral or teleconsultation when the AI mentions malignancy, ulceration with induration, or erythro/leukoplakic change); (iii) safety-netting protocols—patients with persistent symptoms are recalled for re-evaluation at 2–4 weeks irrespective of AI output; (iv) structured documentation and audit trails capturing the AI suggestion, clinician override/confirmation, and final decision; (v) data governance and consent procedures compliant with GDPR (explicit patient consent for image use, secure storage, de-identification); and (vi) continuous performance monitoring, with periodic re-calculation of sensitivity/specificity and targeted re-training or model replacement when drift is detected. Further, robust audit trails, decision-support confidence scoring, and continuous performance monitoring should be standard components to mitigate medico-legal risks associated with AI-assisted triage. Regarding healthy mucosa, ChatGPT-4o correctly classified normal tissue in only 4 out of 10 cases, suggesting a tendency toward over-diagnosis of benign conditions. This pattern suggests that ChatGPT-4o may have a higher sensitivity for detecting alterations but lacks the ability to confidently exclude pathology in cases of normal mucosa.

The integration of multimodal AI into oral pathology and head and neck oncology represents an exciting frontier for early diagnosis and patient triage. Future developments could focus on improving AI training with large-scale annotated datasets, enhancing its ability to recognize rare and atypical lesions. Moreover, the real-time application of AI-powered image analysis in telemedicine platforms could facilitate early lesion detection in primary care and underserved communities.

Further research should investigate the potential of ChatGPT-4o in assisting not only with initial diagnosis but also with monitoring lesion evolution over time, particularly for potentially malignant disorders. The ability to track subtle morphological changes using AI-based analysis could support personalized follow-up strategies and enhance early detection of malignant transformations.

Despite its strengths, this study has several limitations. First, it is important to note that the images used in this study were retrieved from publicly available online sources and were classified based on expert clinical evaluation, rather than histopathological confirmation. While the expert panel consisted of highly experienced specialists in oral pathology and head and neck oncology, the lack of histological verification for all images represents a potential limitation. Future studies should aim to validate these findings using datasets with confirmed histopathological diagnoses to enhance the reliability of the results. Because we used a unanimous consensus to define the reference diagnosis and did not retain individual ratings, we could not quantify inter-rater reliability or diagnostic variability among experts (e.g., κ, ICC). Future studies should capture individual assessments to report these metrics. Another potential limitation is the possibility that ChatGPT-4o may have been exposed to similar images during its pre-training phase. However, as OpenAI has not disclosed the exact datasets used for training, it is not possible to determine whether specific images or cases were included. The model was tested in its untrained, publicly available version, without fine-tuning for medical tasks, which suggests that its performance is based on general pattern recognition rather than memorized examples. Nevertheless, excluding images published before June 2024 and screening each candidate with Google Lens cannot fully eliminate data-leakage risk, because GPT-4o’s proprietary pre-training corpus is undisclosed. In other words, visually similar images could still have been seen by the model during training despite our filters. This residual possibility represents a key limitation of the present work. To remove this uncertainty, future validation will use prospectively collected, institution-owned images obtained after the model’s last training cut-off and kept entirely outside the public domain (and off third-party clouds) until evaluation is complete. Furthermore, the images used were obtained from online sources, which may not fully reflect the variability seen in clinical practice. Google Images could tend to showcase classic textbook examples; atypical early-stage lesions encountered in primary care may therefore be under-represented. Finally, the dataset encompassed 100 pathological and 10 normal cases; while sufficient for a proof-of-concept, this sample does not yield tight confidence intervals for rare entities such as melanoma or salivary-gland tumors (<5 cases each). Consistently, the 95% confidence interval for sensitivity (77.5–98.2%) is wide, reflecting the modest number of malignant cases (*n* = 36). Larger malignant cohorts are required to achieve ±5% precision. Likewise, the McNemar test was under-powered with only three discordant pairs, limiting inference about marginal asymmetry.

## 5. Conclusions

In conclusion, ChatGPT-4o exhibited high diagnostic accuracy, strong descriptive capabilities, and a reliable diagnostic workflow in analyzing oral mucosal lesions.

Nevertheless, it misclassified 3 of 36 malignant cases (false-negative rate = 8.3%), demonstrating that the model cannot yet be deployed as an autonomous diagnostic device. Under specialist supervision, however, it could serve as a first-line triage aid, particularly in areas with limited access to oral-medicine expertise.

To further validate these findings, future studies should replicate this analysis using prospectively collected clinical cases with confirmed histopathological diagnoses. This would provide a more robust assessment of the model’s diagnostic reliability in real-world settings and help determine whether AI-assisted diagnostic tools can be effectively integrated into clinical practice. In the interim, safe clinical use is feasible only as a supervised triage aid, following explicit protocols for escalation, safety-netting, documentation, and ongoing performance auditing.

## Figures and Tables

**Table 1:** SMART prompt used for ChatGPT-4o image-based diagnosis.

Seeker	I am a head and neck surgeon with a decade of experience in oral pathology.
Mission	A colleague has sent me an image of an oral mucosal lesion in a patient, asking for my help with the diagnosis.
AI role	You are the world’s leading expert in oral pathology.
Register	Correct and specialized scientific language. The information must be based on the most recent and solid scientific evidence.
Targeted question	Analyze the attached image, assess whether mucosal lesions are present, and, if so, provide the diagnosis that you consider the most probable. If no lesions are detected, state it explicitly. Additionally, provide the most appropriate diagnostic and therapeutic pathway for the patient.

**Table 2:** ChatGPT-4o diagnostic assessment and recommended management for an oral mucosal lesion—case example.

Image	ChatGPT4o Response
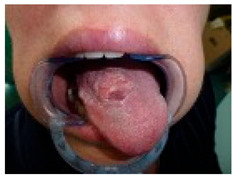	Analysis and Probable Diagnosis Observations: Exophytic, Ulcerated, and Indurated Lesion on the Dorsal Tongue: The lesion presents as an irregular, proliferative mass with ulceration and induration. The surface exhibits an irregular, verrucous texture with areas of erosion and leukoplakia. These features raise a strong suspicion of a malignant process, likely squamous cell carcinoma (SCC). Localization on the Mid-Dorsal Tongue: While SCC is most common on the lateral and ventral tongue, the dorsal tongue can also be affected. The lesion’s non-healing nature, ulceration, and infiltrative characteristics make malignancy highly probable. Leukoplakic and Erythroplakic Areas: Presence of mixed white and red patches suggests epithelial dysplasia or carcinoma in situ. Erythroleukoplakia carries a significantly higher malignant transformation potential than leukoplakia alone. Verrucous-Like Features: The lesion displays verrucous and exophytic growth, which could indicate verrucous carcinoma (if slow-growing, non-ulcerative, and well-differentiated) or squamous cell carcinoma (if ulcerated, infiltrative, and rapidly progressing). Most Probable Diagnosis: oral squamous cell carcinoma (OSCC). Key clinical features supporting SCC diagnosis include non-healing ulceration with induration, exophytic and infiltrative growth pattern, presence of erythroleukoplakia, irregular, roughened, and verrucous surface. Given these features, OSCC is the leading differential diagnosis and must be ruled out with a biopsy. Differential Diagnoses to Consider: Verrucous carcinoma typically presents as a slow-growing, exophytic lesion with less ulceration than SCC. Histopathology is necessary to differentiate between SCC and verrucous carcinoma. Chronic traumatic ulcer or hyperplasia consider if there is a history of persistent mechanical irritation (e.g., sharp teeth, dental prosthesis). Unlike SCC, reactive lesions are typically non-indurated and resolve once the source of trauma is removed. Chronic infectious ulcers (tuberculosis, syphilis, deep fungal infections). These conditions can cause persistent ulcerations, but typically lack the exophytic, verrucous component seen here. Recommended Next Steps: 1. Urgent Incisional Biopsy for Histopathological Diagnosis. A deep incisional biopsy with adequate sampling of the lesion’s central and peripheral areas is mandatory. Preferred biopsy site: the most representative area, avoiding necrotic or superficial regions. The histopathological report should assess: presence of keratin pearls, dysplastic changes, and invasive epithelial nests (indicative of SCC). Differentiation grade (well, moderately, or poorly differentiated). Depth of invasion (critical for staging and prognosis). Presence of lymphovascular or perineural invasion. 2. Imaging for Tumor Staging. If SCC is confirmed, imaging is required to evaluate local invasion and regional metastasis. Recommended imaging modalities: MRI of the tongue and floor of the mouth—to assess tumor extent and muscular infiltration. CT scan (head, neck, and chest)—to evaluate lymph node involvement and distant metastases. Ultrasound with fine-needle aspiration biopsy (FNAB)—if cervical lymphadenopathy is detected. 3. Comprehensive Oncologic Evaluation. Referral to a head and neck cancer multidisciplinary team (MDT). TNM Staging (AJCC 8th Edition) should be established to guide treatment planning. If SCC is confirmed, a treatment plan must be discussed based on tumor size (T staging), lymph node involvement (N staging), and the presence of distant metastasis (M staging). 4. Risk Factor Assessment and Patient Optimization. Evaluate for risk factors contributing to malignant transformation: tobacco use (smoking or smokeless tobacco), heavy alcohol consumption, HPV-associated carcinogenesis (p16 immunohistochemistry if suspected). The management of predisposing factors should be part of the long-term strategy. 5. Definitive Treatment (Based on Staging). If SCC is confirmed and localized (T1–T2, N0), surgery is the preferred treatment: wide local excision with adequate margins (≥1 cm); neck dissection (if nodal involvement is suspected). If SCC is advanced (T3–T4, N+), multimodal therapy is indicated: surgery + adjuvant radiation ± chemotherapy. Definitive chemoradiotherapy if unresectable. Conclusions: This lesion exhibits high-risk clinical features for oral squamous cell carcinoma (OSCC). Immediate incisional biopsy is mandatory for histopathological confirmation. If SCC is confirmed, comprehensive staging with imaging and multidisciplinary oncologic assessment must follow. Treatment planning should be based on TNM staging, patient comorbidities, and functional outcomes. Given the aggressive nature of OSCC, early diagnosis and timely intervention are critical for optimizing prognosis.

## Data Availability

Data are available in the Supplementary Materials.

## Supplementary Materials

The following supporting information can be downloaded at https://www.mdpi.com/article/10.3390/medicina61081379/s1; Supplementary File S1: ChatGPT-4o responses on healthy oral mucosa—complete dataset; Supplementary File S2: ChatGPT-4o responses on healthy oral mucosa—case-by-case analysis; Supplementary File S3: ChatGPT-4o responses on oral mucosal lesions—complete dataset; Supplementary File S4: ChatGPT-4o responses on oral mucosal lesions—case-by-case analysis.
