# Supplementary material for: Diagnostic Performance of ChatGPT-4o in Analyzing Oral Mucosal Lesions: A Comparative Study with Experts
Source: Medicina (Kaunas). 2025 Jul 30;61(8):1379. doi: 10.3390/medicina61081379 (PMC12388129; doi:10.3390/medicina61081379)
Supplement: Supplementary file 1 [file medicina-61-01379-s001.zip › Supplementary document S1.pdf]

Supplementary document S1: ChatGPT-4o responses on healthy oral mucosa – Complete dataset

CASE 1

Reference:

https://www.ildentistamoderno.com/files/2016/07/DM_2015_011_serpico023-460x307.jpg

Chat-GPT4 answer:

Analysis and Probable Diagnosis

Observations:

1. **Generalized opalescence and faint whitish areas on the buccal mucosa:**
   - **There is a diffuse, slightly translucent appearance of the mucosa, particularly in the posterior region.**
   - **The mucosa appears intact, without ulceration, nodularity, or exophytic growths.**
2. **No Clear Evidence of Pathological Lesions:**
   - **There is no leukoplakia, erythroplakia, or suspicious mass.**
   - **The mucosa exhibits a homogeneous, smooth texture.**
3. **Slight Reticular or Wrinkled Pattern in the Posterior Buccal Mucosa:**
   - **The presence of fine white striations or mild hyperkeratosis suggests a benign physiological or reactive process.**

Most Probable Diagnosis: Leukoedema

- **Leukoedema is a benign variation of normal oral mucosa, often seen as a diffuse, grayish-white opalescence.**
- **It is more noticeable in darker-skinned individuals and smokers.**
- **The key diagnostic feature: disappears or diminishes upon mucosal stretching (stretch test).**

Differential Diagnoses to Consider (If Symptomatic or Persistent):

1. **Frictional Keratosis (If Related to Chronic Trauma)**
   - **Often caused by cheek biting, dental restorations, or orthodontic appliances.**
   - **Typically presents as roughened, more focal keratotic plaques.**
2. **Oral Lichen Planus - Reticular Type (If Wickham's Striae Are Present)**
   - **Bilateral, lace-like white striae on the buccal mucosa.**
   - **May be asymptomatic or associated with burning sensations.**
3. **Early Leukoplakia (If Persistent and Non-Stretchable)**
   - **Leukoplakia is a premalignant white plaque that does not wipe off.**
   - **Biopsy is required if the lesion persists for >2 weeks or thickens.**
4. **Candidiasis (Unlikely Here)**
   - **Would present as white patches that can be wiped off, leaving an erythematous base.**
   - **More common in immunosuppressed individuals or those using inhaled corticosteroids.**

Recommended Next Steps:

1. **Stretch Test:**
   - **If the lesion disappears or fades upon stretching, it is likely leukoedema.**
2. **Eliminate Possible Local Irritants:**

    - **If frictional keratosis is suspected, assess for sharp teeth, parafunctional habits, or ill-fitting prostheses.**
3. **Follow-Up and Monitoring:**
    - **If the mucosal changes persist despite removing possible irritants, biopsy is recommended to rule out leukoplakia or dysplasia.**
4. **No Treatment Required if Leukoedema is Confirmed:**
    - **Reassure the patient, as it is a harmless mucosal variation.**

Conclusion

This image does not show any pathological lesions. The findings are most consistent with leukoedema, a benign mucosal condition. No treatment is necessary unless symptoms arise or the lesion changes over time. Would you like a differential guide for oral white lesions or biopsy indications for persistent mucosal changes?

CASE 2

Reference:

https://www.familydentalcare.com.au/wp-content/uploads/2024/10/young-cool-woman-joke-close-up-min-scaled-1.jpg

Chat-GPT4 answer:

Analysis and Probable Diagnosis

Observations:

1. **Normal tongue morphology**:
   - The tongue appears **pink, moist, and covered with evenly distributed papillae**.
   - No visible **ulcerations, white patches, erythematous areas, or nodules**.
2. **Well-defined fungiform and filiform papillae**:
   - The papillary pattern looks intact with no **atrophic or hypertrophic changes**.
3. **No visible signs of pathology**:
   - There are no apparent **leukoplakic, erythroplakic, ulcerative, or exophytic lesions**.
4. **Mild erythema on the lips**:
   - Slight redness on the lips, possibly due to **irritation, dehydration, or minor trauma**.

Most Probable Diagnosis: Clinically Normal Tongue

- The tongue appears to be within **normal physiological variation**, without any apparent pathological signs.

Differential Considerations (If Symptoms Are Present):

1. **Mild Dehydration or Irritation**:
   - If the patient reports a **dry sensation**, it may be due to **insufficient hydration, mouth breathing, or mild irritation**.
2. **Vitamin Deficiencies (B12, Iron, Folate)**:
   - If the patient complains of **burning sensation or glossitis**, a **nutritional deficiency should be considered**.
3. **Allergic or Irritant Contact Reactions**:
   - If associated with **burning, swelling, or tingling**, recent exposure to **spicy foods, toothpaste ingredients, or medications** should be assessed.
4. **Early Geographic Tongue (Erythema Migrans)**:
   - If the patient experiences **migrating, irregular depapillated areas**, it could indicate **benign migratory glossitis**.
5. **Burning Mouth Syndrome (If No Visible Changes but Symptoms Exist)**:
   - If the patient **feels discomfort without visible lesions**, a **neuropathic etiology** should be considered.

Recommended Next Steps (If Symptoms Exist):

- **Ask about symptoms**: Any pain, burning, taste changes, or dryness?
- **Check hydration and dietary history**: Is there a risk of dehydration or vitamin deficiency?
- **Monitor for changes**: If symptoms persist or new lesions appear, follow up with a more detailed clinical exam.

Conclusion

This tongue appears **clinically normal**, with no immediate pathological concerns. However, if the patient experiences **burning, pain, or functional disturbances**, further evaluation may be needed. Would you like guidance on **oral health recommendations or differential workups for burning mouth syndrome?**

CASE 3

Reference:
https://www.abdn.ac.uk/medical/elf/coursefiles/the-oral-cavity/1/img/10.jpg

Chat-GPT4 answer:

Analysis and Probable Diagnosis

Observations:

1. **Diffuse, slightly opalescent mucosa**:
   - The buccal mucosa appears **mildly whitish or translucent**, with no clear ulcerations or exophytic growths.
   - The lesion seems **homogeneous and does not show focal areas of hyperkeratosis or erosion**.
2. **No signs of ulceration, erythema, or exophytic mass**:
   - The surface is **smooth and well-defined**.
   - There are **no vascular changes, nodules, or suspicious thickened areas**.
3. **Symmetric Appearance (if present bilaterally)**:
   - If the same pattern is seen on the **contralateral buccal mucosa**, this suggests a **benign physiological or reactive condition**.

Most Probable Diagnosis: Leukoedema

- **Leukoedema** is a **benign variation of normal mucosa**, often presenting as a **diffuse, opalescent, or milky-white appearance**.
- It is **more common in darker-skinned individuals** and **smokers**.
- A key diagnostic feature: **Disappears or reduces in intensity when the mucosa is stretched** (stretch test).

Differential Diagnoses to Consider:

1. **Frictional Keratosis (If History of Chronic Trauma)**:
   - If the patient has a **habit of cheek biting, or dental irritation**, this could be **reactive keratosis**.
   - However, frictional keratosis is usually **more focal and rough-textured**, unlike leukoedema.
2. **Oral Lichen Planus - Reticular Type**:
   - Would present with **bilateral Wickham's striae (white lace-like pattern)**.
   - Biopsy is needed if the lesion **becomes erosive or symptomatic**.
3. **Early Leukoplakia (If Persistent and Asymmetric)**:
   - **Premalignant lesion**, does not disappear with stretching.
   - **Requires biopsy if the lesion persists >2 weeks**.
4. **Candidiasis (Unlikely Here)**:
   - White patches that **wipe off** with erythematous areas underneath.
   - More common in **immunosuppressed patients** or those on **antibiotics/steroids**.

Recommended Next Steps:

1. **Stretch Test**:
    - If the lesion disappears or fades, it is **likely leukoedema**.
2. **Eliminate Local Irritants**:
    - If frictional keratosis is suspected, assess for **sharp teeth, cheek-biting habits, or ill-fitting restorations**.
3. **Monitor for Changes**:
    - If the lesion is **persistent, asymmetric, or thickens over time**, biopsy is required to rule out **leukoplakia or dysplasia**.
4. **Reassurance if Leukoedema is Confirmed**:
    - **No treatment needed**, as it is a benign variant.
    - If the patient smokes, cessation is advised to prevent **mucosal alterations**.

Conclusion

This lesion is **most consistent with leukoedema**, a benign and physiological mucosal change. However, if the lesion **persists, thickens, or shows asymmetric growth, a biopsy should be performed**. Would you like guidance on **biopsy indications for leukoplakia or mucosal monitoring protocols**?

CASE 4

Reference:
https://www.abdn.ac.uk/medical/elf/coursefiles/the-oral-cavity/1/img/fom1.png

Chat-GPT4 answer

Analysis and Probable Diagnosis

Observations:

1. **Bilateral bluish-gray or brownish pigmentation on the ventral tongue and floor of the mouth**:
   - The pigmentations are **diffuse, irregular, and asymptomatic**.
   - No evidence of ulceration, nodularity, or tissue thickening.
2. **No associated inflammation or induration**:
   - The surrounding mucosa appears **healthy, with no signs of erythema or irritation**.
3. **Pattern suggests a physiological or benign process**:
   - The pigmentation is **symmetrical and follows a natural anatomical distribution**.

Most Probable Diagnosis: Physiologic Pigmentation (Melanotic Macules)

- This is a **benign** condition caused by **increased melanin production by basal keratinocytes and melanocytes**.
- More common in **individuals with darker skin tones** due to genetic predisposition.
- Typically appears in the **gingiva, buccal mucosa, hard palate, and ventral tongue**.

Differential Diagnoses to Consider:

1. **Amalgam Tattoo (Focal Blue-Black Pigmentation)**
   - Results from **dental material deposition** (amalgam particles embedded in tissue).
   - Typically **localized**, irregular, and may show **radiopaque fragments on X-ray**.
2. **Smoker's Melanosis (If History of Tobacco Use)**
   - Caused by **nicotine-induced melanin production**.
   - Found on the **anterior gingiva, buccal mucosa, and lips**.
3. **Drug-Induced Pigmentation (Minocycline, Antimalarials, Zidovudine)**
   - **Generalized mucosal discoloration** due to systemic medications.
   - Common culprits: **chloroquine, hydroxychloroquine, tetracyclines, and chemotherapy drugs**.
4. **Peutz-Jeghers Syndrome (If Perioral Pigmentation is Present)**
   - If the patient has **multiple mucocutaneous macules on the lips, oral cavity, and perioral skin**, this syndrome should be considered.
   - Associated with **gastrointestinal polyposis** and increased cancer risk.
5. **Addison's Disease (If Diffuse Mucosal and Skin Pigmentation)**
   - May present as **diffuse hyperpigmentation of oral mucosa, palms, and knuckles**.
   - Often accompanied by **fatigue, hypotension, and weight loss**.
6. **Melanoma (Rare but Requires Consideration)**
   - If the lesion **enlarges, becomes irregular, ulcerates, or is asymmetric**, biopsy is mandatory.

- Oral melanomas are **aggressive** but rare.

Recommended Next Steps:

1. **Clinical History and Risk Factor Assessment**:
   - Check for **smoking history, drug intake, trauma, or systemic conditions**.
   - If there are **other pigmented lesions on the skin or mucosa**, consider a syndromic cause.
2. **Observation and Monitoring**:
   - If the pigmentation is **symmetric, stable, and asymptomatic**, no intervention is needed.
   - If it **changes in size, color, or texture**, a biopsy is warranted.
3. **Biopsy Indications**:
   - **Irregular borders, asymmetry, ulceration, or rapid growth**.
   - **New-onset pigmentation with no obvious cause**.
   - If the lesion appears **nodular or irregular**, histopathologic examination is necessary to rule out **melanoma**.

Conclusion

This pigmentation is **most consistent with physiologic melanosis or smoker's melanosis** (if applicable). **No treatment is required unless there are suspicious changes**. Would you like a **detailed guide on differentiating benign vs malignant oral pigmentation or biopsy protocols for pigmented lesions?**

CASE 5

Reference:
https://www.lsusd.lsuhsc.edu/socs/images/floor%20of%20mouth.jpg

Chat-GPT4 answer

Analysis and Probable Diagnosis

Observations:

1. **Bilateral bluish-gray pigmentation on the floor of the mouth and ventral tongue**:
    - The pigmentations appear **diffuse and irregular, with a slightly mottled appearance**.
    - The surrounding mucosa is **intact, with no ulceration, nodularity, or induration**.
2. **Presence of Amalgam Restorations**:
    - The patient has **dental amalgam fillings**, which could suggest **a localized cause for pigmentation**.
3. **No Associated Inflammation or Erosion**:
    - The affected areas are **smooth, with no signs of active irritation, swelling, or erythema**.

Most Probable Diagnosis: Amalgam Tattoo

- Amalgam tattoos occur due to **incorporation of dental amalgam particles into the mucosa** following **restorative dental procedures or extractions**.
- The pigmentation is **bluish-gray to black**, non-wipeable, and typically **asymptomatic**.
- Often found in **gingiva, buccal mucosa, and floor of the mouth** near amalgam restorations.

Differential Diagnoses to Consider:

1. **Physiologic Pigmentation (Melanotic Macules)**
    - More common in individuals with **darker skin tones**.
    - Typically **symmetric and well-defined**, whereas amalgam tattoos are **more irregular**.
2. **Smoker's Melanosis**:
    - Occurs in long-term smokers due to **nicotine-induced melanin production**.
    - Commonly affects **anterior gingiva, buccal mucosa, and lips**.
3. **Drug-Induced Pigmentation**:
    - **Minocycline, antimalarials, and chemotherapeutic agents** can cause generalized mucosal discoloration.
    - Unlike amalgam tattoos, drug-induced pigmentation is usually **widespread and affects multiple sites**.
4. **Oral Melanoma (Rare but Serious)**
    - Suspicious features include **asymmetry, irregular borders, rapid growth, ulceration, or nodularity**.
    - Biopsy is mandatory if the lesion **changes in size, texture, or color**.

Recommended Next Steps:

1. **Radiographic Examination (Periapical X-ray or CBCT)**

    - If the lesion **contains radiopaque particles**, this confirms an **amalgam tattoo**.
2. **Clinical Monitoring**
    - If the pigmentation is **stable and asymptomatic**, no treatment is needed.
    - If the lesion **enlarges, changes color, or develops an irregular border**, a **biopsy is required**.
3. **Biopsy Indications**
    - If the lesion is **not associated with amalgam or dental restorations**.
    - If it **grows over time or appears irregularly pigmented**.
    - If melanoma is suspected, **histopathologic examination with immunohistochemistry (HMB-45, S100, Melan-A)** is necessary.

Conclusion

This pigmentation is **most consistent with an amalgam tattoo**, especially considering the presence of **amalgam restorations**. No intervention is required unless **changes in size, color, or texture occur**. Would you like a **detailed protocol for biopsy indications or management of oral pigmentation?**

CASE 6

Reference:
https://img.medscapestatic.com/pi/meds/ckb/58/6158tn.jpg

Chat-GPT4 answer

Analysis and Probable Diagnosis

Observations:

1. **Diffuse thickening of the palatal mucosa with multiple ridges**:
   - The mucosa of the **hard palate appears hyperkeratotic and corrugated**.
   - The texture is **rough, irregular, and nodular**, with no focal ulceration.
2. **Presence of Dental Amalgam Restorations**:
   - The patient has **dental amalgam restorations**, which may indicate **long-term exposure to oral irritants**.
3. **No Signs of Ulceration, Induration, or Malignant Changes**:
   - The lesion appears **benign**, without any **asymmetry, exophytic growth, or necrotic areas**.

Most Probable Diagnosis: Nicotine Stomatitis

- **Nicotine stomatitis (smoker's palate)** is a **benign, reactive condition** due to **chronic heat exposure**, often from **pipe smoking, cigars, or heavy cigarette use**.
- The palatal mucosa becomes **hyperkeratotic and inflamed**, leading to a **diffuse white, cobblestone-like appearance with inflamed minor salivary gland openings**.
- It is **not premalignant** but indicates chronic exposure to an **irritant (heat or chemicals)**.

Differential Diagnoses to Consider:

1. **Inflammatory Papillary Hyperplasia (If Associated with Dentures)**
   - Occurs in **denture wearers due to chronic irritation**.
   - Typically seen on the **hard palate beneath ill-fitting dentures**.
2. **Leukoplakia (If Localized and Persistent)**
   - If the lesion is **well-demarcated, thick, and does not regress after smoking cessation**, **biopsy is needed** to rule out **premalignant leukoplakia**.
3. **Verrucous Carcinoma (If Progressively Growing)**
   - A **slow-growing variant of squamous cell carcinoma**.
   - Appears as a **warty, thickened, white plaque that does not regress**.
4. **Oral Lichen Planus - Plaque Type (If Widespread and Symptomatic)**
   - Typically presents as **bilateral white striae or plaques**.
   - May have **burning sensation or ulcerative components**.

Recommended Next Steps:

1. **Patient History and Smoking Habits**:
   - Assess **smoking frequency, type of tobacco use (cigars, pipes, cigarettes), and duration**.

    - Ask about **other irritant exposures (hot beverages, spices, alcohol use)**.
2. **Smoking Cessation and Follow-Up**:
    - If due to smoking, **nicotine stomatitis should regress within 1-2 months after cessation**.
    - Persistent lesions require **biopsy to rule out leukoplakia or dysplasia**.
3. **Biopsy Indications**:
    - If the lesion **persists despite eliminating irritants**.
    - If there is **localized thickening, nodularity, ulceration, or asymmetry**.
    - If **verrucous carcinoma is suspected**.
4. **Preventive Recommendations**:
    - Encourage **smoking cessation** to reduce the risk of **oral cancer**.
    - Regular **oral cancer screenings** if the patient is a long-term smoker.

Conclusion

This lesion is **most consistent with nicotine stomatitis**, a **benign heat-induced keratosis**. **No immediate treatment is required, but smoking cessation is strongly advised**. If the lesion **persists or shows progressive thickening, biopsy should be performed**. Would you like guidance on **smoking cessation strategies or biopsy protocols for persistent oral keratoses?**

CASE 7

Reference:

https://media.springernature.com/full/springer-static/image/art%3A10.1038%2Fs41415-023-6138-5/MediaObjects/41415_2023_6138_Fig2_HTML.jpg

Chat-GPT4 answer:

Analysis and Probable Diagnosis

Observations:

1. **Generalized thickening and rugosity of the palatal mucosa**:
   - The **hard palate** presents with **corrugated ridges** and a **slightly erythematous appearance**.
   - The mucosa appears **firm, with no ulceration, nodularity, or exophytic growths**.
2. **No Signs of Malignancy or Induration**:
   - There is **no ulceration, bleeding, or mass effect**, which would indicate a **neoplastic process**.
3. **Possible Chronic Irritation or Mechanical Influence**:
   - The lesion appears to be **bilateral and symmetrical**, suggesting a **reactive or adaptive mucosal response**.

Most Probable Diagnosis: Inflammatory Papillary Hyperplasia (IPH)

- **Inflammatory papillary hyperplasia** is a **benign, reactive proliferation** of the palatal mucosa, commonly associated with:
  - **Chronic irritation from ill-fitting dentures or orthodontic appliances**.
  - **Mouth breathing or high palatal arch anatomy**.
  - **Poor oral hygiene and Candida colonization**.
- It presents as **erythematous, pebbled, or nodular thickening of the palatal mucosa** without ulceration or pain.

Differential Diagnoses to Consider:

1. **Nicotine Stomatitis (If the Patient is a Smoker)**:
   - Presents as **diffuse keratosis with inflamed minor salivary gland openings**.
   - Typically occurs due to **chronic heat exposure from smoking or hot beverages**.
2. **Denture Stomatitis (If Associated with a Removable Prosthesis)**:
   - Typically presents with **erythema under a denture base**, often due to **Candida infection**.
   - More common in **patients who wear dentures overnight**.
3. **Verrucous Hyperplasia (If Asymmetrical or Irregularly Thickened)**:
   - A **precancerous lesion** that may appear as **thickened, warty mucosa**.
   - If the lesion **persists, enlarges, or ulcerates, biopsy is recommended**.
4. **Palatal Keratosis (If Related to Chronic Friction or Irritation)**:

- Frictional keratosis from **orthodontic appliances, tongue habits, or parafunctional activity**.

Recommended Next Steps:

1. **Assess for Contributing Factors**:
   - History of **denture or orthodontic appliance use**.
   - Presence of **parafunctional habits (tongue thrusting, mouth breathing, bruxism)**.
   - Smoking or use of **hot beverages**.
2. **Improve Oral Hygiene and Remove Irritants**:
   - If associated with **dentures, adjusting the fit and improving hygiene is crucial**.
   - If Candida is suspected, an **antifungal treatment** (nystatin or fluconazole) may be needed.
3. **Biopsy if Needed**:
   - If the lesion is **progressive, asymmetric, or fails to resolve with management**, a biopsy should be performed to **rule out dysplasia or verrucous carcinoma**.
4. **Long-Term Monitoring**:
   - Regular follow-ups are important to **monitor for resolution or progression**.
   - If related to **denture use**, fabrication of a **well-fitting prosthesis** may be required.

Conclusion

This lesion is **most consistent with Inflammatory Papillary Hyperplasia (IPH)**, likely due to **chronic irritation from a dental prosthesis or mechanical factors**. **Management involves removing the underlying cause, improving hygiene, and monitoring for changes**. Would you like a **detailed protocol on antifungal therapy or prosthetic adjustments for hyperplastic palatal lesions?**

CASE 8

Reference:
https://www.researchgate.net/publication/348128138/figure/fig1/AS:976191007752194@1609753481777/ntraoral-examination-revealed-a-healthy-oral-mucosa.jpg

Chat-GPT4 answer:

Analysis and Probable Diagnosis

Observations:

1. **Localized erythema with slight keratotic changes on the buccal mucosa and vestibule**:
   - The area presents **diffuse redness with mild thickening of the mucosa**.
   - There is **no clear ulceration, nodularity, or exophytic growth**.
   - A faint **whitish or slightly keratotic area** is visible, possibly indicating **chronic irritation**.
2. **Location: Retromolar/Buccal Vestibule Region**:
   - This area is commonly affected by **chronic trauma, habits like cheek biting, or prosthetic irritation**.
3. **No Evidence of Ulceration, Induration, or Neoplastic Features**:
   - The lesion appears **flat or slightly raised but lacks an invasive component**.
   - No obvious **necrosis, rolled borders, or asymmetry**.

Most Probable Diagnosis: Frictional Keratosis or Traumatic Hyperkeratosis

- Caused by **chronic mechanical irritation**, such as:
  - **Cheek biting or parafunctional habits (bruxism, lip sucking, or tongue thrusting).**
  - **Irritation from rough dental restorations, prostheses, or malposed teeth.**
  - **Chronic exposure to spicy foods or repetitive trauma from mastication.**
- Typically presents as a **whitish or erythematous lesion that resolves after removal of the irritant**.

Differential Diagnoses to Consider:

1. **Lichen Planus (If There Are Reticular White Striae)**
   - If **bilateral Wickham's striae are present**, oral lichen planus should be considered.
   - Lichen planus is **chronic and may be symptomatic**, requiring biopsy if erosive.
2. **Leukoplakia (If the Lesion Persists Without an Obvious Cause)**
   - Leukoplakia is a **premalignant lesion** that appears **white, well-demarcated, and does not resolve with trauma removal**.
   - Biopsy is required if **persistent, thickened, or located in a high-risk site (floor of the mouth, ventral tongue, soft palate).**
3. **Erythroplakia (If the Red Area Persists Without Keratinization)**
   - A more serious consideration, as **erythroplakia has a higher risk of dysplasia or malignancy**.
   - Requires **biopsy if the lesion does not resolve within 2-3 weeks after removing irritants**.
4. **Oral Candidiasis (If the Lesion is Wipeable)**

- Candida infections, especially **chronic hyperplastic candidiasis**, can cause **white or erythematous mucosal changes**.
- If the patient is **immunocompromised, diabetic, or using inhaled steroids**, a fungal culture may be warranted.

Recommended Next Steps:

1. **Eliminate Possible Local Irritants**:
    - **Check for sharp cusps, defective restorations, or rough prostheses**.
    - Advise **behavioral modification if cheek biting or parafunctional habits are suspected**.
2. **Monitor for 2-3 Weeks**:
    - If the lesion resolves after removing the suspected irritant, no further intervention is needed.
    - If **persists, enlarges, or develops surface irregularities, biopsy is mandatory**.
3. **Biopsy Indications**:
    - If the lesion **remains after trauma removal**.
    - If it **thickens, ulcerates, or becomes indurated**.
    - If there is **no clear history of trauma** and the lesion is persistent.
4. **If Candida is Suspected**:
    - Consider **antifungal therapy (nystatin, fluconazole)** if the lesion has a white removable component.
    - A **fungal smear or culture** can confirm **candidiasis**.

Conclusion

This lesion is **most consistent with frictional keratosis or traumatic hyperkeratosis**, likely due to **chronic irritation from biting or dental trauma**. **Removing the source of irritation and monitoring for resolution** is the primary approach. If the lesion **persists beyond 3 weeks or worsens, biopsy is recommended**. Would you like **a protocol for distinguishing leukoplakia from benign keratosis or a biopsy guideline for persistent lesions?**

CASE 9

Reference:
https://t3.ftcdn.net/jpg/03/00/99/62/360_F_300996222_zpD4cR1vlEjBFmfatVALyd7Y1m7f6dGw.jpg

Chat-GPT4 answer

Analysis and Probable Diagnosis

Observations:

1. **Prominent and dilated veins on the ventral tongue**:
   - The **bluish-purple, linear, or tortuous vascular structures** on the ventral surface of the tongue are prominent.
   - No evidence of **ulceration, nodularity, or white plaques**.
2. **Symmetrical Distribution**:
   - The vascular prominence appears **bilateral and evenly distributed**, which is a key feature of benign vascular variations.
3. **No Associated Induration or Mass Effect**:
   - The tongue appears **smooth and moist, without any exophytic growths**.

Most Probable Diagnosis: Lingual Varicosities (Lingual Varices)

- **Lingual varices** are **dilated veins** on the **ventral surface of the tongue**, commonly seen in:
  - **Elderly individuals** due to **age-related vascular changes**.
  - **Hypertension** or **venous insufficiency**.
  - Patients with a history of **smoking**.
- They are **asymptomatic, benign, and do not require treatment**.

Differential Diagnoses to Consider:

1. **Sublingual Hemangioma (If Focal and Nodular)**
   - A **hemangioma** would present as a **raised, compressible, bluish lesion**.
   - Unlike varices, it may **enlarge over time** and sometimes **require surgical excision**.
2. **Venous Malformation (If Associated with Facial or Lip Involvement)**
   - If the vascular changes extend beyond the tongue (e.g., lips, oral mucosa), a **congenital venous malformation** should be considered.
3. **Oral Melanoma (If Pigmentation is Irregular and Asymmetric)**
   - Oral melanoma is **rare but serious**, typically presenting as a **progressive, asymmetric, darkly pigmented lesion** with **irregular borders**.
   - Unlike varices, melanoma **does not blanch with pressure**.
4. **Blue Nevus (If Well-Demarcated and Persistent)**
   - Blue nevi are **benign melanocytic lesions**, appearing as **small, well-circumscribed blue spots**.
   - Unlike lingual varices, they are **not vascular**.

Recommended Next Steps:

1. **No Treatment Needed (If Asymptomatic)**
    - Lingual varicosities are a **benign anatomical variation**, requiring no intervention.
2. **Clinical Monitoring**
    - If the **lesions change in size, become firm, or ulcerate**, further evaluation (including biopsy) may be necessary.
3. **Consider Cardiovascular Evaluation (If Extensive)**
    - In rare cases, **widespread venous dilations may be linked to systemic venous insufficiency or hypertension**.
    - If the patient has a history of **hypertension, smoking, or cardiovascular disease**, consider an evaluation.
4. **Biopsy Indications**
    - If the lesion is **solitary, nodular, enlarging, or has irregular pigmentation**.
    - If there is **bleeding, ulceration, or a non-blanching lesion**, biopsy should be performed to rule out **vascular malformations or melanoma**.

Conclusion

This case is **most consistent with lingual varicosities**, a **common and benign vascular variation** seen in **older adults and hypertensive individuals**. **No treatment is required unless symptomatic or suspicious changes occur**. Would you like a **differential guide for distinguishing vascular lesions or a biopsy protocol for suspicious oral pigmented lesions?**

<u>CASE 10</u>

<u>Reference:</u>
https://149629222.v2.pressablecdn.com/wp-content/uploads/Tongue-Tie.jpg

<u>Chat-GPT4 answer:</u>

The image shows **lingual varicosities**, a common benign vascular condition, typically observed in older adults.

Observations:

1. **Presence of Lingual Varicosities:**
   - Multiple **bluish to purplish, dilated, and tortuous veins** are visible on the **ventral surface of the tongue and floor of the mouth**.
   - The lesions appear non-tender, compressible, and without signs of inflammation, ulceration, or hemorrhage.
   - No signs of secondary complications such as **thrombosis, phlebolith formation, or mucosal ulceration**.
2. **Mucosal Integrity:**
   - The surrounding mucosa appears **intact, smooth, and adequately hydrated**, with no evidence of leukoplakia, erythroplakia, or dysplastic changes.
3. **Physiological Variations and Risk Factors:**
   - Lingual varicosities are often **asymptomatic and age-related**, with a higher prevalence in individuals over 50 years old.
   - Possible **associations with chronic venous insufficiency, hypertension, or vascular fragility**, but not necessarily indicative of systemic disease.

Recommended Next Steps:

- **No treatment is required** if asymptomatic.
- **Patient education:** Reassure the patient that **lingual varicosities are benign** and do not require intervention unless associated with discomfort or complications.
- **Monitoring:** Instruct the patient to report any sudden changes, such as **increased size, thrombosis, persistent pain, or ulceration**.
- **Further evaluation (if indicated):**
  - If the patient has a history of **systemic vascular disease, coagulation disorders, or unexplained oral discomfort**, consider a **vascular assessment**.
  - If associated with chronic venous insufficiency, referral for a **cardiovascular evaluation** may be warranted.

Conclusion:

This case is consistent with **lingual varicosities**, a **benign and age-related vascular phenomenon**, requiring **no intervention unless symptomatic**. Routine follow-up is advised only if changes occur over time.
