# Supplementary material for: Diagnostic Performance of ChatGPT-4o in Analyzing Oral Mucosal Lesions: A Comparative Study with Experts
Source: Medicina (Kaunas). 2025 Jul 30;61(8):1379. doi: 10.3390/medicina61081379 (PMC12388129; doi:10.3390/medicina61081379)
Supplement: Supplementary file 1 [file medicina-61-01379-s001.zip › Supplementary document S2.pdf]

Supplementary document S2: ChatGPT-4o responses on healthy oral mucosa – Case-by-Case analysis		
**Case number**	**ChatGPT4o diagnosis**	**Expert panel diagnosis**
1	Frictional keratosis	Normal
2	Normal	Normal
3	Normal	Normal
4	Normal	Normal
5	Amalgam tattoo	Normal
6	Nicotine stomatitis	Normal
7	Inflammatory papillary hyperplasia	Normal
8	Frictional keratosis	Normal
9	Lingual varices	Normal
10	Lingual varices	Normal
