# Supplementary material for: Diagnostic Performance of ChatGPT-4o in Analyzing Oral Mucosal Lesions: A Comparative Study with Experts
Source: Medicina (Kaunas). 2025 Jul 30;61(8):1379. doi: 10.3390/medicina61081379 (PMC12388129; doi:10.3390/medicina61081379)
Supplement: Supplementary file 1 [file medicina-61-01379-s001.zip › Supplementary document S3.pdf]

Supplementary document S3: ChatGPT-4o responses on oral mucosal lesions – Complete dataset.

CASE 1

Reference:
https://dentistchannel-online.s3.ap-south-1.amazonaws.com/photos/1661875002_Oral%20Leukoplakia.jpg

Chat-GPT4 answer:
The image shows the ventral surface of the tongue and floor of the mouth, with the presence of well-defined, bilateral, whitish, and reticulated plaques. The affected mucosa appears slightly thickened, with a corrugated or striated pattern, particularly in the anterior portion of the floor of the mouth and extending onto the ventral tongue.

Most Probable Diagnosis: Oral Lichen Planus (OLP)

Based on the clinical appearance, the most probable diagnosis is **Oral Lichen Planus (reticular type)**. The characteristic **white striae (Wickham striae)** and the symmetrical distribution strongly suggest this chronic inflammatory mucosal disorder.

Key Diagnostic Features:

- **White, lacy, or reticulated plaques** that are **bilateral** and **asymptomatic or mildly symptomatic**.
- Predominant involvement of **the ventral tongue and floor of the mouth**, common sites for lichen planus.
- **No evidence of ulceration or erosions**, making the **reticular form** of OLP more likely than erosive or atrophic variants.
- **Chronic, non-removable plaques**, ruling out conditions like pseudomembranous candidiasis.

Differential Diagnoses:

1. **Oral Leukoplakia:** Typically unilateral, well-demarcated, and lacks the characteristic Wickham striae.
2. **Hyperkeratotic Candidiasis:** May present with white patches, but is often associated with erythema and can be removed with scraping.
3. **Oral Graft-versus-Host Disease (GVHD):** Similar reticulated white patches but generally in post-transplant patients.
4. **Lupus Erythematosus:** Often presents with central atrophy or ulceration surrounded by white striations, commonly involving the vermilion border.

Next Steps for Confirmation:

- **Biopsy with histopathology**: Will show a **lichenoid inflammatory infiltrate** with **civatte bodies and basal cell degeneration**.
- **Direct Immunofluorescence (DIF)**: Helpful in differentiating from lupus erythematosus or GVHD.

Management:

- If asymptomatic: No active treatment required, but periodic monitoring for malignant transformation.
- If symptomatic: Topical **corticosteroids** (e.g., clobetasol propionate 0.05%) or calcineurin inhibitors (e.g., tacrolimus).
- **Malignant potential**: Although rare, regular follow-up is essential due to the potential risk of malignant transformation.

Conclusion:

The lesion in the image is highly suggestive of **reticular oral lichen planus**. Biopsy is recommended for definitive diagnosis, especially to rule out dysplasia or early malignancy in high-risk patients. Would you like a structured management algorithm or a literature review on OLP?

CASE 2

Reference:
https://www.ccjm.org/content/ccjom/87/3/133/F1.medium.gif

Chat-GPT4 answer:

The image shows a well-demarcated, extensive white lesion on the lateral border and ventral surface of the tongue. The lesion appears to have a non-homogeneous texture, with areas of hyperkeratosis and possible erosions. The presentation suggests a **potentially premalignant or malignant condition**.

Most Probable Diagnosis:

The lesion is highly suggestive of **oral leukoplakia with dysplasia** or possibly **oral squamous cell carcinoma (OSCC)**, given the irregular, non-homogeneous, and possibly indurated appearance.

Differential Diagnoses:

- **Oral candidiasis (hyperplastic type):** While candidiasis can present with white plaques, these would typically be removable with gauze. The fixed nature of this lesion makes candidiasis less likely.
- **Lichen planus (plaque-like form):** Less common on the lateral tongue, usually more reticular and symmetric.
- **Traumatic/frictional keratosis:** Possible if there is a history of chronic irritation (e.g., sharp teeth, dentures), but this lesion appears more suspicious for dysplasia.
- **Verrucous carcinoma:** A slow-growing, low-grade variant of OSCC with a warty surface.

Key Next Steps:

1. **Incisional Biopsy:** Essential to determine the degree of dysplasia or presence of malignancy.
2. **Histopathological Examination:** To confirm the nature of the lesion (hyperkeratosis, dysplasia, carcinoma).
3. **Eliminate Risk Factors:** If the patient has a history of smoking, alcohol use, or betel quid chewing, cessation is crucial.
4. **Imaging (CT/MRI if OSCC suspected):** To assess local invasion and lymph node involvement.
5. **Regular Follow-Up:** If precancerous, close monitoring is needed to detect malignant transformation early.

Would you like to discuss biopsy techniques, management options, or follow-up protocols for leukoplakia and OSCC?

CASE 3

Reference:
https://www.chirurgiaorale.it/wp-content/uploads/2019/07/Epulide-Prof.-Luigi-Checchi-1.jpeg

Chat-GPT4 answer:

The image shows a well-circumscribed, red, vascular-appearing nodular lesion on the attached gingiva in the anterior maxillary region. The lesion appears to be exophytic, erythematous, and possibly ulcerated, with surrounding mild inflammation.

Most Probable Diagnosis:

The lesion is most consistent with a **pyogenic granuloma**, a benign, reactive lesion commonly found on the gingiva due to chronic irritation, trauma, or hormonal changes (e.g., pregnancy). It is characterized by rapid growth, a high degree of vascularity, and a tendency to bleed easily.

Differential Diagnoses:

- **Peripheral giant cell granuloma (PGCG):** Similar in appearance but often has a bluish-purple hue and may show bone resorption radiographically.
- **Peripheral ossifying fibroma (POF):** A firm, fibrous lesion that may contain calcifications; less vascular than a pyogenic granuloma.
- **Squamous cell carcinoma:** Less likely, but persistent ulcerated lesions should be biopsied to rule out malignancy.
- **Metastatic lesion:** Rare but should be considered, especially if the patient has a history of malignancy.

Recommended Next Steps:

1. **Clinical history:** Assess for local irritants (e.g., plaque, calculus, trauma, orthodontic appliances).
2. **Biopsy:** If the lesion is large, recurrent, or atypical in presentation.
3. **Surgical excision:** Complete removal, including the base, to reduce recurrence.
4. **Histopathological examination:** To confirm the diagnosis and rule out other lesions.
5. **Oral hygiene reinforcement:** To prevent recurrence and ensure good periodontal health.

Would you like to discuss surgical removal techniques or adjuvant therapies (e.g., laser excision)?

CASE 4

Reference:
https://i0.wp.com/idmperu.com/wp-content/uploads/2021/12/01-78.jpg?fit=820%2C355&ssl=1

Chat-GPT4 answer:

The image shows a well-defined, symmetrical swelling on the midline of the hard palate, covered by intact, normal-colored mucosa without ulceration, erythema, or significant keratosis.

Most Probable Diagnosis:

The lesion appears consistent with a **torus palatinus**, a benign, bony exostosis that commonly arises in the midline of the hard palate. This condition is asymptomatic in most cases and does not require treatment unless it interferes with prosthetic rehabilitation (e.g., denture fitting) or becomes ulcerated due to trauma.

Differential Diagnoses:

- **Minor salivary gland tumor** (e.g., pleomorphic adenoma, mucoepidermoid carcinoma): These would typically present as unilateral, firm, slow-growing masses rather than a bilateral, midline torus.
- **Fibrous hyperplasia**: Could develop in response to chronic irritation, but it would usually lack the characteristic bony hard consistency of a torus.
- **Osteoma**: A solitary osteoma could occur but would be more discrete rather than a broad-based, midline exostosis.
- **Maxillary cysts or neoplasms**: A rare consideration, but odontogenic or non-odontogenic cysts (e.g., nasopalatine duct cyst) could appear as palatal swellings, typically with associated radiographic findings.

Recommended Next Steps:

1. **Clinical palpation**: To confirm the bony consistency typical of a torus palatinus.
2. **Radiographic evaluation (CBCT/Panoramic X-ray)**: If needed to confirm the presence of a bony outgrowth and rule out intraosseous pathology.
3. **Monitoring**: If asymptomatic, no treatment is necessary.
4. **Surgical removal**: Only indicated in cases of recurrent trauma, interference with prosthetic devices, or significant patient discomfort.

CASE 5

Reference:
https://www.researchgate.net/publication/322046926/figure/fig3/AS:726161374076929@1550141771789/Oral-squamous-cell-carcinoma-left-lateral-tongue.jpg

Chat-GPT4 answer:

The image shows an ulcerative-proliferative lesion on the lateral border of the tongue, with areas of leukoplakia-like keratotic changes and erythematous, possibly indurated regions. The lesion appears irregular and heterogeneous, with evidence of possible necrotic tissue or secondary infection.

Most Probable Diagnosis:

Given the macroscopic appearance, the most likely diagnosis is **oral squamous cell carcinoma (OSCC)**. The lateral border of the tongue is a high-risk site for malignant transformation, especially in patients with risk factors such as tobacco and alcohol use, chronic trauma, or HPV infection.

Differential Diagnoses:

- **Oral leukoplakia with dysplasia**: If this lesion has a history of a pre-existing white patch, it could represent leukoplakia that has undergone malignant transformation.
- **Oral lichen planus (erosive form)**: Although less likely, erosive lichen planus can present with ulcerations and erythema but typically has a more symmetrical, reticular pattern.
- **Traumatic ulcer or chronic traumatic ulcerative granuloma (TUGSE)**: Unlikely unless there is a clear history of repeated trauma.
- **Infectious etiologies**: Tuberculous ulcer, deep fungal infections (e.g., histoplasmosis), or syphilitic chancre could mimic malignancy, but they are rarer.

Recommended Next Steps:

1. **Biopsy (Incisional)**: To confirm histopathological diagnosis and assess for dysplasia or invasive carcinoma.
2. **Imaging (CT/MRI with contrast)**: To evaluate for local invasion and nodal involvement.
3. **HPV Testing**: If clinical suspicion is high, particularly in younger patients or non-smokers.
4. **Full Head and Neck Examination**: To assess for other lesions, lymphadenopathy, and potential field cancerization.

CASE 6

Reference:
https://marianiumberto.it/wp-content/uploads/2018/09/Angiomi/image16.jpeg

Chat-GPT4 answer:

The image shows a well-circumscribed, red, vascular-appearing nodular lesion on the attached gingiva in the anterior maxillary region. The lesion appears to be exophytic, erythematous, and possibly ulcerated, with surrounding mild inflammation.

Most Probable Diagnosis:

The lesion is most consistent with a **pyogenic granuloma**, a benign, reactive lesion commonly found on the gingiva due to chronic irritation, trauma, or hormonal changes (e.g., pregnancy). It is characterized by rapid growth, a high degree of vascularity, and a tendency to bleed easily.

Differential Diagnoses:

- **Peripheral giant cell granuloma (PGCG):** Similar in appearance but often has a bluish-purple hue and may show bone resorption radiographically.
- **Peripheral ossifying fibroma (POF):** A firm, fibrous lesion that may contain calcifications; less vascular than a pyogenic granuloma.
- **Squamous cell carcinoma:** Less likely, but persistent ulcerated lesions should be biopsied to rule out malignancy.
- **Metastatic lesion:** Rare but should be considered, especially if the patient has a history of malignancy.

Recommended Next Steps:

1. **Clinical history:** Assess for local irritants (e.g., plaque, calculus, trauma, orthodontic appliances).
2. **Biopsy:** If the lesion is large, recurrent, or atypical in presentation.
3. **Surgical excision:** Complete removal, including the base, to reduce recurrence.
4. **Histopathological examination:** To confirm the diagnosis and rule out other lesions.
5. **Oral hygiene reinforcement:** To prevent recurrence and ensure good periodontal health.

CASE 7

Reference:
https://upload.wikimedia.org/wikipedia/commons/4/4b/Ranula_human_09.jpg

Chat-GPT4 answer:

The image shows a **large, well-circumscribed, bluish, fluid-filled swelling** on the **floor of the mouth**, lateral to the lingual frenulum. The lesion appears fluctuant, with visible vascularization and a dome-shaped morphology.

Most Probable Diagnosis:

This lesion is highly suggestive of a **ranula**, a **mucous retention cyst** arising from the **sublingual gland** due to ductal obstruction or rupture, leading to extravasation of saliva into surrounding tissues.

- **Simple ranula:** Confined to the floor of the mouth.
- **Plunging ranula:** Extends beyond the mylohyoid muscle, presenting as a swelling in the submandibular region.

Differential Diagnoses:

- **Mucocele:** More common on the lower lip; however, ranulas are a specific form of mucocele found in the floor of the mouth.
- **Salivary gland duct cyst:** A true cystic structure lined by epithelium, unlike ranulas, which are pseudocysts.
- **Vascular malformation (e.g., venous or lymphatic malformation):** Bluish and compressible, but these typically do not arise suddenly or fluctuate in size.
- **Dermoid/Epidermoid cyst:** Midline and firmer, rather than fluctuant and bluish.
- **Lipoma:** Soft, yellowish rather than bluish, and not fluid-filled.

Key Next Steps:

1. **Clinical examination:** Palpation to confirm fluctuation and transillumination (if necessary).
2. **Imaging (Ultrasound or MRI):** To determine extension and confirm the diagnosis, particularly in suspected **plunging ranula** cases.
3. **Fine-needle aspiration (FNA):** Can be performed if diagnosis is uncertain.
4. **Definitive treatment:**
    - **Marsupialization** (for smaller lesions)
    - **Excision of the ranula and sublingual gland** (to prevent recurrence)
    - **Sclerotherapy** (alternative for non-surgical management)

CASE 8

Reference:
https://curaprox.us/modules/advancedcms/uploads/AdvancedCmsBlockTeaser/177/xgingivitis-detail.webp.pagespeed.ic.nqbS_wHVca.webp

Chat-GPT4 answer:

The image shows **generalized gingival inflammation**, characterized by erythema (redness), swelling, and signs of bleeding along the gingival margins, particularly in the upper and lower anterior regions.

Most Probable Diagnosis:

This presentation is highly suggestive of **gingivitis**, which is an inflammatory response of the gingival tissues to bacterial plaque accumulation.

Differential Diagnoses:

- **Gingivitis due to plaque accumulation:** The most common cause, associated with poor oral hygiene.
- **Hormonal gingivitis:** Can be seen in pregnancy, puberty, or due to oral contraceptive use.
- **Gingivitis due to systemic conditions:** Such as diabetes or vitamin C deficiency (scurvy).
- **Gingival enlargement (drug-induced):** Certain medications (e.g., phenytoin, cyclosporine, calcium channel blockers) can cause overgrowth.
- **Necrotizing ulcerative gingivitis (NUG):** If associated with pain, ulceration, and halitosis.
- **Autoimmune conditions (lichen planus, pemphigoid, pemphigus):** If accompanied by mucosal erosions or desquamation.

Recommended Next Steps:

1. **Oral hygiene reinforcement:** Emphasize brushing twice daily with fluoride toothpaste and daily flossing.
2. **Professional dental cleaning (scaling and root planing):** To remove plaque and calculus.
3. **Chlorhexidine mouthwash (0.12-0.2%):** Short-term use to reduce bacterial load.
4. **Evaluation for systemic conditions:** If inflammation persists despite good oral hygiene.
5. **Follow-up after 2-3 weeks:** To assess improvement and determine if further periodontal treatment is necessary.

CASE 9

Reference:
https://www.aaom.com/assets/condition-information/oral%20lichen%20planus%201.jpg

Chat-GPT4 answer:

The lesion is highly suggestive of **oral lichen planus (OLP), reticular form**. This chronic, immune-mediated condition affects the oral mucosa and presents as **Wickham's striae** (fine white reticulations) on the buccal mucosa, tongue, or gingiva.

Differential Diagnoses:

- **Leukoplakia:** Usually presents as a well-demarcated white patch with a higher risk of dysplasia.
- **Oral candidiasis (pseudomembranous or hyperplastic):** Can mimic OLP but is usually removable with gauze.
- **Lupus erythematosus (oral manifestations):** May present with similar striations but with erythematous and ulcerative components.
- **Graft-versus-host disease (GVHD):** If the patient has a history of hematopoietic stem cell transplantation, it can mimic OLP.
- **Oral lichenoid reaction:** Similar to OLP but usually due to drugs, dental materials, or systemic conditions.

Key Next Steps:

1. **Clinical correlation:** Assess for cutaneous lesions, as lichen planus can affect the skin (pruritic, purple, polygonal papules).
2. **Histopathology (biopsy):** To confirm the diagnosis and rule out dysplasia or lichenoid dysplasia.
3. **Regular follow-up:** OLP has a **low but real risk of malignant transformation** (~1-2%).
4. **Management:**
   - If asymptomatic: No treatment needed, but regular monitoring.
   - If symptomatic (erosive/atrophic forms): **Topical corticosteroids** (e.g., clobetasol propionate 0.05% gel).
   - **Systemic therapy:** In refractory cases, immunomodulators (e.g., tacrolimus) may be considered.

CASE 10

Reference:
https://marianiumberto.it/wp-content/uploads/2018/09/Mucocele/mucocele8.jpeg

Chat-GPT4 answer:

The image shows a **well-circumscribed, dome-shaped, fluid-filled lesion** on the lower labial mucosa. The lesion appears **translucent, bluish, and fluctuant**, indicating the presence of mucin beneath the epithelium.

Most Probable Diagnosis:

The lesion is highly suggestive of a **mucocele**, a **mucous extravasation cyst** resulting from trauma or obstruction of minor salivary gland ducts. This occurs due to **rupture of a salivary gland duct**, leading to mucin accumulation in surrounding tissues.

Differential Diagnoses:

- **Retention cyst (true salivary duct cyst):** Unlike mucoceles, these are lined by epithelium.
- **Fibroma:** More firm and not fluid-filled.
- **Vesiculobullous disorder (e.g., pemphigus, mucous membrane pemphigoid):** Usually multiple, with ulceration upon rupture.
- **Lipoma:** Yellowish rather than translucent, and firmer in consistency.
- **Minor salivary gland tumor (e.g., mucoepidermoid carcinoma):** Persistent, non-fluctuant, and may have an irregular surface.

Key Next Steps:

1. **Clinical history:** Evaluate for recurrent trauma (e.g., lip biting).
2. **Observation:** Some mucoceles resolve spontaneously.
3. **Definitive treatment:**
   - **Surgical excision** with removal of the associated minor salivary gland (to prevent recurrence).
   - **Laser ablation** as an alternative.
4. **Histopathological examination:** To confirm the diagnosis, particularly if the lesion is recurrent or atypical.

CASE 11

Reference:
https://ars.els-cdn.com/content/image/1-s2.0-S001185241630023X-gr1.jpg

Chat-GPT4 answer:

The image shows a **well-circumscribed, firm, dome-shaped swelling** on the midline of the **hard palate**, covered by intact mucosa with visible vascularization.

Most Probable Diagnosis:

This lesion is most suggestive of a **palatal torus (torus palatinus)**, a benign, bony exostosis arising from the midline of the hard palate.

Differential Diagnoses:

- **Salivary gland tumor (e.g., pleomorphic adenoma, mucoepidermoid carcinoma):** Would usually present as an asymptomatic, slow-growing submucosal mass but is not typically **midline**.
- **Fibrous hyperplasia:** Could occur due to chronic irritation but would be softer in consistency.
- **Nasopalatine duct cyst:** Typically occurs in the anterior midline but is more likely to be **radiolucent on imaging** and may cause **fluctuant swelling**.
- **Osteoma:** A benign bony growth, but torus palatinus is more common in this location.
- **Squamous cell carcinoma:** Less likely given the intact epithelium and well-defined borders.

Key Next Steps:

1. **Clinical palpation:** The lesion should feel hard and non-compressible, confirming a bony consistency.
2. **Radiographic evaluation (CBCT, panoramic X-ray):** To confirm the **bony** nature of the lesion.
3. **Observation:** No treatment is required unless it interferes with speech, eating, or denture placement.
4. **Surgical excision:** If symptomatic or causing functional issues.

CASE 12

Reference:
https://www.bioral.it/main3/images/docs/Correnti/tatuaggio.jpg

Chat-GPT4 answer:

The image shows a **bluish-gray macular discoloration** on the **posterior buccal mucosa and gingiva**, adjacent to dental restorations.

Most Probable Diagnosis:

The most likely diagnosis is an **amalgam tattoo**, which results from the accidental implantation of **dental amalgam particles** into the soft tissues. These tattoos are often seen near **posterior restorations**, particularly in areas where dental work has been performed.

Differential Diagnoses:

- **Physiologic pigmentation (racial melanosis):** More diffuse and typically found in patients with higher baseline melanin production.
- **Melanotic macule:** A well-defined, flat, brown or black lesion, more common on the lips and gingiva.
- **Oral melanocytic nevus:** A localized pigmented lesion, but typically **not associated with restorations**.
- **Oral melanoma:** Rare, but must be considered if the lesion is enlarging, irregular, or has heterogeneous pigmentation.
- **Heavy metal pigmentation (e.g., lead, bismuth, or arsenic):** More widespread in distribution.

Key Next Steps:

1. **Clinical history:** Confirm recent or past placement/removal of amalgam fillings.
2. **Palpation:** The lesion should be non-elevated and asymptomatic.
3. **Radiographic imaging (periapical X-ray):** To check for **radio-opaque amalgam particles** embedded in the tissue.
4. **Biopsy:** If the lesion is **irregular, growing, or lacks a clear history of amalgam exposure**, a biopsy may be required to **rule out melanoma** or other pigmented lesions.

Management:

- **No treatment necessary** if the diagnosis is clear and the lesion is asymptomatic.
- **Excisional biopsy** if there is diagnostic uncertainty or cosmetic concerns.

CASE 13

Reference:
https://opmdcare.com/wp-content/uploads/oral-gvhd-image1-1024x768.jpeg

Chat-GPT4 answer:

The image shows **white, irregular, and keratotic plaques with surrounding erythema** on the **buccal mucosa**. The lesions appear to have a somewhat reticular pattern and are **not easily scrapable**, suggesting a chronic inflammatory or premalignant condition.

Most Probable Diagnosis:

The presentation is highly suggestive of **oral lichen planus (OLP), erosive or atrophic form**. This is a **chronic immune-mediated mucosal disorder** characterized by **Wickham's striae (white reticulations)**, sometimes accompanied by **erythema and ulceration** in more severe cases.

Differential Diagnoses:

- **Oral leukoplakia:** If the lesion is persistent, white, and non-scrapable, leukoplakia should be considered, particularly given the risk of malignant transformation.
- **Oral candidiasis (chronic hyperplastic type):** Unlike OLP, candidiasis lesions may be partially removable with gauze.
- **Oral lichenoid reaction:** A localized lesion with a similar appearance, often due to **contact hypersensitivity to dental materials** or medications.
- **Pemphigus vulgaris or mucous membrane pemphigoid:** If there is a history of blistering or desquamative gingivitis.
- **Erythroleukoplakia:** A high-risk **premalignant lesion** that appears as mixed red and white areas.
- **Lupus erythematosus (oral manifestation):** Can mimic OLP but usually occurs with systemic symptoms.

Key Next Steps:

1. **Biopsy (incisional)**: To confirm the diagnosis and rule out **dysplasia or malignancy**, especially in erosive/ulcerated areas.
2. **Histopathological evaluation:** Characteristic features of OLP include **liquefaction degeneration of the basal layer and a band-like inflammatory infiltrate**.
3. **Topical corticosteroids (e.g., clobetasol 0.05% gel, dexamethasone mouth rinse):** First-line treatment for symptomatic cases.
4. **Regular follow-up:** To monitor for **malignant transformation** (1-2% risk over time).
5. **Eliminate potential triggers:** If lichenoid reaction is suspected, **assess for medication or dental material-related causes**.

Management Considerations:

- **Mild asymptomatic cases:** Observation and good oral hygiene.

- **Symptomatic or erosive cases:** Topical corticosteroids, immunosuppressants (e.g., tacrolimus for refractory cases).
- **Biopsy and long-term monitoring:** If the lesion persists or shows changes suggestive of malignant transformation.

CASE 14

Reference:
https://marianiumberto.it/wp-content/uploads/2022/08/HHT-1.jpg

Chat-GPT4 answer:

The image shows **multiple erythematous, ulcerative, and papular lesions scattered across the dorsal surface of the tongue**. The lesions appear inflamed, and some have **crusted or scab-like surfaces**, suggesting a chronic or recurrent condition.

Most Probable Diagnosis:

The presentation is highly suggestive of **lingual herpes simplex virus (HSV) infection**, specifically **recurrent herpes simplex or herpetic stomatitis**. HSV can present as **painful, grouped vesicles that ulcerate**, typically on the tongue, gingiva, and lips.

Differential Diagnoses:

- **Aphthous stomatitis (canker sores):** Typically larger, solitary, and with a **yellowish center** and erythematous halo.
- **Erythema multiforme (EM):** If associated with recent infections or medications, EM can present with **multiple oral ulcers** and possibly **skin lesions (target lesions)**.
- **Hand, foot, and mouth disease (Coxsackievirus A):** Typically seen in children, with oral vesicles and accompanying **palmoplantar rash**.
- **Candidiasis (pseudomembranous or erythematous form):** White plaques (if pseudomembranous) or atrophic red areas, but typically not ulcerative.
- **Secondary syphilis (mucous patches):** If associated with systemic symptoms and a history of sexual exposure.
- **Oral lichen planus (erosive form):** If lesions have **reticular white striations (Wickham's striae)**, which are absent here.

Key Next Steps:

1. **Clinical history:**
   - Any recent viral illnesses, fever, or reactivation episodes?
   - History of **HSV-1 infections (cold sores)?**
2. **Tzanck smear or PCR testing for HSV:** To confirm the presence of **HSV-infected multinucleated giant cells**.
3. **Management:**
   - **Topical or systemic antiviral therapy** (e.g., acyclovir, valacyclovir) to shorten duration if diagnosed early.
   - **Analgesics or topical anesthetics** (e.g., lidocaine) for pain management.
   - **Supportive care:** Hydration, soft diet, and avoidance of irritants.

Would you like to discuss **antiviral treatment protocols, HSV recurrence prevention, or differential diagnostic tests**?

CASE 15

Reference:
https://img.perioimplantadvisory.com/files/base/ebm/pia/image/2019/01/16x9/content_dam_diq_online_articles_2016_09_16sept15piadipace_t.png?auto=format,compress&fit=fill&fill=blur&w=1200&h=630

Chat-GPT4 answer:

The image shows **extensive necrotic bone exposure in the posterior mandible**, surrounded by **inflamed mucosa** with evidence of **sequestration and necrosis**. Some adjacent teeth also show signs of decay and possible infection.

Most Probable Diagnosis:

This presentation is highly suggestive of **medication-related osteonecrosis of the jaw (MRONJ)**, particularly **osteonecrosis associated with bisphosphonates or denosumab**. The presence of **exposed necrotic bone**, persistent inflammation, and lack of healing suggests a **chronic osteonecrotic process**.

Differential Diagnoses:

- **Osteoradionecrosis (ORN):** If the patient has a history of radiation therapy to the head and neck.
- **Chronic suppurative osteomyelitis:** Associated with deep infections, but usually has more surrounding soft tissue swelling and purulent discharge.
- **Primary or metastatic malignancy (e.g., squamous cell carcinoma, multiple myeloma, or metastases):** If there is progressive bone destruction without a history of antiresorptive therapy.
- **Severe periodontitis with secondary bone loss:** Could contribute, but necrotic bone exposure is not typical.
- **Syphilitic or tuberculous osteomyelitis:** Rare but can mimic osteonecrosis.

Key Next Steps:

1. **Clinical history:**
    - Use of **bisphosphonates, denosumab, or antiangiogenic agents**?
    - History of **tooth extractions, trauma, or dental procedures**?
    - Any previous **head and neck radiation therapy**?
2. **Imaging (CBCT, panoramic X-ray, or MRI):**
    - To assess **extent of bone necrosis** and rule out osteomyelitis or malignancy.
3. **Microbiological culture (if secondary infection is suspected).**
4. **Histopathological biopsy (if malignancy is suspected).**
5. **Management options:**
    - **Stage-dependent treatment:** Conservative management (chlorhexidine rinses, antibiotics if infected) versus surgical **sequestrectomy or resection** in advanced cases.
    - **Avoid surgical debridement unless necessary**, as it can exacerbate bone exposure.

- **Pain control and infection management** with broad-spectrum antibiotics (amoxicillin ± metronidazole).

Important Considerations:

- If the patient is on **denosumab**, discontinuation may be considered in consultation with the prescribing physician.
- **Preventive dental measures** are critical before initiating bisphosphonate or denosumab therapy.

CASE 16

Reference:
https://static.tecnichenuove.it/ildentistamoderno/2015/04/DM_2012_001_gramegna001.jpg

Chat-GPT4 answer:

Clinical Observations:

1. **Ulcerated, Necrotic, and Infiltrative Lesion of the Palatal Mucosa**
    - The lesion is characterized by **a large, ulcerated area with central necrosis and irregular, infiltrative margins**.
    - The **black necrotic center** suggests **tumor necrosis and possible secondary infection or hemorrhage**.
    - The surrounding mucosa appears **erythematous and indurated**, indicative of **a locally aggressive process**.
2. **Irregular Borders and Deep Invasion**
    - The lesion's **ill-defined and infiltrative nature** raises **strong suspicion for an advanced squamous cell carcinoma (SCC)**.
    - SCC of the palate is typically associated with **chronic tobacco or alcohol use, ill-fitting dentures, or HPV infection**.

Primary Differential Diagnosis:

- **1. Squamous Cell Carcinoma (SCC) (Most Likely Diagnosis)**
    - SCC is the **most common malignant tumor of the oral cavity**, often presenting as **an ulcerated lesion with rolled, indurated margins and deep invasion**.
    - The presence of **central necrosis** suggests **tumor ischemia, which is common in rapidly growing malignancies**.
    - SCC of the **hard palate** can invade **bone (maxilla) and adjacent soft tissues**, leading to **oroantral fistula formation** in advanced cases.
- **2. Mucosal Malignant Melanoma (MMM) (Less Likely but Must Be Ruled Out)**
    - The **black pigmentation** could indicate **melanoma**, which is known for its **asymmetric, ulcerated, and rapidly spreading nature**.
    - However, SCC with **hemorrhagic necrosis and tissue infarction** can mimic pigmented lesions.
- **3. Necrotizing Deep Fungal Infection (e.g., Mucormycosis in Immunocompromised Patients)**
    - If the patient is **diabetic or immunosuppressed**, **mucormycosis** should be considered.
    - Unlike SCC, **fungal infections progress rapidly and may cause palatal perforation**.
- **4. Aggressive Lymphoproliferative Disorder (e.g., Extranodal NK/T-cell Lymphoma, Nasal Type)**
    - Epstein-Barr virus (EBV)-associated lymphomas can present as **ulcerative, necrotic lesions of the palate**, but are less common.

Recommended Next Steps:

1. Urgent Biopsy with Histopathological and Immunohistochemical Analysis

- **Incisional biopsy with deep tissue sampling** to confirm SCC and assess depth of invasion.
- **Immunohistochemistry panel for differentiation:**
    - **SCC markers**: p63, cytokeratin CK5/6, CK14.
    - **Melanoma markers (to exclude melanoma)**: S-100, HMB-45, Melan-A.
    - **Fungal stains (PAS, GMS) if infection is suspected**.

2. Imaging for Tumor Staging

- **Contrast-enhanced MRI or CT scan** of the head and neck to assess **local tissue invasion (bone, maxilla, sinuses, soft tissue)**.
- **PET-CT scan or neck CT** to evaluate for **regional lymph node or distant metastases**.

3. Multidisciplinary Tumor Board Consultation

- Referral to **head and neck oncology, maxillofacial surgery, and radiation oncology**.
- If confirmed SCC, discussion of **surgical resectability and need for adjuvant therapy**.

4. Definitive Treatment Plan (Based on Staging and Tumor Extent)

- **Early-stage SCC (T1-T2, N0)**:
    - **Radical surgical excision with clear margins**, considering maxillectomy if bone invasion is present.
- **Advanced-stage SCC (T3-T4, N+)**:
    - **Surgical resection + neck dissection (if nodal involvement is confirmed)**.
    - **Adjuvant radiotherapy ± concurrent chemotherapy (if margins are positive or extracapsular spread is detected)**.

Conclusion:

- **The clinical presentation strongly suggests an advanced squamous cell carcinoma (SCC) of the hard palate**, with necrosis and possible bone invasion.
- **Urgent biopsy and imaging are necessary to confirm the diagnosis and determine staging**.
- **If SCC is confirmed, a multimodal treatment approach (surgery, radiation, chemotherapy) will be required depending on tumor extent**.
- **A prompt oncologic evaluation is critical, as advanced SCC carries a significant risk of local destruction and metastasis**.

CASE 17

Reference:

https://ars.els-cdn.com/content/image/1-s2.0-S2667147622000140-gr1.jpg

Chat-GPT4 answer:

The image shows a **large, ulcerative, and exophytic lesion on the lateral border of the tongue**, with **irregular, non-homogeneous white and erythematous areas**, and **induration**.

Most Probable Diagnosis:

The lesion is highly suggestive of **oral squamous cell carcinoma (OSCC)**. The **lateral border of the tongue** is a high-risk site for **malignant transformation**, especially in individuals with **tobacco, alcohol, betel quid use, or HPV exposure**.

Differential Diagnoses:

- **Oral leukoplakia with dysplasia:** If the lesion started as a white patch, it could have undergone malignant transformation.
- **Traumatic ulcer/granuloma:** If there is a history of chronic irritation (e.g., sharp tooth, dentures), but these lesions typically heal within **2 weeks**.
- **Erosive lichen planus:** Usually has **bilateral reticular striations (Wickham's striae)** and more widespread distribution.
- **Chronic hyperplastic candidiasis:** White, non-scrapable lesion, but lacks the ulcerated and infiltrative nature seen here.
- **Syphilitic ulcer (tertiary syphilis):** Could mimic OSCC but is associated with systemic findings.

Key Next Steps:

1. **Urgent incisional biopsy:** To confirm OSCC and determine histopathological grade.
2. **Imaging (CT/MRI with contrast):** To assess **local invasion and lymph node involvement**.
3. **HPV testing:** If suspected (especially in non-smokers).
4. **Staging (TNM classification):** To guide treatment planning.
5. **Multidisciplinary team approach:** Head and neck oncology, maxillofacial surgery, radiation oncology.

Management:

- **Early-stage (T1-T2, N0):** Surgical excision with negative margins.
- **Locally advanced (T3-T4 or N+):** Surgery + adjuvant **radiotherapy ± chemotherapy**.
- **Unresectable/metastatic cases:** Consider **immunotherapy (PD-1 inhibitors like pembrolizumab) or targeted therapy**.

CASE 18

Reference:
https://encrypted-tbn0.gstatic.com/images?q=tbn:ANd9GcSzYTrGus780r757OP38YujuctMUH0jTmaizg&s
https://www.ccjm.org/content/ccjom/87/3/133/F1.medium.gif

Chat-GPT4 answer:

The image shows **a pronounced recession of the tongue toward the floor of the mouth, with limited mobility and fibrotic tissue bands anchoring it**. This is most consistent with **oral submucous fibrosis (OSMF)**, a chronic, progressive, and potentially malignant disorder associated with **betel quid, areca nut chewing, or chronic irritation**.

Most Probable Diagnosis:

Oral submucous fibrosis (OSMF) – a **premalignant condition** characterized by:

- **Progressive fibrosis of the oral mucosa**, leading to **trismus (restricted mouth opening)**.
- **Pale or marble-like appearance of mucosa**, with fibrous bands restricting mobility.
- **Burning sensation (stomatopyrosis), xerostomia, and speech/swallowing difficulties**.

Differential Diagnoses:

- **Ankyloglossia (tongue-tie):** Congenital short lingual frenulum, but lacks submucosal fibrosis and progressive restriction.
- **Scleroderma:** Can cause oral fibrosis but is associated with **systemic skin tightening**.
- **Radiation-induced fibrosis:** In post-radiotherapy patients.
- **Systemic sclerosis:** Features **Raynaud's phenomenon, esophageal dysmotility, and skin thickening**.

Key Next Steps:

1. **Detailed history:**
    - **Areca nut/betel quid/tobacco use?**
    - **Dietary deficiencies (iron, B12)?**
    - **Progressive difficulty opening the mouth?**
2. **Clinical assessment:**
    - Measure **interincisal mouth opening** (if <35 mm, advanced stage).
    - Check for **palpable fibrous bands and mucosal stiffness**.
3. **Biopsy (if needed):**
    - **Dense collagen deposition with atrophic epithelium** confirms OSMF.
4. **Assess for malignant transformation:** High risk (~7-13%) for **oral squamous cell carcinoma (OSCC)**.

Management:

- **Early-stage: Cessation of areca nut/tobacco**, antioxidants (lycopene, curcumin), and intralesional corticosteroids.

- **Advanced cases: Fibrotomy, myotomy, or laser excision** for severe trismus.
- **Regular monitoring: Lifelong surveillance** for malignant transformation.

CASE 19

Reference:
https://pubs.sciepub.com/ijdsr/2/5/1/image/fig1.png

Chat-GPT4 answer:

The image shows a **large ulcerated and irregular lesion on the hard palate**, with **erythema, necrotic tissue, and an infiltrative appearance**. There are also **poor oral hygiene and dental decay** visible in the adjacent teeth.

Most Probable Diagnosis:

This presentation is highly suggestive of **palatal squamous cell carcinoma (OSCC)** or **malignant neoplasm of the maxillary gingiva or palatal mucosa**. The **necrotic appearance, irregular borders, and surrounding inflammation** raise suspicion for **invasive malignancy**.

Differential Diagnoses:

- **Necrotizing sialometaplasia (NS):** Benign **self-limiting ischemic necrosis** of the minor salivary glands, mimicking malignancy but typically resolving in 6-10 weeks.
- **Deep fungal infection (mucormycosis, histoplasmosis):** If the patient is **immunocompromised (diabetes, post-COVID, transplant)**, consider **mucormycosis**, especially if there is rapid tissue destruction.
- **Tuberculosis or tertiary syphilis:** Chronic granulomatous infections that can **cause palatal perforation**.
- **Wegener's granulomatosis (granulomatosis with polyangiitis):** Can cause **midline destruction**, but systemic symptoms (e.g., pulmonary and renal involvement) are expected.

Key Next Steps:

1. **Urgent incisional biopsy** → Histopathology for SCC or deep fungal infection.
2. **Imaging (CT/MRI with contrast):** Assess **tumor invasion** into the **maxilla, sinuses, or orbit**.
3. **Chest X-ray/CT:** To rule out metastasis or **granulomatous disease (TB, sarcoidosis)**.
4. **Microbiological/fungal culture and PAS/GMS staining (if fungal infection suspected).**
5. **HPV testing (if OSCC is confirmed),** as HPV-related tumors may have a better prognosis.

Management Approach (if SCC is confirmed):

- **Stage-dependent treatment:**
  - **Early-stage (T1-T2, N0):** Surgical excision ± neck dissection.
  - **Locally advanced (T3-T4, N+):** Surgery + adjuvant **radiotherapy ± chemotherapy**.
  - **Unresectable cases:** Consider **immunotherapy (PD-1 inhibitors like pembrolizumab or nivolumab)**.

Prognosis:

- **OSCC of the palate often presents late**, leading to **invasion into deep structures (maxillary sinus, nasal cavity)**.

- **Early diagnosis and aggressive treatment** improve survival.

CASE 20

Reference:

https://specialistidelsorriso.it/wp-content/uploads/2023/07/afte-in-bocca-una-dolorosa-sintomatologia-da-trattare-cosi.jpg

Chat-GPT4 answer:

The image shows **multiple well-defined, round, shallow ulcerations with a white pseudomembranous center and an erythematous halo** on the **ventral surface of the tongue and inner lower lip**.

Most Probable Diagnosis:

The lesions are highly suggestive of **recurrent aphthous stomatitis (RAS)**, also known as **canker sores**. These are **painful, non-contagious ulcers** that typically heal within **10-14 days** without scarring.

Differential Diagnoses:

- **Herpetic stomatitis (HSV-1 infection):** Typically presents with **clustered vesicles that rupture into ulcers**, often involving the **keratinized mucosa (gingiva, hard palate, lips)**.
- **Oral lichen planus (erosive form):** Chronic ulcers with a **reticular pattern (Wickham's striae)**.
- **Traumatic ulcer:** May occur due to **mechanical injury (e.g., biting, sharp teeth, braces)** but usually **solitary** and heals quickly.
- **Behçet's disease:** If associated with **genital ulcers, ocular inflammation, or systemic symptoms**.
- **Autoimmune conditions (pemphigus vulgaris, pemphigoid, lupus erythematosus):** If ulcers are chronic, widespread, or accompanied by **skin involvement**.
- **Nutritional deficiencies (iron, folate, vitamin B12):** If recurrent or associated with **glossitis and angular cheilitis**.

Key Next Steps:

1. **Assess history:**
    - **Recurrent ulcers?** (suggests RAS)
    - **Triggers?** (stress, trauma, acidic foods)
    - **Systemic symptoms?** (rule out Behçet's or autoimmune disease)
2. **Topical treatment:**
    - **Corticosteroids (e.g., clobetasol, fluocinonide, or dexamethasone rinse)**
    - **Lidocaine gel** for pain relief
    - **Chlorhexidine mouthwash** for secondary infection prevention
3. **Systemic treatment (if severe or frequent):**
    - **Colchicine, dapsone, or thalidomide** in refractory cases.

4. **Nutritional assessment:** Consider **iron, folate, B12 supplementation** if deficiencies are suspected.

When to Biopsy?

- If ulcers are **persistent (>3 weeks)**.
- If they appear **atypical (indurated, proliferative, or non-healing)**.
- If there is suspicion of **immune-mediated or neoplastic conditions**.

https://www.researchgate.net/publication/233942366/figure/fig1/AS:931957898760194@1599207486328/A-typical-squamous-cell-carcinoma-of-the-floor-of-the-mouth.png

The image shows a **large, irregular, ulceroproliferative lesion on the ventral and lateral tongue**, with **erythematous and keratotic areas, induration, and surface ulceration**.

Most Probable Diagnosis:

The lesion is highly suggestive of **oral squamous cell carcinoma (OSCC)**. The **lateral border of the tongue** is a common site for **malignant transformation**, especially in individuals with **risk factors** such as **tobacco, alcohol use, betel quid chewing, or HPV exposure**.

Differential Diagnoses:

- **Oral leukoplakia with dysplasia:** If the lesion started as a white patch, it could have undergone malignant transformation.
- **Traumatic ulcer/granuloma:** If there is a history of chronic irritation (e.g., sharp tooth, dentures), but these lesions typically **heal within 2 weeks** after removing the cause.
- **Erosive lichen planus:** Usually has **bilateral reticular striations (Wickham's striae)** and is more widespread.
- **Chronic hyperplastic candidiasis:** White, non-scrapable lesion, but lacks the ulcerated and infiltrative nature seen here.
- **Syphilitic ulcer (tertiary syphilis):** Could mimic OSCC but is associated with **systemic findings**.

Key Next Steps:

1. **Urgent incisional biopsy:** To confirm OSCC and assess histopathological grade.
2. **Imaging (CT/MRI with contrast):** To evaluate **tumor invasion and lymph node involvement**.
3. **HPV testing (if suspected):** HPV-positive OSCCs (especially p16-positive) have a **better prognosis**.
4. **TNM Staging:** Based on **tumor size, lymph node involvement, and metastasis**.
5. **Multidisciplinary team approach:** Head and neck oncology, maxillofacial surgery, radiation oncology.

Management Approach (if SCC is confirmed):

- **Early-stage (T1-T2, N0): Surgical excision** with negative margins.
- **Locally advanced (T3-T4, N+): Surgery + adjuvant radiotherapy ± chemotherapy**.

- **Unresectable/metastatic cases:** Consider **immunotherapy (PD-1 inhibitors like pembrolizumab or nivolumab).**

Prognosis:

- **Early detection and treatment** significantly improve **5-year survival rates**.
- **Regular follow-up** is necessary due to **risk of recurrence and second primary tumors**.

CASE 21

Reference:

https://www.researchgate.net/publication/225058197/figure/fig2/AS:646431320854528@1531132645129/Left-retromolar-squamous-cell-carcinoma-case-3.png

Chat-GPT4 answer:

The image shows a **large ulcerative lesion on the posterior buccal mucosa**, characterized by **irregular borders, erythema, and a central necrotic or keratotic area**.

Most Probable Diagnosis:

This lesion is highly **suspicious for oral squamous cell carcinoma (OSCC)**, given its **ulcerative nature, irregular margins, and indurated appearance**. OSCC is the most common **malignant neoplasm of the oral cavity** and often arises in **high-risk sites**, including the **buccal mucosa, tongue, and gingiva**.

Differential Diagnoses:

- **Traumatic ulcer (TUGSE - Traumatic Ulcerative Granuloma with Stromal Eosinophilia):** If there is a history of chronic irritation (sharp teeth, dentures), but these lesions **heal within 2-3 weeks** once the cause is removed.
- **Oral leukoplakia with dysplasia:** If the lesion started as a **white patch**, it could have undergone **malignant transformation**.
- **Necrotizing sialometaplasia:** A **benign ischemic process** that mimics malignancy but typically affects the **palate**.
- **Chronic hyperplastic candidiasis:** Can present as a **white, non-scrapable lesion**, but typically lacks ulceration and induration.
- **Syphilitic ulcer (tertiary syphilis - gumma):** Could mimic malignancy but is associated with **systemic findings (e.g., neurologic symptoms, cardiovascular involvement).**
- **Tuberculosis (TB) ulcer:** Chronic **painless** ulcers with **caseating necrosis**, often in immunocompromised individuals.

Key Next Steps:

1. **Urgent incisional biopsy:** To confirm **OSCC** and assess histopathological grade.
2. **Imaging (CT/MRI with contrast):** To evaluate **tumor invasion and lymph node involvement**.
3. **HPV testing (if suspected):** HPV-positive OSCCs (especially **p16-positive**) have a **better prognosis**.
4. **TNM Staging:** Based on **tumor size, lymph node involvement, and metastasis**.
5. **Multidisciplinary team approach: Head and neck oncology, maxillofacial surgery, radiation oncology**.

Management Approach (if SCC is confirmed):

- **Early-stage (T1-T2, N0): Surgical excision** with negative margins.
- **Locally advanced (T3-T4, N+): Surgery + adjuvant radiotherapy ± chemotherapy**.

- **Unresectable/metastatic cases:** Consider **immunotherapy (PD-1 inhibitors like pembrolizumab or nivolumab).**

Prognosis:

- **Early detection and treatment significantly improve 5-year survival rates**.
- **Regular follow-up** is necessary due to **high recurrence r**

CASE 22

Reference:

https://www.researchgate.net/publication/51854767/figure/fig2/AS:195404902735873@1423599567522/Female-44-year-old-patient-with-oral-melanoma-in-tongue-with-a-2-year-disease-length.png

Chat-GPT4 answer:

The image shows a **large, necrotic, and ulcerated mass on the lateral tongue**, with **areas of black necrosis and fibrinous exudate**. The lesion appears **exophytic, irregular, and infiltrative**, with surrounding erythema.

Most Probable Diagnosis:

The most concerning and likely diagnosis is **advanced oral squamous cell carcinoma (OSCC) with necrosis**. The presence of **black necrotic tissue (indicative of ischemia or tumor necrosis)**, an **exophytic and ulcerative growth**, and its location on the **lateral border of the tongue (a high-risk site)** strongly suggests **invasive carcinoma**.

Differential Diagnoses:

- **Mucormycosis (rhinocerebral or orofacial involvement):** If the patient is **immunocompromised (diabetes, post-COVID, transplant)**, deep fungal infection could present as **black necrotic tissue** due to **angioinvasion**.
- **Necrotizing sialometaplasia:** A **self-limiting ischemic process** but typically affects the **palate**, not the tongue.
- **Malignant melanoma (oral variant):** If pigmentation is observed, but oral melanoma is **rare** and typically appears **nodular with diffuse pigmentation**.
- **Tuberculous ulcer or syphilitic gumma:** Rare but can cause **chronic ulceration** with necrosis.

Key Next Steps:

1. **Urgent incisional biopsy** to confirm malignancy and assess tumor grade.
2. **CT/MRI with contrast** to evaluate **tumor invasion and nodal involvement**.
3. **Histopathological analysis** to differentiate between **OSCC, deep fungal infection, or another malignancy**.
4. **Fungal culture and PAS/GMS staining** if mucormycosis is suspected.
5. **Multidisciplinary approach** involving **head and neck oncology, maxillofacial surgery, and radiation oncology**.

Management Approach (if SCC is confirmed):

- **Early-stage (T1-T2, N0): Surgical excision** with negative margins ± selective neck dissection.
- **Locally advanced (T3-T4, N+): Surgery + adjuvant radiotherapy ± chemotherapy**.
- **Unresectable/metastatic cases:** Consider **immunotherapy (PD-1 inhibitors like pembrolizumab)**.

Prognosis:

- **Advanced OSCC has a high risk of metastasis (lymphatic spread) and poor prognosis** if not treated early.
- **Early intervention and aggressive treatment improve survival rates**.

CASE 23

Reference:
https://opmdcare.com/wp-content/uploads/oral-erythroplakia-image2.jpeg

Chat-GPT4 answer:

The image shows **erythematous, inflamed mucosa on the buccal mucosa**, with areas of **erosion and irregular redness**. The lesion appears **diffuse**, with no clear ulceration or induration.

Most Probable Diagnosis:

This presentation is highly suggestive of **erosive oral lichen planus (OLP)**, an **immune-mediated mucosal disorder** that presents as **erythematous, atrophic, or ulcerated areas**, often accompanied by **reticular white striations (Wickham's striae)**.

Differential Diagnoses:

- **Candidiasis (atrophic or erythematous type):** Diffuse red mucosal involvement, often associated with **burning sensation**, but typically responds to **antifungal therapy**.
- **Traumatic or irritative mucositis:** Due to chronic trauma (e.g., dentures, sharp restorations), but should resolve once the irritant is removed.
- **Allergic contact stomatitis:** Can result from **dental materials (e.g., amalgam, composites), toothpaste, or mouthwashes**.
- **Erythroplakia:** A high-risk **premalignant lesion** that should be **biopsied if persistent**.
- **Pemphigus vulgaris or mucous membrane pemphigoid:** Autoimmune conditions that present with **mucosal erosions, desquamation, and blistering**.

Key Next Steps:

1. **Clinical correlation:**
    - **Any systemic conditions?** (e.g., autoimmune diseases)
    - **Symptoms of burning sensation or pain?**
    - **Presence of white reticulations (Wickham's striae)?**
2. **Histopathological confirmation (biopsy):**
    - If OLP is suspected, look for **band-like lymphocytic infiltrate, basal cell degeneration**.
    - If concern for dysplasia or malignancy, biopsy should rule out **erythroplakia or early SCC**.
3. **Topical treatment (if symptomatic):**
    - **Topical corticosteroids (clobetasol propionate 0.05%)** as first-line therapy.
    - **Calcineurin inhibitors (e.g., tacrolimus) for refractory cases**.
4. **Regular follow-up:** OLP has a **low risk (~1-2%) of malignant transformation** over time.

When to Biopsy?

- If lesions **persist for more than 2 weeks**.
- If there is **induration, ulceration, or resistance to treatment**.
- If lesions appear **atypical (nodular, proliferative, or rapidly expanding).**

CASE 24

Reference:
https://www.dentaljournal.it/wp-content/uploads/2018/09/iperplasia-gengivale.png

Chat-GPT4 answer:

The image shows **generalized gingival overgrowth**, characterized by **firm, lobulated, and enlarged gingival tissue covering the crowns of the teeth**.

Most Probable Diagnosis:

This presentation is highly suggestive of **gingival hyperplasia (gingival overgrowth)**, which can be associated with **various etiologies**.

Possible Causes of Gingival Overgrowth:

1. **Drug-induced gingival hyperplasia (DIGH):**
    - **Common drugs:**
        - **Calcium channel blockers** (e.g., nifedipine, amlodipine)
        - **Anticonvulsants** (e.g., phenytoin)
        - **Immunosuppressants** (e.g., cyclosporine)
    - **Features:** Firm, fibrotic gingival enlargement, more pronounced in the **anterior region**.
2. **Hereditary gingival fibromatosis (HGF):**
    - **Rare, genetic disorder** causing **slow-growing, fibrotic gingival enlargement**.
    - Typically starts in **childhood or adolescence**.
3. **Leukemic gingival enlargement:**
    - Occurs in **acute myeloid leukemia (AML)** or **chronic myeloid leukemia (CML)**.
    - **Features:** Soft, **erythematous, and edematous** gingiva, often with **spontaneous bleeding**.
4. **Inflammatory gingival enlargement (chronic periodontitis-associated):**
    - Caused by **poor oral hygiene, plaque accumulation, and periodontal disease**.
    - Tissues appear **soft, edematous, and red**.
5. **Hormonal gingival hyperplasia:**
    - **Pregnancy, puberty, or oral contraceptive use** can exacerbate inflammation-related gingival overgrowth.

Key Next Steps:

1. **Detailed medical history:**
    - **Medications?**
    - **Genetic history?**
    - **Symptoms of leukemia (fatigue, bleeding, infections)?**
2. **Clinical examination:** Assess **consistency, extent, and bleeding tendency**.
3. **Blood tests (if leukemia is suspected): Complete blood count (CBC) with differential**.
4. **Biopsy (if needed):** To rule out **neoplastic or hereditary conditions**.
5. **Treatment:**
    - **Drug substitution (if drug-induced):** Consult with the prescribing physician.

- **Professional scaling and root planing (if inflammatory).**
- **Gingivectomy or laser excision (for severe cases).**
- **Strict oral hygiene with chlorhexidine rinses**.

CASE 25

Reference:
https://static.medicitalia.it/public/uploadedfiles/img-lic/staff_lingua-carta-geografica-lesioni-circolari.jpg

Chat-GPT4 answer:

The image shows **white, irregular, swirling or curvilinear patterns on the dorsal surface of the tongue**, with areas of **erythema and depapillation**.

Most Probable Diagnosis:

This presentation is highly suggestive of **benign migratory glossitis (BMG), also known as geographic tongue**. It is a **benign, inflammatory condition** characterized by:

- **Erythematous areas of depapillation** due to loss of filiform papillae.
- **White or yellowish raised borders** forming irregular patterns.
- **Lesions that change shape and migrate over time**.

Differential Diagnoses:

- **Oral lichen planus (reticular form):** Typically presents with **Wickham's striae (white lacy reticulations)**, but does not **migrate**.
- **Candidiasis (atrophic or pseudomembranous):** Usually involves **white plaques that can be wiped off**, often with associated burning.
- **Leukoplakia:** Persistent white patches that do not rub off and may be **premalignant**.
- **Nutritional deficiencies (B12, iron, folate):** Can cause **atrophic glossitis** but lacks the characteristic migrating lesions.

Key Next Steps:

1. **Clinical history:**
    - Symptoms? (burning, irritation?)
    - History of atopic conditions (as BMG is associated with allergies, asthma).
2. **Examination over time:** If the lesion **migrates**, it confirms BMG.
3. **Management:**
    - **Reassurance** (BMG is benign and self-limiting).
    - If symptomatic, consider **topical corticosteroids or antihistamines**.
    - Avoid irritants (spicy, acidic foods, alcohol).

Prognosis:

- **Chronic but benign**, with periodic flare-ups.
- No risk of **malignant transformation**.

CASE 26

Reference:
https://ars.els-cdn.com/content/image/1-s2.0-S1166341319301071-gr3.jpg

Chat-GPT4 answer:

The image shows a **thick white to yellowish coating on the dorsal surface of the tongue**, extending from the posterior to the midsection, with an uneven distribution.

Most Probable Diagnosis:

This presentation is most consistent with **oral candidiasis (pseudomembranous type, also known as thrush)**, caused by an **overgrowth of Candida species**, commonly **Candida albicans**.

Differential Diagnoses:

- **Hairy tongue:** Characterized by **elongated filiform papillae with a stained, hairy-like appearance** rather than a uniform coating.
- **Leukoplakia:** Persistent **white plaques that do not wipe off**, often premalignant.
- **Lichen planus (keratotic form):** Typically presents with **reticular white striations (Wickham's striae)** rather than diffuse coating.
- **Xerostomia-related tongue coating:** Seen in patients with **dry mouth (Sjogren's syndrome, medication-induced xerostomia)**.
- **Poor oral hygiene-related tongue coating:** Can be caused by **bacterial overgrowth and food debris**.

Key Next Steps:

1. **Clinical history:**
   - **Any risk factors?** (immunosuppression, diabetes, antibiotic/steroid use, HIV, dry mouth)
   - **Symptoms?** (burning sensation, altered taste, difficulty swallowing)
2. **Diagnostic test:**
   - **KOH preparation or fungal culture** (for confirmation).
3. **Treatment:**
   - **Topical antifungals: Nystatin (oral rinse) or clotrimazole troches**.
   - **Systemic antifungals (if severe/refractory): Fluconazole 100 mg daily for 7-14 days**.
   - **Improving oral hygiene** and considering **probiotic supplementation**.

Prognosis:

- **Resolves with appropriate antifungal therapy**.
- **Recurrent cases require investigation for underlying immunosuppression (diabetes, HIV, chemotherapy effects).**

CASE 27

Reference:

https://marianiumberto.it/wp-content/uploads/2021/09/DSC_6864mod.jpg

Chat-GPT4 answer:

The image shows **generalized gingival overgrowth**, particularly affecting the **anterior maxillary region**, with **smooth, pale pink to erythematous swelling**. The enlargement appears **fibrotic and firm**, without signs of acute inflammation or ulceration.

Most Probable Diagnosis:

The findings suggest **gingival hyperplasia (gingival overgrowth)**, which may be caused by **various underlying conditions**.

Possible Causes of Gingival Overgrowth:

1. **Drug-induced gingival hyperplasia (DIGH):**
    - **Common drugs:**
        - **Calcium channel blockers** (e.g., nifedipine, amlodipine)
        - **Anticonvulsants** (e.g., phenytoin)
        - **Immunosuppressants** (e.g., cyclosporine)
    - **Features:** Firm, **fibrotic, and lobulated gingival overgrowth**, especially in the **anterior region**.
2. **Hereditary gingival fibromatosis (HGF):**
    - A **rare genetic disorder** causing **slow-growing, non-inflammatory fibrotic gingival overgrowth**.
    - Typically starts in **childhood or adolescence**.
3. **Leukemic gingival enlargement:**
    - Occurs in **acute myeloid leukemia (AML)** or **chronic myeloid leukemia (CML)**.
    - **Features:** Soft, **erythematous, and edematous gingiva**, often with **spontaneous bleeding and systemic symptoms (pallor, fatigue, fever, bruising).**
4. **Inflammatory gingival enlargement (chronic periodontitis-associated):**
    - Caused by **poor oral hygiene, plaque accumulation, and periodontal disease**.
    - Tissues appear **soft, edematous, and reddish**.
5. **Hormonal gingival hyperplasia:**
    - **Pregnancy, puberty, or oral contraceptive use** can exacerbate inflammation-related gingival overgrowth.

Key Next Steps:

1. **Medical history assessment:**
    - **Medication use?**
    - **Family history of gingival overgrowth?**
    - **Symptoms of leukemia (fatigue, bleeding, recurrent infections)?**

2. **Clinical examination:**
    - Assess **texture, consistency, and extent of overgrowth**.
    - Check for **plaque accumulation or calculus deposits**.
3. **Blood tests (if leukemia suspected):**
    - **Complete blood count (CBC) with differential**.
4. **Biopsy (if needed):**
    - To rule out **neoplastic, hereditary, or systemic conditions**.
5. **Treatment:**
    - **Drug substitution (if drug-induced):** Consult with the prescribing physician.
    - **Professional scaling and root planing (if inflammatory cause).**
    - **Gingivectomy or laser excision** (for severe cases).
    - **Strict oral hygiene with chlorhexidine mouth rinses**.

Prognosis:

- **Drug-induced and inflammatory cases improve with treatment.**
- **Hereditary forms may require periodic surgical management.**
- **Leukemic gingival enlargement requires urgent systemic treatment.**

Would you like to discuss **treatment protocols, surgical management, or prevention strategies for gingival hyperplasia?**

CASE 28

Reference:
https://upload.wikimedia.org/wikipedia/commons/thumb/3/34/Torus_cropped.jpg/640px-Torus_cropped.jpg

Chat-GPT4 answer:

The image shows a **well-circumscribed, firm, sessile, and lobulated swelling on the gingiva**, appearing pink to reddish in color. The lesion is adjacent to teeth, with no clear signs of ulceration or necrosis.

Most Probable Diagnosis:

This lesion is highly suggestive of a **pyogenic granuloma (PG)**, a **benign vascular lesion** often associated with **chronic irritation, hormonal changes, or poor oral hygiene**.

Differential Diagnoses:

1. **Peripheral ossifying fibroma (POF):**
   - Similar appearance but **firmer, more fibrotic**, and contains **calcifications** on radiographs.
2. **Peripheral giant cell granuloma (PGCG):**
   - Bluish or purple in color, often associated with **bony resorption**.
3. **Fibroma:**
   - **More firm and pale**, does not exhibit **vascular proliferation**.
4. **Gingival hyperplasia:**
   - More **diffuse and generalized**, often due to **medications** (e.g., calcium channel blockers, phenytoin, cyclosporine).
5. **Malignant lesions (rare, but to rule out):**
   - Squamous cell carcinoma or **spindle cell tumors** (if ulcerated, rapidly growing, or recurrent).

Key Next Steps:

1. **Clinical history:**
   - Any history of **trauma, chronic irritation (dental appliances, restorations), pregnancy, or hormonal changes?**
2. **Radiographic evaluation:**
   - To rule out **bony involvement** (especially for POF or PGCG).
3. **Biopsy (if atypical or persistent):**
   - Histopathology confirms **vascular proliferation, inflammatory cells, and granulation tissue**.
4. **Management:**
   - **Surgical excision** (complete removal, including any irritants).
   - **Scaling and root planing** to prevent recurrence.
   - **Hormonal evaluation** if occurring during pregnancy (pregnancy tumor).
   - **Recurrence risk:** Moderate, especially if **irritants are not eliminated**.

Prognosis:

- **Benign and non-malignant**.
- Recurrence possible if the **source of irritation is not removed**.

Would you like to discuss **biopsy techniques, surgical excision, or recurrence prevention strategies?**

CASE 29

Reference:

https://medicinaonline.co/wp-content/uploads/2017/03/medicina-online-lingua-nera-villosa-cause-diagnosi-terapie-black-tongue.jpg

Chat-GPT4 answer:

The image shows **blackish-brown discoloration and elongated filiform papillae on the dorsal surface of the tongue**, with a characteristic **"hairy" appearance**.

Most Probable Diagnosis:

This presentation is highly suggestive of **black hairy tongue (lingua villosa nigra)**, a benign condition caused by **excessive keratinization and delayed desquamation of the filiform papillae**, leading to accumulation of **bacteria, fungi, food debris, and staining agents**.

Risk Factors for Black Hairy Tongue:

- **Poor oral hygiene**
- **Smoking**
- **Excessive coffee, tea, or alcohol consumption**
- **Broad-spectrum antibiotic use (e.g., tetracyclines)**
- **Xerostomia (dry mouth)**
- **Radiation therapy to the head and neck**
- **Use of mouthwashes containing oxidizing agents (e.g., hydrogen peroxide, chlorhexidine)**

Differential Diagnoses:

- **Oral melanotic macules:** Typically **flat** and non-hairy.
- **Pseudomembranous candidiasis:** White patches rather than **black discoloration**.
- **Oral hairy leukoplakia (OHL):** Associated with **HIV/AIDS, caused by Epstein-Barr virus**, affecting the lateral tongue, not the dorsum.

Key Next Steps:

1. **Clinical history:** Assess for **risk factors (smoking, medication use, oral hygiene habits)**.
2. **Tongue scraping:** Removing the keratinized layer should improve symptoms.
3. **Management:**
   - **Improving oral hygiene** (regular tongue brushing with a soft toothbrush).
   - **Cessation of smoking and alcohol**.
   - **Increased hydration and dietary modifications**.
   - **Discontinuation of causative agents (e.g., antibiotics, oxidizing mouthwashes)**.
   - **Antifungal therapy** only if secondary candidiasis is present.

Prognosis:

- **Completely benign and reversible** with improved oral hygiene.
- **Recurrence is common** if risk factors persist.

Would you like to discuss **specific oral hygiene recommendations, risk factor management, or differential diagnoses in more detail?**

https://figures.semanticscholar.org/d2a4adfa2a031dc8514c99606ca55a103335175d/2-Figure1-1.png

The image shows a **large, irregular, exophytic, and proliferative lesion on the hard palate**, with a **verrucous (wart-like) surface and yellowish keratotic projections**. Surrounding tissue appears **erythematous**, and there is **poor oral hygiene with dental deposits**.

Most Probable Diagnosis:

This lesion is highly suspicious for **verrucous carcinoma (VC)**, a **low-grade variant of oral squamous cell carcinoma (OSCC)**. It is a **slow-growing, well-differentiated, exophytic tumor** that commonly affects the **gingiva, buccal mucosa, and hard palate**, particularly in **smokers, areca nut chewers, and immunocompromised patients**.

Differential Diagnoses:

1. **Proliferative verrucous leukoplakia (PVL):**
   - **Multiple verrucous white plaques** that **progress** over time.
   - High potential for **malignant transformation** into SCC.
2. **Papillomatosis (HPV-associated verrucous lesions):**
   - **Smaller, multiple lesions** rather than a single large growth.
   - **Caused by HPV (subtypes 6, 11, 16, 18, 31, 33).**
3. **Necrotizing sialometaplasia:**
   - **Benign, self-limiting**, presents with **ulceration rather than exophytic growth**.
4. **Chronic hyperplastic candidiasis:**
   - **White plaques that do not wipe off**, but lacks the **verrucous, thickened nature** of this lesion.
5. **Giant cell granuloma or peripheral ossifying fibroma:**
   - Generally occurs on **gingiva**, not the hard palate.

Key Next Steps:

1. **Urgent incisional biopsy:**
   - Histopathology will confirm **verrucous carcinoma (VC)** (papillary hyperplasia with well-differentiated squamous cells and minimal atypia).
2. **HPV testing:**
   - If suspected, **p16 immunohistochemistry or PCR for HPV subtypes**.
3. **Imaging (CT/MRI):**
   - To assess **tumor depth, bony invasion, and lymph node involvement**.
4. **Treatment approach (if VC is confirmed):**
   - **Wide surgical excision (first-line treatment).**

- **NO radiation as first-line therapy**, as VC can undergo **anaplastic transformation into aggressive SCC**.
- **Rehabilitation and prosthodontic management if maxillary bone is involved.**

Prognosis:

- **Better prognosis than conventional OSCC**, as VC is **slow-growing and less likely to metastasize**.
- **High recurrence risk if not completely excised.**

Would you like to discuss **biopsy techniques, HPV involvement, or treatment planning in more detail?**

CASE 30

Reference:
https://storage.googleapis.com/dl.dentistrykey.com/clinical/OralCavity/1_3s20B9780323415187000080.jpg

Chat-GPT4 answer:

The image shows a **large, ulcerative, and exophytic lesion on the floor of the mouth**, with **irregular borders, surface erosion, and areas of necrosis**. The lesion appears **indurated and infiltrative**, with surrounding erythema.

Most Probable Diagnosis:

This lesion is highly **suspicious for oral squamous cell carcinoma (OSCC)**, given its **location (floor of the mouth), ulcerative nature, and infiltrative characteristics**. The **floor of the mouth** is a high-risk site for OSCC, especially in individuals with **tobacco, alcohol, or betel quid use**.

Differential Diagnoses:

- **Chronic traumatic ulcer:** If there is a history of **mechanical trauma (sharp tooth, ill-fitting dentures)**, but these lesions usually **heal within 2-3 weeks** after removing the cause.
- **Oral leukoplakia with dysplasia:** A white plaque with potential **malignant transformation**.
- **Necrotizing sialometaplasia:** Benign, **self-limiting ischemic necrosis**, but more common on the **palate**.
- **Tuberculosis ulcer:** Chronic ulceration with **caseous necrosis**, often in **immunocompromised patients**.
- **Syphilitic ulcer (tertiary syphilis - gumma):** Can mimic OSCC but is associated with **systemic symptoms (neurologic, cardiovascular involvement).**
- **Deep fungal infections (histoplasmosis, blastomycosis, mucormycosis):** More common in **immunocompromised individuals**.

Key Next Steps:

1. **Urgent incisional biopsy:** To confirm **OSCC and assess histopathological grade**.
2. **Imaging (CT/MRI with contrast):** To evaluate **tumor invasion and lymph node involvement**.
3. **HPV testing (if suspected):** HPV-positive OSCCs (especially **p16-positive**) have a **better prognosis**.
4. **TNM Staging:** Based on **tumor size, lymph node involvement, and metastasis**.
5. **Multidisciplinary team approach: Head and neck oncology, maxillofacial surgery, radiation oncology**.

Management Approach (if SCC is confirmed):

- **Early-stage (T1-T2, N0): Surgical excision** with negative margins.
- **Locally advanced (T3-T4, N+): Surgery + adjuvant radiotherapy ± chemotherapy**.
- **Unresectable/metastatic cases:** Consider **immunotherapy (PD-1 inhibitors like pembrolizumab or nivolumab).**

Prognosis:

- **Early detection and treatment significantly improve 5-year survival rates**.
- **Regular follow-up** is necessary due to **risk of recurrence and second primary tumors**.

Would you like to discuss **biopsy techniques, OSCC staging, or treatment planning?**

CASE 31

Reference:
https://media-us.amboss.com/media/thumbs/big_5081d952cf2c7.jpg

Chat-GPT4 answer:

The image shows a **large, ulceroproliferative lesion on the floor of the mouth**, with **irregular borders, surface erosion, necrosis, and induration**. The lesion appears **infiltrative and exophytic**, with surrounding **erythema and poor oral hygiene**.

Most Probable Diagnosis:

This lesion is highly **suspicious for oral squamous cell carcinoma (OSCC)**, considering:

- **Ulcerative and exophytic features**.
- **Irregular, indurated, and infiltrative appearance**.
- **Common high-risk location (floor of the mouth)**.
- **Associated poor oral hygiene and possible risk factors (tobacco, alcohol, betel quid, HPV exposure).**

Differential Diagnoses:

- **Chronic traumatic ulcer:** Typically heals within **2-3 weeks** after removing the cause (e.g., sharp tooth, denture trauma).
- **Necrotizing sialometaplasia:** Usually self-limiting and found on the **palate** rather than the floor of the mouth.
- **Chronic hyperplastic candidiasis:** White plaques with **fungal overgrowth**, but lacks **infiltration and ulceration**.
- **Tuberculosis ulcer:** Chronic, **painless** ulcers with **caseous necrosis**, seen in immunocompromised patients.
- **Tertiary syphilitic ulcer (gumma):** Can mimic malignancy but is associated with **systemic symptoms**.

Key Next Steps (Urgent):

1. **Incisional biopsy:** To confirm OSCC and assess histological grade.
2. **Imaging (CT/MRI with contrast):** Evaluate **tumor invasion and lymph node involvement**.
3. **HPV testing (p16 immunohistochemistry or PCR):** HPV-positive OSCCs **(especially p16-positive)** have a **better prognosis**.
4. **TNM Staging:** Based on **tumor size (T), nodal involvement (N), and distant metastasis (M)**.
5. **Multidisciplinary team consultation:** Involves **head and neck oncology, maxillofacial surgery, and radiation oncology**.

Management Approach (if SCC is confirmed):

- **Early-stage (T1-T2, N0): Surgical excision** with negative margins.
- **Locally advanced (T3-T4, N+): Surgery + adjuvant radiotherapy ± chemotherapy**.

- **Unresectable/metastatic cases:** Consider **immunotherapy (PD-1 inhibitors like pembrolizumab or nivolumab).**

Prognosis:

- **Early detection improves survival (5-year survival: 70-90% for early-stage, 30-50% for advanced cases).**
- **Regular follow-up is essential due to the high risk of recurrence and secondary tumors.**

Would you like to discuss **OSCC staging, biopsy techniques, or treatment planning in more detail?**

CASE 32

Reference:
https://www.pcds.org.uk/imager/gallery/clinical/leukoplakia/12267/cheek-biting-02_fee391183f15cb4d62773032fe0be92d.jpg

Chat-GPT4 answer:

The image shows **a whitish, linear, and slightly elevated lesion on the buccal mucosa**, running along the occlusal plane, with **no ulceration, erythema, or irregular margins**.

Most Probable Diagnosis:

This lesion is characteristic of **Linea Alba**, a **benign, physiological, and frictional keratosis** resulting from **chronic irritation due to cheek biting, sucking, or pressure from teeth**.

Key Features of Linea Alba:

- **Bilateral white line** on the buccal mucosa along the occlusal plane.
- **Asymptomatic, well-demarcated, and non-wipeable**.
- No signs of **inflammation, ulceration, or induration**.
- **More prominent in individuals with parafunctional habits (e.g., cheek biting, bruxism).**

Differential Diagnoses:

1. **Leukoplakia:** Persistent white patches that do not rub off, but usually **not confined to the occlusal line**.
2. **Frictional keratosis:** Similar to linea alba but seen in areas of chronic trauma (e.g., sharp restorations, ill-fitting dentures).
3. **Oral lichen planus (reticular form):** White striations with a lace-like pattern (Wickham's striae), often **bilateral and affecting multiple sites**.
4. **Morsicatio buccarum (chronic cheek chewing):** Presents as **irregular, shredded, or macerated white areas** rather than a smooth line.
5. **Oral candidiasis (hyperplastic type):** White plaques that do not wipe off, but typically associated with **fungal overgrowth and burning sensation**.

Management and Recommendations:

- **No treatment is necessary** as linea alba is **a normal anatomical variation**.
- If **parafunctional habits (cheek biting, bruxism)** are suspected, consider:
  - **Occlusal splint/night guard** if bruxism is present.
  - **Patient education** on avoiding cheek biting.
- **Re-evaluation if lesion changes over time** (e.g., thickens, ulcerates, or becomes symptomatic).

Prognosis:

- **Completely benign and does not require intervention.**
- **No risk of malignant transformation.**

Would you like to discuss **parafunctional habit management or differential diagnoses in more detail?**

https://i0.wp.com/images-prod.healthline.com/hlcmsresource/images/4377-Everything_You_Need_to_Know_About_Tongue_Cancer-642x361-oral_leukoplakia-slide1.jpg?w=1155&h=758

The image shows a **white, non-homogeneous, irregular lesion on the lateral border of the tongue**, with **a slightly raised, rough, and indurated appearance**.

Most Probable Diagnosis:

This lesion is **highly suspicious for leukoplakia**, a **potentially malignant disorder (PMD)** of the oral mucosa. Given its **location (lateral tongue, a high-risk site) and non-homogeneous pattern**, it may represent **dysplastic or early malignant transformation**.

Differential Diagnoses:

1. **Oral lichen planus (reticular form)**
   - Presents as **bilateral, lace-like white striations (Wickham's striae)**.
   - Typically affects **buccal mucosa, tongue, and gingiva**.
2. **Oral hairy leukoplakia (OHL):**
   - **White, corrugated plaques on the lateral tongue**.
   - Associated with **Epstein-Barr virus (EBV) and HIV/AIDS**.
   - Does **not rub off** and is often **bilateral**.
3. **Chronic hyperplastic candidiasis:**
   - **White, non-scrapable plaques**, often associated with **fungal overgrowth**.
   - Responds to **antifungal therapy**.
4. **Frictional keratosis (morsicatio linguarum):**
   - Due to **chronic mechanical irritation** (sharp teeth, cheek biting).
   - **Reversible** after eliminating the source of trauma.
5. **Oral squamous cell carcinoma (early stage):**
   - Leukoplakic lesions with **induration, ulceration, or nodularity** should raise suspicion.
   - Requires **urgent biopsy**.

Key Next Steps:

1. **Biopsy (gold standard):**
   - **Incisional biopsy** to assess dysplasia or malignancy.
   - **Histopathology determines degree of dysplasia (mild, moderate, severe).**
2. **Elimination of risk factors:**
   - **Smoking, alcohol, betel quid, HPV infection.**
   - Regular follow-up for any **persistent or changing lesions**.
3. **Management:**
   - **If no dysplasia:** Conservative monitoring with lifestyle modification.
   - **Mild dysplasia:** Observation with follow-up.
   - **Moderate/severe dysplasia:** Consider **excision or laser ablation**.

    - **Malignant transformation:** Requires **oncologic evaluation and definitive treatment**.

Prognosis:

- **Leukoplakia has a 5-17% malignant transformation rate** (higher in **non-homogeneous, high-risk sites** like the lateral tongue).
- **Early detection and biopsy are critical for preventing malignant progression.**

Would you like to discuss **biopsy techniques, leukoplakia grading, or malignant transformation risk factors?**

CASE 33

Reference:

https://opmdcare.com/wp-content/uploads/leukoplakia-image3-1024x683.jpeg

Chat-GPT4 answer:

The image shows a **white, irregular, non-homogeneous patch on the buccal mucosa**, with a slightly raised and keratotic surface. The lesion does not appear ulcerated or inflamed.

Most Probable Diagnosis:

This lesion is highly suggestive of **oral leukoplakia**, a **potentially malignant disorder (PMD)** of the oral mucosa, especially considering:

- **White, non-wipeable plaque.**
- **Irregular, slightly thickened, non-homogeneous surface.**
- **Buccal mucosa involvement** (a common site for leukoplakia, especially in tobacco users).

Differential Diagnoses:

1. **Frictional keratosis (reactive hyperkeratosis):**
   - White plaque due to **chronic mechanical irritation (sharp teeth, ill-fitting dentures, habitual cheek biting).**
   - **Reversible** after eliminating the source of trauma.
2. **Oral lichen planus (reticular type):**
   - Presents as **bilateral, lace-like white striations (Wickham's striae).**
   - Affects **buccal mucosa, tongue, and gingiva**.
3. **Oral candidiasis (hyperplastic type):**
   - White, non-wipeable plaques with possible **fungal superinfection**.
   - Responds to **antifungal therapy** (e.g., nystatin, fluconazole).
4. **Oral hairy leukoplakia (OHL):**
   - White **corrugated plaques on the lateral tongue**.
   - Associated with **Epstein-Barr virus (EBV) and HIV/AIDS**.
5. **Early oral squamous cell carcinoma (OSCC):**
   - Any **non-homogeneous leukoplakia or speckled leukoplakia (erythroleukoplakia) raises suspicion.**
   - Biopsy is **mandatory** if lesions are persistent, thickened, or ulcerated.

Key Next Steps:

1. **Biopsy (gold standard for diagnosis):**
   - **Incisional biopsy** to assess dysplasia or malignant transformation.
   - Histopathology determines **degree of dysplasia (mild, moderate, severe).**
2. **Elimination of risk factors:**
   - **Smoking, alcohol, betel quid, HPV exposure**.
   - **Monitor for progression or worsening.**
3. **Management:**

    - **No dysplasia:** Conservative follow-up with risk factor modification.
    - **Mild dysplasia:** Regular monitoring (every 3-6 months).
    - **Moderate/severe dysplasia:** Consider **surgical excision, laser ablation, or cryotherapy**.
    - **Malignant transformation (OSCC detected):** Requires **oncologic evaluation and definitive treatment**.

Prognosis:

- **5-17% risk of malignant transformation** (higher for non-homogeneous leukoplakia or lesions in high-risk sites like the tongue and floor of the mouth).
- **Early detection and biopsy are critical for preventing progression to carcinoma.**

Would you like to discuss **biopsy techniques, leukoplakia grading, or early OSCC detection strategies?**

CASE 34

Reference:

https://media.springernature.com/lw685/springer-static/image/art%3A10.1007%2Fs12105-019-01003-7/MediaObjects/12105_2019_1003_Fig1_HTML.jpg

Chat-GPT4 answer:

The image shows a **small, pedunculated, cauliflower-like lesion on the hard palate**, with a **whitish surface** and no obvious ulceration or erythema.

Most Probable Diagnosis:

This lesion is **highly suggestive of a squamous papilloma**, a **benign epithelial proliferation caused by human papillomavirus (HPV), most commonly subtypes 6 and 11**.

Key Features of Squamous Papilloma:

- **Small, exophytic, papillary lesion**.
- **Pedunculated or sessile, with a verrucous or cauliflower-like surface**.
- **Usually asymptomatic, slow-growing**.
- Commonly found on the **palate, tongue, uvula, or buccal mucosa**.
- Associated with **HPV infection (low-risk subtypes 6 and 11).**

Differential Diagnoses:

1. **Verruca vulgaris (common wart):**
    - More common on **keratinized areas (hard palate, gingiva)**.
    - Often multiple and associated with **autoinoculation (fingers, lips).**
2. **Condyloma acuminatum:**
    - Associated with **HPV 6 and 11**, but larger and often **multiple, coalescing lesions**.
    - Typically found in the **genital region but can occur in the oral cavity**.
3. **Verrucous carcinoma:**
    - **Slow-growing, well-differentiated SCC variant**.
    - Larger, **locally aggressive**, and can **invade deeper tissues**.
    - Requires **biopsy for differentiation**.
4. **Focal epithelial hyperplasia (Heck's disease):**
    - Multiple small papillary lesions, often in **children or immunocompromised individuals**.
5. **Fibroepithelial polyp:**
    - Smooth, **pedunculated lesion without a papillary surface**.
    - More commonly caused by **trauma or chronic irritation**.

Key Next Steps:

1. **Excisional biopsy (definitive diagnosis):**

- Histopathology will confirm **papillary hyperplasia, koilocytes (HPV-infected cells), and benign squamous proliferation**.

2. **HPV Testing (optional but useful for high-risk patients).**
3. **Treatment:**
   - **Complete surgical excision** is **curative** with a low recurrence rate.
   - Laser excision or cryotherapy may be used for **multiple lesions**.
   - **Monitor for recurrence**, especially in **immunocompromised patients**.

Prognosis:

- **Excellent, as squamous papillomas are benign and rarely recur after excision.**
- **Low malignant transformation risk (unlike leukoplakia or verrucous carcinoma).**
- HPV-related lesions should prompt **discussion about HPV vaccination**.

Would you like to discuss **biopsy techniques, surgical management, or HPV-related oral lesions in more detail?**

CASE 35

Reference:
https://www.dentistavomero.it/wp-content/uploads/2023/02/papilloma-squamoso-orale-1024x560.jpg?x47274

Chat-GPT4 answer:

The image shows a **small, exophytic, pedunculated, and verrucous lesion on the hard palate**, with a **whitish, cauliflower-like surface**.

Most Probable Diagnosis:

This lesion is highly suggestive of a **squamous papilloma**, a **benign epithelial proliferation caused by human papillomavirus (HPV), most commonly subtypes 6 and 11**.

Key Features of Squamous Papilloma:

- **Small, exophytic, papillary lesion**, often **pedunculated**.
- **White or pink**, depending on keratinization.
- **Cauliflower-like surface** due to papillary projections.
- **Slow-growing, usually asymptomatic**.
- Most commonly found on the **palate, tongue, uvula, and buccal mucosa**.
- Associated with **HPV infection (low-risk subtypes 6 and 11).**

Differential Diagnoses:

1. **Verruca vulgaris (common wart):**
    - More common on **keratinized areas (palate, gingiva)**.
    - Often **multiple** and associated with **autoinoculation (fingers, lips).**
2. **Condyloma acuminatum:**
    - Associated with **HPV 6 and 11**, but tends to be **larger, broader, and often multiple**.
    - Typically found in the **genital region but can occur in the oral cavity**.
3. **Verrucous carcinoma:**
    - A **slow-growing, locally invasive variant of squamous cell carcinoma (SCC)**.
    - **Larger, non-pedunculated, and more aggressive**, requiring **biopsy for differentiation**.
4. **Focal epithelial hyperplasia (Heck's disease):**
    - Multiple small papillary lesions, often seen in **children or immunocompromised patients**.
5. **Fibroepithelial polyp:**
    - Smooth, **pedunculated lesion without a papillary surface**.
    - More commonly due to **trauma or chronic irritation**.

Key Next Steps:

1. **Excisional biopsy (definitive diagnosis):**
    - Histopathology will confirm **papillary hyperplasia, koilocytes (HPV-infected cells), and benign squamous proliferation**.

2. **HPV Testing (optional but useful for high-risk patients).**
3. **Treatment:**
   - **Complete surgical excision** is **curative** with a low recurrence rate.
   - Laser excision or cryotherapy may be used for **multiple lesions**.
   - **Monitor for recurrence**, especially in **immunocompromised patients**.

Prognosis:

- **Excellent, as squamous papillomas are benign and rarely recur after excision.**
- **Low malignant transformation risk (unlike leukoplakia or verrucous carcinoma).**
- HPV-related lesions should prompt **discussion about HPV vaccination**.

Would you like to discuss **biopsy techniques, surgical management, or HPV-related oral lesions in more detail?**

CASE 36

Reference:
https://www.blueridgedental.com/wp-content/uploads/2017/05/Oral-Cancer-Prevention-from-Top-Dentist-near-Minneapolis-Blue-Ridge-Dental-Center.jpg

Chat-GPT4 answer:

The image shows **irregular white patches with a reticulated pattern on the buccal mucosa**, without signs of ulceration, induration, or mass formation.

Most Probable Diagnosis:

This lesion is highly suggestive of **oral lichen planus (OLP)**, particularly the **reticular form**, which is the most common type.

Key Features of Oral Lichen Planus (OLP):

- **White, lace-like striations (Wickham's striae)**.
- Typically **bilateral and symmetrical**, affecting the **buccal mucosa, tongue, and gingiva**.
- **Chronic and immune-mediated**, associated with **T-cell-mediated autoimmunity**.
- Can be **asymptomatic or cause burning sensations, especially with spicy foods**.

Differential Diagnoses:

1. **Leukoplakia:**
   - Persistent **white plaques that do not rub off**.
   - Higher risk of **malignant transformation** (especially if non-homogeneous).
2. **Oral candidiasis (chronic hyperplastic type):**
   - White plaques that **do not wipe off** but may have **fungal superinfection**.
   - Responds to **antifungal treatment (e.g., nystatin, fluconazole).**
3. **Lupus erythematosus (discoid or systemic):**
   - White striae with **erythematous atrophic areas**.
   - Often associated with **systemic symptoms (photosensitivity, joint pain).**
4. **Oral hairy leukoplakia (OHL):**
   - White **corrugated plaques on the lateral tongue**.
   - Associated with **Epstein-Barr virus (EBV) and HIV/AIDS**.
5. **Chronic trauma/frictional keratosis:**
   - **More linear and associated with chronic irritation** (e.g., sharp teeth, dentures).
   - Resolves with elimination of trauma.

Key Next Steps:

1. **Clinical evaluation:**
   - Check for **bilateral involvement** (highly indicative of OLP).
   - Assess for **associated burning or sensitivity**.
2. **Biopsy (if uncertain or suspected dysplasia):**
   - Histopathology: **Saw-tooth rete ridges, lymphocytic infiltrate, liquefaction degeneration of basal cells.**

- Direct immunofluorescence (DIF): May help differentiate **OLP from lupus or pemphigoid.**

3. **Management:**
    - **Asymptomatic cases require no treatment but regular follow-ups.**
    - **Symptomatic cases:**
        - **Topical corticosteroids (clobetasol, fluocinonide).**
        - **Tacrolimus (calcineurin inhibitor) for refractory cases.**
        - **Avoidance of spicy foods, alcohol, and irritants.**
4. **Malignant Transformation Risk:**
    - **OLP has a 1-2% risk of malignant transformation to OSCC.**
    - Non-reticular forms (atrophic, erosive, ulcerative) have **higher risk**.

Prognosis:

- **Chronic condition with remissions and exacerbations.**
- Regular monitoring is essential due to **potential for malignant transformation**.

Would you like to discuss **OLP subtypes, biopsy techniques, or long-term management?**

CASE 37

Reference:

https://dentistchannel-online.s3.ap-south-1.amazonaws.com/photos/u2p9FuVXru2fYF1CnxewWardNQPbukZ4mBHsLAT8.jpg

Chat-GPT4 answer:

The image shows a **round, well-defined ulcer with a white fibrinous center and an erythematous halo on the palatal mucosa**.

Most Probable Diagnosis:

This lesion is highly suggestive of a **minor aphthous ulcer (recurrent aphthous stomatitis, RAS)**.

Key Features of Minor Aphthous Ulcers:

- **Small (<10 mm), round or oval ulcers** with a **yellowish/white fibrinous center** and **red inflamed borders**.
- **Painful**, especially with spicy, acidic, or hot foods.
- Typically occurs on **non-keratinized mucosa** (e.g., labial mucosa, buccal mucosa, soft palate).
- **Self-limiting**, resolving in **7-14 days** without scarring.
- Common in **young adults** and often **recurrent**.

Differential Diagnoses:

1. **Herpetic ulcers (Herpes Simplex Virus, HSV-1):**
    - **Multiple, clustered vesicles that rupture into ulcers**.
    - Common on **keratinized mucosa (gingiva, hard palate, lips)**.
    - Preceded by **prodromal symptoms (burning, tingling)**.
2. **Traumatic ulcer:**
    - Usually **irregular in shape** with **a history of local trauma** (e.g., sharp teeth, accidental biting).
    - Heals quickly after removal of the irritant.
3. **Oral lichen planus (erosive type):**
    - **Chronic, painful ulcers with surrounding Wickham's striae (reticular pattern)**.
    - Often **bilateral** and associated with an **immune-mediated response**.
4. **Squamous cell carcinoma (SCC):**
    - **Non-healing ulcer with induration, raised borders, or irregular surface**.
    - Persistent for **more than 2 weeks**, especially in **high-risk patients (smokers, alcohol consumers).**
    - **Biopsy is mandatory if persistent**.
5. **Behçet's disease:**
    - **Recurrent aphthous ulcers with genital ulcers and ocular involvement.**
    - More common in **Mediterranean and Middle Eastern populations**.

Management and Treatment:

- **Pain relief:**
  - **Topical corticosteroids (clobetasol, fluocinonide, dexamethasone rinse).**
  - **Topical anesthetics (lidocaine, benzocaine).**
- **Healing acceleration:**
  - **Chlorhexidine mouthwash** (antiseptic effect).
  - **Vitamin B12 supplementation** (deficiency is linked to aphthous ulcers).
- **Avoid triggers:**
  - **Spicy, acidic foods, sodium lauryl sulfate (SLS) in toothpaste**.
- **Severe or recurrent cases:**
  - **Systemic corticosteroids (prednisone for major aphthous ulcers).**
  - **Colchicine or thalidomide in refractory cases.**

Prognosis:

- **Self-limiting** and resolves within **1-2 weeks**.
- **Recurrent episodes are common**, but no risk of malignancy.

Would you like to discuss **triggers, systemic conditions associated with RAS, or differential diagnoses in more detail?**

CASE 38

Reference:

https://cwc-react.s3.ap-south-1.amazonaws.com/img/About+Cancer/BM01.jpg

Chat-GPT4 answer:

Clinical Analysis:

The image shows an **irregular, exophytic, white and erythematous lesion on the buccal mucosa**, with a **non-homogeneous, verrucous appearance**. The lesion appears to have a **thickened keratotic surface** with areas of **erythema and ulceration**, raising suspicion of a **potentially malignant disorder or early malignancy**.

Most Probable Diagnosis:

This lesion is **highly suggestive of a verrucous carcinoma**, which are **high-risk lesions for malignant transformation**.

Key Features Suggesting High Risk:

1. **Verrucous or exophytic growth:**
   - Possible **verrucous carcinoma**, a slow-growing, well-differentiated **variant of SCC**.
   - May also indicate **proliferative verrucous leukoplakia (PVL)**, which has a **high rate of malignant transformation**.
2. **Location:**
   - **Buccal mucosa and lateral tongue** are high-risk sites for oral squamous cell carcinoma (OSCC).
3. **Potential Malignant Transformation Indicators:**
   - **Ulceration or nodularity.**
   - **Induration or fixation.**
   - **Rapid growth or non-healing lesion (>2 weeks).**
   - **History of tobacco, alcohol, HPV, or betel quid use.**

Differential Diagnoses:

1. **Oral squamous cell carcinoma (OSCC):**
   - **Irregular, non-healing lesion with areas of ulceration, induration, and possible pain.**
   - Requires **urgent biopsy for histopathological confirmation**.
2. **Proliferative verrucous leukoplakia (PVL):**
   - **Multifocal, slowly progressive leukoplakia with high malignant potential.**
   - **High recurrence rate even after excision**.
3. **Verrucous carcinoma (VC):**
   - A well-differentiated variant of SCC, **locally aggressive but rarely metastatic**.
   - **Exophytic, wart-like, slow-growing lesion**.
4. **Chronic hyperplastic candidiasis:**
   - **White, thickened plaques that do not wipe off.**

    - **Biopsy with PAS stain to rule out fungal elements**.
5. **Oral lichen planus (erosive or plaque type):**
    - **Reticulated white striae with atrophic, ulcerative areas.**
    - **Often bilateral, may cause burning pain**.

Key Next Steps:

1. **Urgent Incisional Biopsy:**
    - **Histopathology to confirm dysplasia, carcinoma in situ, or invasive carcinoma**.
    - **Immunohistochemistry for p53, Ki-67 if needed**.
2. **Eliminate Risk Factors:**
    - **Smoking, alcohol, betel quid, HPV exposure.**
    - **Monitor for recurrence after excision.**
3. **Definitive Treatment (Depending on Biopsy Results):**
    - **Moderate/Severe Dysplasia:** Consider **surgical excision or laser therapy**.
    - **OSCC/VC:** Requires **oncologic management (surgery ± radiotherapy ± chemotherapy).**
    - **PVL:** Requires **long-term follow-up** due to high recurrence.

Prognosis:

- **Leukoplakia with dysplasia: 5-30% risk of malignant transformation**.
- **PVL:** Up to **70% malignant transformation over time**.
- **Verrucous carcinoma: Locally invasive but rarely metastasizes**.
- **OSCC:** Prognosis depends on **tumor stage, nodal involvement, and early intervention**.

Conclusion:

- **This lesion requires an urgent biopsy to determine malignancy risk.**
- **If biopsy confirms dysplasia or SCC, early treatment is critical to improving prognosis.**
- **Long-term follow-up is essential due to recurrence risk.**

Would you like to discuss **biopsy techniques, surgical options, or adjuvant therapies?**

CASE 39

Reference:

https://www.aaom.com/assets/condition-information/oral%20lichen%20planus%201.jpg

Chat-GPT4 answer:

Clinical Analysis:

The image shows **white, reticulated, lace-like striations (Wickham's striae) on the buccal mucosa**, characteristic of **oral lichen planus (OLP)**.

Most Probable Diagnosis:

This lesion is highly suggestive of **reticular oral lichen planus (OLP)**, a **chronic immune-mediated mucosal disorder**.

Key Features of Oral Lichen Planus (OLP):

- **White, lace-like striations (Wickham's striae) on the buccal mucosa**.
- **Bilateral and symmetrical distribution**.
- Commonly affects the **buccal mucosa, tongue, and gingiva**.
- Chronic, **immune-mediated disease** (T-cell-mediated).
- **Asymptomatic or mild burning sensation**.

Differential Diagnoses:

1. **Leukoplakia:**
    - **Homogeneous white patch**, no lace-like pattern.
    - **Higher risk of malignant transformation**.
2. **Oral candidiasis (chronic hyperplastic):**
    - White plaques that **do not wipe off**.
    - May respond to **antifungal therapy**.
3. **Lupus erythematosus (discoid or systemic):**
    - White striae with **erythematous atrophic areas**.
    - Often associated with **systemic symptoms (photosensitivity, joint pain).**
4. **Oral hairy leukoplakia (OHL):**
    - **White, corrugated plaques** on the lateral tongue.
    - Associated with **Epstein-Barr virus (EBV) and HIV/AIDS**.
5. **Frictional keratosis (chronic trauma):**
    - **More linear, no Wickham's striae**, resolves after eliminating trauma.

Key Next Steps:

1. **Clinical Evaluation:**
    - Check for **bilateral involvement** (highly indicative of OLP).
    - Assess for **associated burning or sensitivity**.

2. **Biopsy (if uncertain or suspected dysplasia):**
    - **Histopathology:** Saw-tooth rete ridges, basal layer degeneration, lymphocytic infiltrate.
    - **Direct immunofluorescence (DIF):** Helps differentiate **OLP from lupus or pemphigoid**.
3. **Management:**
    - **Asymptomatic cases:** No treatment, regular follow-up.
    - **Symptomatic cases:**
        - **Topical corticosteroids (clobetasol, fluocinonide).**
        - **Tacrolimus (calcineurin inhibitor) for refractory cases.**
        - **Chlorhexidine mouthwash** for secondary infections.
4. **Malignant Transformation Risk:**
    - **OLP has a 1-2% risk of malignant transformation to OSCC.**
    - Non-reticular forms (atrophic, erosive) have **higher risk**.

Prognosis:

- **Chronic condition** with remissions and exacerbations.
- **Regular monitoring is essential** due to **potential for malignant transformation**.

Would you like to discuss **OLP subtypes, biopsy techniques, or long-term management?**

CASE 40

Reference:

https://www.mdpi.com/ijms/ijms-23-13099/article_deploy/html/images/ijms-23-13099-g001.png

Chat-GPT4 answer:

Clinical Analysis:

The image presents **bilateral white, reticular, lace-like striations on the buccal mucosa**, which are characteristic of **oral lichen planus (OLP)**. There are also areas of **erythema and erosion**, suggesting a more symptomatic or erosive form.

Most Probable Diagnosis:

This lesion is highly suggestive of **oral lichen planus (OLP)**, particularly the **reticular and erosive subtypes**.

Key Features Suggesting Oral Lichen Planus (OLP):

✅ **Reticular White Striae (Wickham's Striae):**

- Interlacing **white keratotic lines** on the buccal mucosa.
- Classically **bilateral and symmetrical**.

✅ **Erosive/Atrophic Areas (Right Side of Image):**

- **Reddened, ulcerated areas** → Indicates **erosive OLP**.
- More likely to cause **pain and discomfort**.

✅ **Chronic Nature:**

- Long-term **waxing and waning course**.
- T-cell-mediated immune response.

✅ **Commonly Affected Sites:**

- Buccal mucosa (**most frequent**).
- Tongue, gingiva, and palate.

Differential Diagnoses:

1️⃣ Leukoplakia:

- Typically **unilateral, well-demarcated, homogeneous or speckled**.
- Higher malignant transformation potential.

2️⃣ Lupus Erythematosus (Oral Lesions):

- Can have **reticular striae** but usually more **atrophic or ulcerated**.
- May involve **systemic symptoms (photosensitivity, arthritis, etc.).**

3️⃣ Chronic Hyperplastic Candidiasis:

- White plaques that **do not wipe off**.
- **Responds to antifungal therapy.**

4️⃣ Oral Graft-Versus-Host Disease (GVHD):

- Similar striations but in **post-transplant patients**.
- More **diffuse mucosal involvement**.

5️⃣ Oral Dysplasia / Early SCC (if ulcerative areas persist):

- Requires **biopsy if erosions persist >2 weeks**.

Management & Follow-Up:

📌 **Diagnosis Confirmation:**

- **Biopsy if uncertain** (Histology: **basal layer liquefaction, lymphocytic infiltrate**).
- **Direct Immunofluorescence (DIF)** → Differentiates from **Lupus or Pemphigoid**.

📌 **Treatment Options (Based on Symptoms):**

- **Asymptomatic Reticular OLP** → No treatment, but regular monitoring.
- **Symptomatic (Erosive/Ulcerative)**:
  - **First-line: Topical Corticosteroids (Clobetasol, Fluocinonide, Betamethasone).**
  - **Second-line: Topical Calcineurin Inhibitors (Tacrolimus, Pimecrolimus) for steroid-resistant cases.**
  - **Chlorhexidine mouthwash** to prevent secondary infections.

📌 **Long-Term Monitoring:**

- **1-2% Malignant Transformation Risk** → Higher in erosive OLP.
- **Regular follow-ups every 6 months** to monitor progression.

Conclusion:

- This lesion is **highly suggestive of oral lichen planus** (reticular and erosive forms).
- **Requires monitoring** due to risk of malignant transformation.
- **Treatment depends on symptoms** (topical steroids for erosive cases).

Would you like to discuss **biopsy criteria, malignant transformation risks, or advanced treatment options?**

CASE 41

Reference:

https://i0.wp.com/pocketdentistry.com/wp-content/uploads/2020/06/10-1055-b-006-161159_c023_f004-1.jpg?fit=498%2C653&ssl=1

Chat-GPT4 answer:

Clinical Analysis:

The image shows an **irregular, ulcerated, exophytic mass with a necrotic center and erythematous inflamed borders**, suggestive of **a malignant or aggressive neoplastic process** in the oropharyngeal or oral mucosa.

Most Probable Diagnosis:

The lesion strongly suggests **oral squamous cell carcinoma (OSCC)** due to its: ✓ **Irregular, exophytic, ulcerated mass**
✓ **Necrotic areas and induration**
✓ **Erythematous, inflamed periphery**
✓ **Infiltrative nature with possible tissue destruction**

Differential Diagnoses:

1️⃣ **Oral Squamous Cell Carcinoma (OSCC)**

- Most common malignancy of the oral cavity.
- Risk factors: **Tobacco, alcohol, HPV, betel nut, immunosuppression**.
- **Histology:** Dysplastic epithelium, keratin pearls, invasive nests.

2️⃣ **Verrucous Carcinoma**

- Exophytic, slow-growing, **cauliflower-like appearance**.
- **Less aggressive, rare metastasis**, but locally invasive.

3️⃣ **Deep Fungal Infection (e.g., Mucormycosis)**

- **Necrotic ulcer with black eschar**, often in immunocompromised patients.
- **Biopsy + PAS/GMS stain** for fungal elements.

4️⃣ **Tuberculous Ulcer**

- Chronic **non-healing ulcer** with **caseating granulomas**.
- Associated with **pulmonary TB**.

5️⃣ Necrotizing Sialometaplasia

- **Benign, self-limiting ulcerative lesion**, mimicking SCC.
- **Biopsy essential** to exclude malignancy.

Essential Investigations:

✅ **Incisional biopsy** → Confirm SCC histopathology.
✅ **CT/MRI** → Tumor extent, bone invasion, lymph node involvement.
✅ **HPV testing** → If located in the oropharynx.
✅ **PET-CT** → If advanced disease is suspected.

Management Plan (if SCC is confirmed):

📌 **Early-stage (T1-T2, N0):**

- **Surgical excision with clear margins**
- +/- **Selective neck dissection**

📌 **Locally advanced (T3-T4, N+):**

- **Surgery + adjuvant radiotherapy +/- chemotherapy**
- Cisplatin-based regimens for advanced cases.

📌 **Metastatic:**

- **Palliative chemo/immunotherapy (PD-1 inhibitors, Cetuximab)**

Prognosis & Follow-up:

- **5-year survival rate** depends on **TNM staging** (80% early, <30% advanced).
- **Regular follow-ups (every 3 months first 2 years)** due to **high recurrence risk**.

Conclusion:

- **High suspicion for OSCC**.
- **Urgent biopsy required** for definitive diagnosis.

- **Multidisciplinary approach (surgery, oncology, radiation therapy).**

Would you like guidance on **biopsy techniques or treatment algorithms** for advanced cases?

CASE 42

Reference:

https://www.bjmp.org/files/2016-9-4/bjmp-2016-9-4-a930e.jpg

Chat-GPT4 answer:

Clinical Analysis:

The image reveals **exposed necrotic bone in the posterior mandible**, with evidence of **adjacent mucosal ulceration and lack of healing**, suggestive of **medication-related osteonecrosis of the jaw (MRONJ)**.

Most Probable Diagnosis:

This presentation is highly **suggestive of MRONJ** (Medication-Related Osteonecrosis of the Jaw), particularly in patients with: ✓ **History of antiresorptive therapy** (Denosumab, Bisphosphonates). ✓ **Non-healing mucosal ulceration with exposed bone ≥ 8 weeks**. ✓ **Absence of prior radiation therapy or metastatic disease to the jaws**.

Differential Diagnoses:

1️⃣ Osteoradionecrosis (ORN)

- Occurs **post-radiation therapy (>50 Gy) to head & neck**.
- More fibrotic, widespread necrosis, and **poor vascular supply**.

2️⃣ Chronic Suppurative Osteomyelitis

- Associated with **bacterial infection**, **pain**, and **purulent discharge**.
- Often follows **untreated odontogenic infections**.

3️⃣ Primary or Metastatic Bone Malignancy

- Consider **squamous cell carcinoma or metastatic lesions** (breast, lung, prostate).
- **Biopsy required** to rule out malignancy.

Essential Investigations:

✅ **History:**

- **Anti-resorptive drug use (Denosumab, Zoledronate, Alendronate)?**
- **Prior dental extractions or surgical trauma?**
- **Any malignancy (multiple myeloma, breast cancer, osteoporosis treatment)?**

✅ **Imaging:**

- **Panoramic X-ray / CBCT** → Detect **sequestrum, osteolysis, periosteal reaction**.
- **MRI** → Evaluate **soft tissue involvement**.
- **Bone Scan (SPECT-CT) if extensive necrosis suspected**.

✅ **Biopsy (if atypical presentation):**

- **Exclude malignancy or osteomyelitis.**

Management (Based on AAOMS 2022 Staging):

Stage 1 (Asymptomatic Exposed Bone)

🔷 **Oral hygiene, chlorhexidine rinses, conservative follow-up.**

Stage 2 (Painful Exposed Bone + Secondary Infection)

🔷 **Antibiotics (Amoxicillin ± Metronidazole) + Antiseptic rinses.**
🔷 **Avoid aggressive debridement unless necessary.**

Stage 3 (Extensive Bone Exposure with Pathologic Fracture)

🔷 **Surgical debridement/sequestrectomy** if **severe necrosis.**
🔷 **Systemic antibiotics (IV therapy in advanced cases).**

📌 **Discontinue Denosumab/Bisphosphonates if feasible** (case-by-case).
📌 **Hyperbaric oxygen therapy?** → Consider **only in refractory cases.**

Prognosis & Follow-up:

- **MRONJ may progress despite conservative management.**
- **Regular monitoring (every 3-6 months) to prevent worsening.**
- **Prevention is key: Avoid extractions, optimize oral hygiene before starting antiresorptive therapy.**

Conclusion:

🔎 **Strongly suggestive of MRONJ (likely Stage 2).**
🔎 **Urgent dental/surgical evaluation needed to prevent further complications.**
🔎 **CT/MRI imaging recommended before definitive management.**

Would you like a **flowchart for MRONJ management**, or details on **advanced surgical options** (e.g., segmental resection vs. conservative debridement)?

CASE 43

Reference:

https://www.chirurgiafacciale.com/wp-content/uploads/2023/09/mascellare-1-1024x873.webp

Chat-GPT4 answer:

Analysis and Probable Diagnosis

Observations:

1. **Exposed necrotic bone**:
   - There is an area of exposed bone in the posterior maxilla with a necrotic appearance.
   - Yellowish-white sequestra are visible, surrounded by inflamed and hyperemic mucosa.
2. **Surrounding inflammatory response**:
   - The adjacent tissue appears erythematous with a hyperplastic, irregular border.
   - Some areas show granulation tissue, potentially indicating a chronic inflammatory response.
3. **Foamy secretion or exudate**:
   - Presence of bubbles or a foamy appearance, which might indicate bacterial colonization or infection.

Most Probable Diagnosis:

Medication-Related Osteonecrosis of the Jaw (MRONJ)

- Given the characteristic findings of exposed necrotic bone, surrounding inflammatory response, and absence of healing mucosa, this lesion is highly suggestive of **MRONJ**.
- It appears to be in **Stage 2**, as defined by the **AAOMS staging system**, due to the presence of infection and inflammation around the necrotic bone.

Differential Diagnoses to Consider:

1. **Chronic osteomyelitis**:
   - Typically presents with bone necrosis, but usually follows an odontogenic infection rather than antiresorptive therapy.
2. **Squamous cell carcinoma (SCC) of the maxilla**:
   - A biopsy should be considered if the lesion has been progressive, shows non-healing ulceration, or if paresthesia is present.
3. **Fungal osteomyelitis (Mucormycosis or Aspergillosis)**:
   - Especially relevant in immunocompromised patients, but typically associated with black eschar formation.

Recommended Next Steps:

1. **Detailed history taking**:

  - **Medications**: Denosumab, bisphosphonates, antiangiogenic agents?
  - **Previous dental procedures**: Extractions or trauma to the area?
  - **Systemic conditions**: Diabetes, malignancies, or immunosuppression?
2. **Microbiological evaluation**:
  - Culture if secondary infection is suspected.
3. **Imaging**:
  - **CBCT** or **MRI** to assess the extent of bone involvement and rule out malignancy.
4. **Biopsy**:
  - If malignancy is a concern, a biopsy is necessary to rule out SCC.

Would you like a **detailed management protocol for MRONJ Stage 2**, including pharmacological and surgical strategies?

CASE 44

Reference:

https://screening.iarc.fr/picoral/B2b00024.jpg

Chat-GPT4 answer:

Analysis and Probable Diagnosis

Observations:

1. **Ulcerative, exophytic mass**:
   - The lesion in the posterior maxilla appears **irregular, ulcerated, and exophytic**, with a mix of red and white areas.
   - The lesion has a **friable, granular surface** that suggests rapid cellular proliferation.
2. **Necrotic and inflammatory areas**:
   - There are **yellowish necrotic zones**, possibly indicating tissue breakdown or secondary infection.
   - Surrounding **erythematous mucosa** suggests an inflammatory response.
3. **Bone involvement**:
   - The lesion appears to be affecting the **posterior maxilla** with potential extension into deeper structures.
   - This could suggest **bone invasion**, which is concerning for malignancy.

Most Probable Diagnosis: Squamous Cell Carcinoma (SCC) of the Maxilla

- The **irregular, ulcerative mass with necrosis and induration** is highly suggestive of **oral squamous cell carcinoma (OSCC)**.
- SCC is the **most common malignant tumor of the oral cavity**, often presenting with **non-healing ulcers, pain, and local tissue destruction**.

Differential Diagnoses to Consider:

1. **Necrotizing sialometaplasia**:
   - Benign self-limiting condition but typically **painless** and lacks an exophytic component.
2. **Chronic osteomyelitis with sequestration**:
   - Would present with **exposed bone** rather than a proliferative mass.
3. **Medication-Related Osteonecrosis of the Jaw (MRONJ)**:
   - Typically shows **exposed necrotic bone**, but in advanced stages, superinfection and inflammatory tissue can mimic malignancy.
   - Requires **history of antiresorptive medication** (bisphosphonates, denosumab).
4. **Deep fungal infections (Mucormycosis, Aspergillosis)**:
   - Often present with **black necrotic eschars** rather than ulceroproliferative lesions.
   - More common in immunocompromised patients (e.g., diabetes, chemotherapy).

Recommended Next Steps:

1. **Urgent Biopsy**:
   - Incisional biopsy to confirm malignancy and histological grading.
2. **Imaging**:
   - **Contrast-enhanced CT or MRI** to assess **bone invasion, perineural spread, and soft tissue extension**.
   - **PET-CT** if SCC is confirmed, to evaluate metastasis.
3. **TNM Staging**:
   - If SCC is diagnosed, staging according to **AJCC TNM classification** is necessary.
4. **Multidisciplinary Approach**:
   - Referral to **Head and Neck Oncology**, involving surgery, radiation, and possibly chemotherapy based on staging.

Conclusion

This lesion is highly suspicious for **oral squamous cell carcinoma (OSCC)** and requires **urgent biopsy and imaging**. Would you like assistance with **TNM staging criteria or treatment protocols** for OSCC?

CASE 45

Reference:

https://domenicotesi.it/wp-content/uploads/2020/08/EPULIDE-GRAVIDICA.jpg

Chat-GPT4 answer:

Analysis and Probable Diagnosis

Observations:

1. **Exophytic, vascular lesion on the gingiva**:
   - The lesion appears **pedunculated and reddish-purple**, suggesting a high degree of vascularity.
   - It is localized on the **interdental gingiva**.
2. **Smooth or lobulated surface**:
   - The lesion does not appear ulcerated, and it has a **smooth, shiny, or slightly lobulated surface**.
3. **Gingival inflammation in surrounding tissue**:
   - The adjacent gingiva appears **erythematous**, indicating a possible reactive inflammatory response.

Most Probable Diagnosis: Pyogenic Granuloma

- Pyogenic granuloma (also known as **lobular capillary hemangioma**) is a **common reactive lesion** that occurs due to **chronic irritation, trauma, or hormonal influences (e.g., pregnancy, puberty).**
- It is **not a true granuloma** but rather a **vascular proliferation** caused by local irritation factors such as plaque, calculus, or minor trauma.

Differential Diagnoses to Consider:

1. **Peripheral Giant Cell Granuloma (PGCG)**:
   - Typically has a **bluish-purple** hue and may show **bone resorption on radiographs**.
   - More common in **edentulous or older patients**.
2. **Peripheral Ossifying Fibroma (POF)**:
   - More fibrotic, often has **calcifications** on histology.
   - Less vascular than pyogenic granuloma.
3. **Kaposi's Sarcoma (KS)**:
   - More common in **HIV-positive** or **immunocompromised patients**.
   - Typically **multifocal** and may extend beyond the gingiva.
4. **Squamous Cell Carcinoma (SCC) of the gingiva**:
   - More **irregular, indurated, ulcerated**, and less vascular.
   - Needs to be ruled out if the lesion **does not regress** after excision.

Recommended Next Steps:

1. **Patient History and Clinical Correlation**:
    - History of **recent trauma, irritation (e.g., orthodontic brackets, restorations), or hormonal changes**?
    - **Pregnancy status** in female patients, as pyogenic granuloma is common in pregnancy.
2. **Radiographic Examination**:
    - **Periapical or panoramic radiograph** to check for **bone involvement** (rules out PGCG or POF).
3. **Excisional Biopsy with Histopathology**:
    - Complete surgical excision with **removal of any underlying irritants** (plaque, calculus, or defective restorations).
    - Histology will confirm **proliferating capillaries, inflammatory infiltrate, and absence of malignancy**.
4. **Recurrence Prevention**:
    - **Good oral hygiene and plaque control** to reduce recurrence risk.
    - If related to pregnancy, **spontaneous regression** can occur postpartum.

Conclusion

The lesion is **highly suggestive of a Pyogenic Granuloma**, a benign vascular overgrowth due to chronic irritation. Would you like a **detailed excision protocol** or a **discussion on recurrence management**?

CASE 46

Reference:

https://www.odontoiatria33.it/cont/media/Foto_articoli/Dental_Cadmos_2007/de_biase.jpg

Chat-GPT4 answer:

Analysis and Probable Diagnosis

Observations:

1. **Well-defined, pedunculated gingival lesion**:
   - The lesion is **smooth, round, and reddish-pink**, suggesting a **vascular nature**.
   - It is attached to the gingiva between the **lower anterior teeth**.
2. **Localized inflammatory response**:
   - The surrounding gingival margin appears **erythematous**.
   - There is **gingival recession** and possible **plaque or calculus accumulation**, which might be contributing factors.
3. **No ulceration or necrosis**:
   - The surface of the lesion is intact, with no apparent **ulceration, induration, or necrotic areas**.

Most Probable Diagnosis: Pyogenic Granuloma

- **Reactive hyperplastic lesion** due to chronic irritation from **poor oral hygiene, calculus, or minor trauma**.
- Highly vascular and **often seen in pregnant women** due to hormonal influences (**pregnancy tumor**).
- Typically **benign** and does not show aggressive behavior.

Differential Diagnoses to Consider:

1. **Peripheral Giant Cell Granuloma (PGCG)**:
   - Usually more **bluish-purple** and may involve **underlying bone** (requires radiographic evaluation).
   - Histologically contains **multinucleated giant cells**.
2. **Peripheral Ossifying Fibroma (POF)**:
   - Firmer in consistency, may contain **calcifications**.
   - More fibrotic and less vascular than pyogenic granuloma.
3. **Squamous Cell Carcinoma (SCC)**:
   - Unlikely, as SCC lesions are typically **irregular, ulcerated, and indurated**.
   - SCC should be considered if the lesion **does not resolve after excision**.
4. **Kaposi's Sarcoma (KS)**:
   - More common in **HIV-positive or immunocompromised patients**.
   - Appears **multifocal**, often involving the **palate and gingiva**.

Recommended Next Steps:

1. **Patient History and Clinical Correlation**:
    - **History of trauma, pregnancy, or local irritation**?
    - Assessment of **hormonal influences** (common in pregnancy).
2. **Radiographic Examination**:
    - **Periapical radiograph or panoramic X-ray** to rule out **bony involvement**.
3. **Excisional Biopsy with Histopathology**:
    - Recommended for **definitive diagnosis and to prevent recurrence**.
    - Ensures **no underlying neoplastic changes**.
4. **Management & Prevention**:
    - **Complete excision** including the **base of the lesion**.
    - **Scaling and root planing** to eliminate any contributing local irritants.
    - **Oral hygiene reinforcement** to prevent recurrence.

Conclusion

This lesion is **highly suggestive of a Pyogenic Granuloma**, a common reactive gingival overgrowth. Would you like a **detailed excision protocol** or a **discussion on recurrence prevention strategies**?

CASE 47

Reference:

https://pub.mdpi-res.com/curroncol/curroncol-30-00767/article_deploy/html/images/curroncol-30-00767-g001.png?1702880941

Chat-GPT4 answer:

Analysis and Probable Diagnosis

Observations:

1. **Ulcerative, exophytic lesion with necrosis**:
   - The lesion on the **mandibular alveolar ridge** appears **irregular, lobulated, and ulcerative**.
   - Areas of **necrotic tissue** and **granulation-like hyperplasia** are evident.
2. **Poor oral hygiene and dental status**:
   - The adjacent teeth show **advanced periodontal disease, severe plaque accumulation, and possible mobility**.
   - The lesion might have developed in association with chronic irritation or infection.
3. **Bone exposure and possible sequestration**:
   - The lesion appears to have **underlying bone involvement**, suggesting an aggressive or chronic condition affecting the mandible.

Most Probable Diagnosis: Squamous Cell Carcinoma (SCC) of the Gingiva or Alveolar Ridge

- The irregular, ulcerative, and indurated nature of the lesion is **highly suspicious for oral squamous cell carcinoma (OSCC)**.
- OSCC commonly affects the **gingiva and alveolar mucosa**, especially in **elderly patients with poor dental status**.
- Mandibular involvement suggests **bone invasion**, a feature of advanced SCC.

Differential Diagnoses to Consider:

1. **Medication-Related Osteonecrosis of the Jaw (MRONJ)**:
   - If the patient has a **history of bisphosphonates, denosumab, or antiangiogenic therapy**, MRONJ should be considered.
   - Typically, MRONJ presents with **exposed necrotic bone**, while this lesion appears **more proliferative and ulcerated**.
2. **Chronic Osteomyelitis with Sequestration**:
   - Possible in long-standing **odontogenic infections or periodontal disease**.
   - Unlike SCC, it **lacks an exophytic component** and typically presents with **pain, pus discharge, and fistula formation**.
3. **Deep Fungal Infections (Mucormycosis, Aspergillosis)**:
   - If the patient is **immunocompromised (e.g., diabetes, chemotherapy, corticosteroids use)**, fungal osteomyelitis could be considered.
   - More likely in cases with **black necrotic eschars** and rapid tissue destruction.

Recommended Next Steps:

1. **Urgent Biopsy**:
    - Incisional biopsy is **mandatory** to confirm malignancy and assess histopathology.
    - Immunohistochemical staining may be needed for definitive diagnosis.
2. **Imaging**:
    - **Contrast-enhanced CT/MRI** to assess:
        - **Bone invasion and soft tissue spread**.
        - **Perineural involvement**.
    - **PET-CT** if SCC is confirmed, to evaluate **metastasis**.
3. **Multidisciplinary Evaluation**:
    - Referral to **Head and Neck Oncology** for staging and treatment planning.
    - If SCC is confirmed, management may include:
        - **Surgical resection** with **segmental mandibulectomy** if bone invasion is present.
        - **Neck dissection** if nodal involvement is suspected.
        - **Adjuvant radiotherapy and/or chemotherapy** depending on TNM staging.

Conclusion

This lesion is **highly suspicious for squamous cell carcinoma (SCC) of the gingiva/alveolar ridge**, requiring **urgent biopsy and imaging**. Would you like guidance on **TNM staging, treatment protocols, or surgical approaches** for SCC?

CASE 48

Reference:

https://marianiumberto.it/wp-content/uploads/2023/09/CARCINOMA-PERIMPLANTARE-1-1030x687.jpg

Chat-GPT4 answer:

Analysis and Probable Diagnosis

Observations:

1. **Irregular, exophytic lesion with ulceration and induration**:
   - The lesion appears **erythematous with a lobulated, proliferative surface**.
   - There are **areas of ulceration and necrosis**, with a **firm, indurated base**, raising suspicion of malignancy.
2. **Association with a dental implant**:
   - The presence of a **dental implant near the lesion** suggests a possible **peri-implant pathology**.
   - The lesion could be a **reactive lesion due to chronic irritation**, or a more serious condition like **peri-implant malignancy**.
3. **Surrounding mucosal inflammation**:
   - The adjacent mucosa appears **erythematous and hyperplastic**, possibly due to **chronic irritation, infection, or neoplastic infiltration**.
   - **Bone loss** around the implant is highly probable and should be evaluated radiographically.

Most Probable Diagnosis: Squamous Cell Carcinoma (SCC) of the Mandibular Mucosa

- The **ulcerated, exophytic, and indurated nature** of the lesion is highly suggestive of **oral squamous cell carcinoma (OSCC)**.
- SCC is the **most common malignancy affecting the oral cavity**, often associated with **chronic irritation, previous trauma, or peri-implant complications**.
- If the implant was placed after **previous extractions for a premalignant lesion**, this could indicate **implant-associated malignancy**.

Differential Diagnoses to Consider:

1. **Peri-Implantitis with Hyperplastic Granulation Tissue**:
   - Typically presents with **erythema, swelling, and pus discharge**.
   - However, it does not usually have **firm induration or ulceration**, as seen in this lesion.
2. **Medication-Related Osteonecrosis of the Jaw (MRONJ)**:
   - If the patient has a **history of bisphosphonates or denosumab**, osteonecrosis should be considered.
   - MRONJ presents with **exposed necrotic bone**, while this lesion appears more proliferative.
3. **Chronic Traumatic Ulcer/Hyperplasia**:

- Could result from **chronic irritation** from the implant or adjacent teeth.
- Unlike SCC, traumatic ulcers **resolve after removing the irritant**, whereas SCC persists and progresses.

4. **Metastatic Carcinoma**:
   - If the patient has a history of **lung, breast, or renal cancer**, a metastatic lesion should be ruled out.
   - **Biopsy with immunohistochemistry (IHC)** can help differentiate **primary SCC from metastatic carcinoma**.

Recommended Next Steps:

1. **Urgent Incisional Biopsy**:
   - **Histopathological examination is essential** to confirm malignancy.
   - If SCC is diagnosed, **immunohistochemistry (p16, CK5/6, p63) can help differentiate subtypes**.
2. **Imaging Studies**:
   - **Contrast-enhanced CT or MRI** to evaluate **bone invasion, perineural spread, and soft tissue involvement**.
   - **PET-CT scan** if SCC is confirmed, to check for **regional and distant metastasis**.
3. **TNM Staging**:
   - **Based on tumor size, nodal involvement, and metastasis (AJCC staging system)**.
   - If lymph nodes are involved, a **neck dissection** may be required.
4. **Multidisciplinary Management**:
   - Referral to **Head and Neck Oncology** for staging and treatment planning.
   - If SCC is confirmed, treatment may involve:
     - **Surgical resection (segmental mandibulectomy if bone invasion is present)**.
     - **Neck dissection** if lymph nodes are involved.
     - **Adjuvant radiotherapy and/or chemotherapy** based on stage.

Conclusion

This lesion is **highly suspicious for oral squamous cell carcinoma (OSCC)**, requiring **urgent biopsy and imaging** to confirm the diagnosis and plan appropriate treatment. Would you like assistance with **TNM staging, surgical management options, or treatment protocols** for OSCC?

CASE 49

Reference:
https://media.springernature.com/lw685/springer-static/image/chp%3A10.1007%2F978-981-15-5483-4_45/MediaObjects/484230_1_En_45_Figa_HTML.jpg

Chat-GPT4 answer:

Analysis and Probable Diagnosis

Observations:

1. **Large, exophytic, ulcerated mass on the lateral border of the tongue**:
   - The lesion is **irregular, fungating, and lobulated**, with **erythematous and ulcerated areas**.
   - There are **white, keratotic regions mixed with necrotic tissue**.
   - The lesion appears **firm and indurated**, with evidence of **infiltration into surrounding tissues**.
2. **High-Risk Location: Lateral Border of the Tongue**:
   - The **posterolateral tongue** is a **high-risk site for malignancy**.
   - The lesion's **size, irregular surface, ulceration, and firm consistency** raise concern.
3. **Poor Oral Hygiene and Tobacco Staining on Teeth**:
   - The **severely stained, worn dentition** suggests **a history of smoking or betel nut chewing**, which are strong **risk factors for oral cancer**.

Most Probable Diagnosis: Squamous Cell Carcinoma (SCC) of the Tongue

- **Oral squamous cell carcinoma (OSCC)** is the **most common malignancy affecting the oral cavity**.
- The lateral border of the tongue is a **common site due to chronic exposure to carcinogens (tobacco, alcohol, betel nut)**.
- The lesion's **exophytic nature, ulceration, induration, and possible fixation** are highly suspicious for malignancy.

Differential Diagnoses to Consider:

1. **Verrucous Carcinoma (If Slow-Growing and Wart-Like)**
   - A **low-grade variant of SCC**, often associated with **chronic tobacco use**.
   - Presents as a **slow-growing, exophytic, cauliflower-like lesion**.
   - Unlike SCC, **less tendency for deep invasion or metastasis**.
2. **Chronic Traumatic Ulcer (If Associated with Mechanical Trauma)**
   - Would have **a clear history of repeated trauma (sharp teeth, ill-fitting dentures)**.
   - Typically **smaller and resolves after the removal of the source of trauma**.
   - **Biopsy is required if the lesion persists for >2 weeks**.
3. **Syphilitic Ulcer (If Associated with Systemic Symptoms)**
   - **Primary syphilitic chancre** can present as a **painless ulcer**, while **tertiary syphilis can cause gummatous lesions**.
   - **Serological testing (FTA-ABS, VDRL) is needed if suspected**.
4. **Tuberculous Ulcer (If Associated with Pulmonary TB)**

- Presents as a **chronic, non-healing ulcer** with **undermined edges**.
- Requires **histopathological confirmation of granulomas and AFB staining**.

Recommended Next Steps:

1. **Urgent Incisional Biopsy**:
   - **Histopathological examination is essential** to confirm malignancy.
   - If SCC is diagnosed, **immunohistochemistry (p16, CK5/6, p63) can help differentiate subtypes**.
2. **Imaging Studies**:
   - **Contrast-enhanced CT or MRI** to evaluate **tumor extent, bone invasion, and perineural spread**.
   - **PET-CT scan** if SCC is confirmed, to check for **regional and distant metastasis**.
3. **TNM Staging (AJCC Staging System)**:
   - Based on **tumor size, nodal involvement, and metastasis**.
   - If lymph nodes are involved, a **neck dissection** may be required.
4. **Multidisciplinary Management**:
   - Referral to **Head and Neck Oncology** for staging and treatment planning.
   - If SCC is confirmed, treatment may involve:
     - **Surgical resection (partial glossectomy or hemiglossectomy if extensive involvement)**.
     - **Neck dissection (if lymph nodes are involved)**.
     - **Adjuvant radiotherapy and/or chemotherapy** based on stage.

Conclusion

This lesion is **highly suspicious for squamous cell carcinoma (SCC) of the tongue**, requiring **urgent biopsy and imaging** to confirm the diagnosis and plan appropriate treatment. Would you like assistance with **TNM staging, surgical management options, or treatment protocols for OSCC?**

CASE 50

Reference:
https://media-us.amboss.com/media/thumbs/big_5081d952cf2c7.jpg

Chat-GPT4 answer:

Analysis and Probable Diagnosis

Observations:

1. **Exophytic, ulcerated, and irregular mass on the floor of the mouth**:
    - The lesion is **ulcerated, indurated, and has an irregular granular surface with areas of necrosis**.
    - Surrounding mucosa appears **erythematous, inflamed, and slightly thickened**.
2. **Possible Infiltration into Adjacent Tissues**:
    - The lesion appears to involve the **floor of the mouth, extending posteriorly**.
    - There is **no clear demarcation between the lesion and surrounding normal mucosa**, suggesting **invasive growth**.
3. **Poor Oral Hygiene and Presence of Dental Plaque and Calculus**:
    - Multiple **discolored, partially eroded teeth** suggest **long-standing poor oral hygiene**.
    - Chronic irritation from **dental factors may have contributed to mucosal changes**.

Most Probable Diagnosis: Oral Squamous Cell Carcinoma (OSCC)

- **Oral squamous cell carcinoma** is the **most common malignancy of the oral cavity**, frequently occurring on the **floor of the mouth, lateral tongue, and buccal mucosa**.
- **Risk factors**:
    - **Tobacco and alcohol use** (strongly associated).
    - **Poor oral hygiene and chronic irritation**.
    - **Human papillomavirus (HPV) infection** (less common in this location).
    - **History of leukoplakia or erythroplakia**.
- **Clinical features**:
    - **Ulcerated, irregular, and indurated mass**.
    - **Non-healing lesion >2 weeks**.
    - **Pain, dysphagia, speech difficulties, or referred ear pain (if advanced).**

Differential Diagnoses to Consider:

1. **Chronic Traumatic Ulcer (Unlikely Due to Infiltrative Appearance)**
    - May present with **erythema and ulceration** but lacks **exophytic growth and induration**.
    - Resolves once **the irritant is removed** (unlike OSCC).
2. **Tuberculosis-Associated Oral Ulcer (If Systemic Symptoms Are Present)**
    - Chronic **non-healing ulcer** with **necrotic sloughing**.
    - Often associated with **cervical lymphadenopathy and constitutional symptoms**.
3. **Syphilitic Ulcer (Chancre or Gumma)**
    - A **painless, non-healing ulcer** in primary syphilis.

- Secondary syphilis presents with **mucous patches**, while tertiary syphilis can cause **gummatous destruction**.

4. **Deep Fungal Infection (If Immunocompromised)**
    - **Histoplasmosis, blastomycosis, or paracoccidioidomycosis** may present as **chronic oral ulcers**.
    - More common in **HIV-positive or immunosuppressed patients**.
5. **Verrucous Carcinoma (If Growth Is Slow and Non-Ulcerated)**
    - A **slow-growing, exophytic, warty lesion**.
    - Unlike OSCC, it is **less invasive but still requires biopsy**.

Recommended Next Steps:

1. **Urgent Biopsy (Incisional)**
    - Histopathological examination to confirm **squamous cell carcinoma**.
    - Immunohistochemistry may be used for **differentiation if needed**.
2. **Imaging for Staging (CT/MRI of Head and Neck)**
    - Assess for **bone invasion, soft tissue extension, or lymph node involvement**.
    - PET scan if **distant metastasis is suspected**.
3. **Referral to a Head and Neck Oncology Team**
    - **Surgical resection with clear margins** if feasible.
    - **Adjuvant radiation and/or chemotherapy** based on TNM staging.
4. **Risk Factor Management**
    - **Tobacco and alcohol cessation**.
    - **Nutritional support** if oral intake is compromised.

Conclusion

This lesion is **highly suspicious for oral squamous cell carcinoma (OSCC)** and requires **urgent biopsy for confirmation**. **Advanced imaging and multidisciplinary oncologic evaluation are essential**. Would you like a **detailed protocol for biopsy and TNM staging?**

CASE 51

Reference:

https://cwc-react.s3.ap-south-1.amazonaws.com/img/About+Cancer/BM02-1.jpg

Chat-GPT4 answer:

Analysis and Probable Diagnosis

Observations:

1. **Extensive ulcerated and necrotic lesion on the posterior alveolar ridge and retromolar region**:
    - The lesion appears **irregular, ulcerative, and necrotic**, with **yellowish slough and fibrin deposition**.
    - **Surrounding mucosa is erythematous and inflamed**, indicating ongoing tissue destruction.
2. **Poor Oral Hygiene and Dental Condition**:
    - **Severe dental staining and calculus buildup**.
    - Adjacent teeth show **severe periodontal disease, possible mobility, and decay**.
3. **Bone Exposure and Possible Sequestration**:
    - The lesion seems to involve **underlying bone**, suggesting **osteonecrosis or deep tissue invasion**.
    - There are **features suggestive of necrotic bone or sequestration**, which could indicate **medication-related osteonecrosis of the jaw (MRONJ)** or **a necrotic malignancy**.

Most Probable Diagnosis: Medication-Related Osteonecrosis of the Jaw (MRONJ)

- **MRONJ** is strongly suspected given the **non-healing, ulcerative lesion with exposed bone**.
- Risk factors include:
    - **Use of bisphosphonates (e.g., alendronate, zoledronic acid) or denosumab**.
    - **History of chemotherapy or antiangiogenic therapy**.
    - **Prior dental extractions, trauma, or periodontal infection**.

Differential Diagnoses to Consider:

1. **Squamous Cell Carcinoma (SCC)**
    - The **necrotic ulceration, irregular borders, and involvement of adjacent tissue** could indicate **SCC of the alveolar ridge or retromolar area**.
    - SCC is common in **elderly patients, smokers, and those with poor oral hygiene**.
    - **Biopsy is mandatory** to rule out malignancy.
2. **Chronic Osteomyelitis**
    - Presents with **bone exposure, pain, swelling, and pus formation**.
    - Often **secondary to odontogenic infection or trauma**.
    - Unlike MRONJ, osteomyelitis usually **responds to antibiotics and surgical debridement**.

3. **Deep Fungal Infection (Mucormycosis, Aspergillosis)**
    - If the patient is **immunocompromised (e.g., diabetes, chemotherapy, corticosteroids use)**, fungal osteomyelitis should be considered.
    - More likely in cases with **black necrotic eschars and rapid tissue destruction**.
4. **Traumatic Ulcer with Superinfection**
    - Could be related to **chronic trauma from sharp teeth, dentures, or previous extraction sites**.
    - Typically heals within **2 weeks** unless superinfected or involving deeper structures.

Recommended Next Steps:

1. **Urgent Biopsy and Imaging**:
    - **Incisional biopsy** to differentiate **MRONJ from SCC**.
    - **CT or MRI** to assess **bone involvement, sequestration, or malignant infiltration**.
2. **Medication and Medical History Review**:
    - **Ask about bisphosphonate, denosumab, or chemotherapy use**.
    - **Assess systemic conditions (osteoporosis, cancer, multiple myeloma)**.
3. **Multidisciplinary Consultation**:
    - If MRONJ is confirmed, referral to **oral and maxillofacial surgery, oncology, or infectious disease specialists** may be needed.
    - If SCC is confirmed, an **oncology and head & neck surgery team should be involved**.
4. **Management Based on Diagnosis**:
    - **MRONJ**: Conservative management with **chlorhexidine rinses, antibiotics (if infected), and surgical debridement if indicated**.
    - **SCC**: **Surgical excision, neck dissection, and possible radiotherapy** depending on staging.
    - **Osteomyelitis**: **IV antibiotics and possible surgical debridement**.

Conclusion

This lesion is **highly suspicious for MRONJ or squamous cell carcinoma**, requiring **urgent biopsy and imaging** to confirm the diagnosis and plan appropriate treatment. **Would you like guidance on staging criteria, surgical management, or differential workup for MRONJ vs SCC?**

CASE 52

Reference:

https://dims.healthgrades.com/dims3/MMH/215c7e0/2147483647/strip/true/crop/1296x728+0+0/resize/1600x898!/quality/75/?url=https:%2F%2Fucmscdn.healthgrades.com%2F25%2Fd2%2Fdb601cd70b8af55291e90156b176%2Fhg-carcinoma-of-tongue.jpg

Chat-GPT4 answer:

Analysis and Probable Diagnosis

Observations:

1. **Ulcerative lesion on the lateral border of the tongue**:
   - The lesion presents as a **shallow ulcer with a mixed red and white appearance**.
   - There is **central necrosis or fibrin deposition**, giving it a **yellowish-gray aspect**.
   - The surrounding mucosa appears **erythematous and inflamed**.
2. **Irregular borders with apparent tissue breakdown**:
   - The ulcer shows **some degree of erosion and depth**.
   - There is **no clear exophytic mass**, but the lesion appears **chronic and non-healing**.
3. **No visible vesicles or satellite lesions**:
   - This suggests that the lesion is **not vesiculobullous in origin (e.g., herpes or pemphigus)**.

Most Probable Diagnosis: Traumatic Ulcer or Chronic Traumatic Ulcer (TUGSE - Traumatic Ulcerative Granuloma with Stromal Eosinophilia)

- **Chronic mechanical irritation (e.g., dental trauma, biting, sharp restorations)** can cause persistent ulcers, especially on the **lateral tongue**.
- **TUGSE** is a unique form of **traumatic ulcer** that can persist for weeks to months due to **deep inflammatory infiltration**, sometimes mimicking malignancy.
- **Self-healing if the source of trauma is removed**, but may require biopsy if it persists beyond 2–3 weeks.

Differential Diagnoses to Consider:

1. **Squamous Cell Carcinoma (If Persistent or Indurated)**
   - Key warning signs include:
     - **Firm, indurated base**.
     - **Irregular, raised borders**.
     - **Non-healing ulcer lasting >3 weeks**.
     - **Associated regional lymphadenopathy**.
   - If the ulcer does not heal, **biopsy is mandatory to rule out malignancy**.
2. **Major Aphthous Ulcer (If Recurrent)**
   - **Larger, painful, deep ulcers** lasting >2 weeks.
   - **No clear history of trauma**.

  - Typically seen in **Behçet's disease, inflammatory bowel disease, or immunosuppression**.
3. **Syphilitic Ulcer (Primary Chancre or Secondary Mucous Patch)**
   - Primary syphilitic **chancres are painless ulcers** with a **firm base**.
   - Secondary syphilis can present with **multiple mucous patches**.
   - Consider **serologic testing if risk factors are present**.
4. **Oral Tuberculosis (If Associated with Systemic Symptoms)**
   - Presents as a **chronic, non-healing ulcer**.
   - Associated with **night sweats, weight loss, chronic cough**.
   - Requires **biopsy and acid-fast bacillus staining**.
5. **Deep Fungal Infections (Histoplasmosis, Blastomycosis)**
   - If the patient is **immunocompromised or has traveled to endemic areas**, deep fungal infections should be considered.
   - **Biopsy with fungal stains and culture may be needed**.

Recommended Next Steps:

1. **Eliminate Any Local Trauma Sources**:
   - Assess for **sharp teeth, ill-fitting prostheses, or parafunctional habits (e.g., tongue thrusting, bruxism)**.
   - If present, modify or remove the source of trauma.
2. **Monitor for 2-3 Weeks**:
   - If the lesion heals after removing the trauma, no further intervention is needed.
   - If it persists **beyond 3 weeks or worsens, a biopsy is mandatory**.
3. **Biopsy Indications**:
   - **Non-healing ulcer lasting >2-3 weeks**.
   - **Induration, rolled borders, or exophytic growth**.
   - **Regional lymphadenopathy**.
   - **Patient history of smoking, alcohol use, or high-risk HPV exposure**.
4. **Consider Additional Tests if Clinically Indicated**:
   - **Serologic tests for syphilis or TB testing if systemic symptoms are present.**
   - **Fungal cultures if deep fungal infections are suspected**.

Conclusion

This lesion is **most likely a chronic traumatic ulcer** (possibly **TUGSE**). **First-line management is eliminating any source of mechanical trauma and observing for healing over 2-3 weeks**. If the lesion **persists, biopsy is strongly recommended** to rule out **oral squamous cell carcinoma or other chronic ulcerative conditions**. Would you like **a standardized biopsy protocol or a malignancy risk assessment guide for persistent oral ulcers?**

CASE 53

Reference:

https://www.researchgate.net/profile/Mark-Delacure/publication/225058197/figure/fig2/AS:646431320854528@1531132645129/Left-retromolar-squamous-cell-carcinoma-case-3_Q640.jpg

Chat-GPT4 answer:

Analysis and Probable Diagnosis

Observations:

1. **Ulcerated lesion with white and erythematous areas on the posterior alveolar ridge and retromolar region**:
    - The lesion appears **irregular, non-healing, and indurated**.
    - The central area shows **a mixed white and red pattern (erythroleukoplakia), with necrotic sloughing**.
    - The periphery has **an inflamed and slightly elevated border**, a concerning sign of **invasive growth**.
2. **High-Risk Location: Retromolar Trigone / Posterior Alveolar Ridge**:
    - The **retromolar trigone, soft palate, and posterior alveolar ridge** are high-risk sites for **oral squamous cell carcinoma (OSCC)**.
    - These areas often have **late-stage detection due to minimal early symptoms**.
3. **Ulceration and Induration – Hallmark Signs of Malignancy**:
    - A **non-healing ulcer with an indurated base** is highly suspicious for **OSCC**.
    - The **irregular and mixed color pattern** (white hyperkeratotic areas and red atrophic zones) increases the likelihood of **high-grade dysplasia or carcinoma**.

Most Probable Diagnosis: Oral Squamous Cell Carcinoma (OSCC)

- The lesion exhibits **hallmark features of OSCC**, including:
    - **Non-healing ulceration**
    - **Mixed leukoplakic and erythroplakic components**
    - **Induration with irregular borders**
    - **Posterior oral cavity involvement (retromolar trigone, alveolar ridge)**
- OSCC is the **most common oral malignancy**, often associated with:
    - **Tobacco and alcohol use**
    - **Human papillomavirus (HPV) infection (especially HPV-16)**
    - **Chronic irritation from ill-fitting prostheses, dental trauma**

Differential Diagnoses to Consider:

1. **Severe Epithelial Dysplasia / Carcinoma in Situ**
    - If **no invasion is detected histologically**, the lesion may represent a **precancerous state**.
    - **Requires excision due to high malignant potential**.

2. **Chronic Traumatic Ulcer (If Associated with Mechanical Trauma)**
    - Often seen in **denture wearers or near sharp teeth**.
    - Unlike OSCC, **traumatic ulcers heal within 2-3 weeks after irritant removal**.
    - A **biopsy is mandatory if non-healing or indurated**.
3. **Verrucous Carcinoma (If Slow-Growing and Exophytic)**
    - A **low-grade SCC variant**, often presenting as a **warty, slow-growing lesion**.
    - **Less likely** but requires **biopsy for confirmation**.
4. **Deep Fungal Infection (If Immunosuppressed)**
    - **Histoplasmosis, blastomycosis, or paracoccidioidomycosis** can present as **chronic, indurated oral ulcers**.
    - More common in **immunocompromised individuals**.
    - A **fungal culture and PAS stain on biopsy** can confirm the diagnosis.

Urgent Recommended Next Steps:

1. **Immediate Incisional Biopsy**:
    - **Histopathological confirmation is critical** to assess for **invasive squamous cell carcinoma**.
    - **Immunohistochemistry (p16, CK5/6, p63)** may help classify the lesion (HPV-related vs. conventional SCC).
2. **Imaging Studies (CT/MRI) for Local Invasion**:
    - Evaluate **bone involvement, perineural spread, and lymph node metastasis**.
3. **TNM Staging and Multidisciplinary Management**:
    - If SCC is confirmed, **staging determines treatment strategy**.
    - Possible treatments include:
        - **Surgical resection with clear margins (if localized)**
        - **Neck dissection (if lymph node involvement)**
        - **Radiotherapy or chemoradiotherapy (for advanced cases)**

Conclusion

This lesion is **highly suspicious for oral squamous cell carcinoma (OSCC)** and requires **urgent biopsy and imaging** for definitive diagnosis and staging. Early diagnosis is **critical for prognosis**, and immediate referral to **oral surgery, head and neck oncology, or maxillofacial surgery is advised**. Would you like assistance with **TNM staging, treatment guidelines, or surgical approaches for OSCC?**

CASE 54

Reference:

https://th.bing.com/th/id/R.76e87f1cf301a7059656707ef0e2ed30?rik=etlmg8vFY922tQ&riu=http%3a%2f%2fkindaipicks.com%2fuploads%2f201902%2f7c34380fc570b09fdd3c963279aeab72f1efad81.jpg&ehk=Gu%2bcgYVWrt4WKFJqzWlucvRQEMJub8DSwhd9DvlSL6w%3d&risl=&pid=ImgRaw&r=0

Chat-GPT4 answer:

Analysis and Probable Diagnosis

Observations:

1. **Ulcerated, irregular lesion on the lateral tongue and floor of the mouth**:
   - The lesion is **non-homogeneous**, with **white hyperkeratotic areas and surrounding erythema**.
   - It has **an irregular, raised, and indurated border**, a key sign of **malignant transformation**.
   - The surface appears **rough, possibly verrucous**, and non-wipeable.
2. **Surrounding Tissue Shows Erythema and Trauma-Like Changes**:
   - The adjacent **inflamed mucosa** could indicate **chronic irritation or secondary infection**.
   - No apparent **pus or necrotic debris**, suggesting **this is not a simple traumatic ulcer**.
3. **High-Risk Location: Lateral Border of the Tongue and Floor of the Mouth**:
   - The **lateral tongue and floor of the mouth** are among the **highest-risk sites for malignant transformation**.
   - These areas are frequently affected by **chronic irritation (e.g., dental trauma, tobacco, alcohol use).**

Most Probable Diagnosis: Oral Squamous Cell Carcinoma (OSCC)

- The lesion exhibits **hallmark features of OSCC**, including:
  - **Non-healing ulceration**
  - **Irregular keratotic thickening**
  - **Erythroplakic changes, which have a high malignant transformation rate**
  - **Lateral tongue involvement, a high-risk anatomical site**
- OSCC is the **most common oral malignancy**, often associated with:
  - **Tobacco and alcohol use**
  - **Human papillomavirus (HPV) infection (especially HPV-16)**
  - **Chronic irritation (e.g., ill-fitting prostheses, sharp teeth)**

Differential Diagnoses to Consider:

1. **Severe Epithelial Dysplasia / Carcinoma in Situ (If No Invasion on Biopsy)**
   - If **no invasion is detected histologically**, the lesion may represent a **precancerous state**.

    - **Requires excision due to high malignant potential**.
2. **Chronic Traumatic Ulcer (If Associated with Mechanical Trauma)**
    - Often seen in **denture wearers or near sharp teeth**.
    - Unlike OSCC, **traumatic ulcers heal within 2-3 weeks after irritant removal**.
    - A **biopsy is mandatory if non-healing or indurated**.
3. **Verrucous Carcinoma (If Slow-Growing and Exophytic)**
    - A **low-grade SCC variant**, often presenting as a **warty, slow-growing lesion**.
    - **Less likely** but requires **biopsy for confirmation**.
4. **Deep Fungal Infection (If Immunosuppressed)**
    - **Histoplasmosis, blastomycosis, or paracoccidioidomycosis** can present as **chronic, indurated oral ulcers**.
    - More common in **immunocompromised individuals**.
    - A **fungal culture and PAS stain on biopsy** can confirm the diagnosis.

Urgent Recommended Next Steps:

1. **Immediate Incisional Biopsy**:
    - **Histopathological confirmation is critical** to assess for **invasive squamous cell carcinoma**.
    - **Immunohistochemistry (p16, CK5/6, p63)** may help classify the lesion (HPV-related vs. conventional SCC).
2. **Imaging Studies (CT/MRI) for Local Invasion**:
    - Evaluate **bone involvement, perineural spread, and lymph node metastasis**.
3. **TNM Staging and Multidisciplinary Management**:
    - If SCC is confirmed, **staging determines treatment strategy**.
    - Possible treatments include:
        - **Surgical resection with clear margins (if localized)**
        - **Neck dissection (if lymph node involvement)**
        - **Radiotherapy or chemoradiotherapy (for advanced cases)**

Conclusion

This lesion is **highly suspicious for oral squamous cell carcinoma (OSCC)** and requires **urgent biopsy and imaging** for definitive diagnosis and staging. Early diagnosis is **critical for prognosis**, and immediate referral to **oral surgery, head and neck oncology, or maxillofacial surgery is advised**. Would you like assistance with **TNM staging, treatment guidelines, or surgical approaches for OSCC?**

CASE 55

Reference:

https://upload.wikimedia.org/wikipedia/commons/thumb/4/47/PLoS_oral_cancer.png/360px-PLoS_oral_cancer.png

Chat-GPT4 answer:

Analysis and Probable Diagnosis

Observations:

1. **Large, necrotic, ulceroproliferative lesion on the dorsal and lateral tongue**:
    - **Irregular, fungating, and ulcerated mass** with mixed **erythematous, keratotic, and necrotic** areas.
    - A large area of **necrosis with a yellowish-black eschar**.
    - **Surrounding leukoplakic and erythroplakic changes**, suggesting **field cancerization**.
2. **Extensive Surface Keratinization and Erosions**:
    - **Irregular thickened white plaques** interspersed with **ulceration and erythema**.
    - The surface appears **rough, exophytic, and infiltrative**, raising suspicion for an aggressive pathology.
3. **High-Risk Features**:
    - **Lateral tongue involvement**, a **high-risk site for oral malignancies**.
    - **Infiltrative growth, necrosis, and possible induration**, suggesting **deep invasion**.
    - **Advanced stage lesion with possible muscle involvement**.

Most Probable Diagnosis: Advanced Squamous Cell Carcinoma (SCC) of the Tongue

- **Oral squamous cell carcinoma (OSCC)** is the **most common oral malignancy**, especially in high-risk sites like the **lateral and ventral tongue**.
- The presence of:
    - **Ulceration**
    - **Irregular proliferative growth**
    - **Mixed leukoplakic and erythroplakic areas**
    - **Necrosis and deep infiltration** strongly suggests **advanced-stage OSCC**.

Differential Diagnoses to Consider:

1. **Verrucous Carcinoma (If Slow-Growing, Less Invasive)**
    - A **low-grade variant of SCC**, often associated with **chronic tobacco use**.
    - Presents as a **slow-growing, exophytic, cauliflower-like lesion**.
    - **Less tendency for deep invasion** compared to SCC.
2. **Chronic Traumatic Ulcer (Less Likely Given the Mass-Like Growth)**
    - Would have a **clear history of repeated trauma (sharp teeth, dentures, or tongue biting)**.
    - Typically **smaller, less infiltrative, and resolves after removal of the trauma**.
    - **Biopsy is mandatory for ulcers persisting >2 weeks**.

3. **Necrotizing Sialometaplasia (If Self-Limiting and Non-Indurated)**
   - A **benign, ischemic lesion of minor salivary glands**.
   - Mimics malignancy but **resolves spontaneously within weeks**.
   - **Biopsy is essential to rule out carcinoma**.
4. **Tuberculous Ulcer (If Associated with Pulmonary TB)**
   - Presents as a **chronic, non-healing ulcer with undermined edges**.
   - Requires **histopathological confirmation of granulomas and acid-fast bacilli (AFB staining)**.

Recommended Next Steps:

1. **Urgent Incisional Biopsy**
   - **Histopathological confirmation is critical** for staging and treatment planning.
   - If SCC is diagnosed, **immunohistochemistry (p16, CK5/6, p63) can help differentiate subtypes**.
2. **Imaging Studies (Essential for Staging)**
   - **Contrast-enhanced MRI or CT scan** to assess:
     - **Tumor size and depth of invasion**.
     - **Involvement of deep structures (muscle, bone, nerves)**.
   - **PET-CT scan** if SCC is confirmed to evaluate **lymph node and distant metastasis**.
3. **TNM Staging (AJCC Staging System)**
   - Based on:
     - **Tumor size (T)**
     - **Nodal involvement (N)**
     - **Distant metastasis (M)**
   - If lymph nodes are involved, a **neck dissection** may be necessary.
4. **Multidisciplinary Treatment Planning**
   - Referral to a **Head and Neck Oncologic Team** for staging and treatment decisions.
   - Treatment is likely to include:
     - **Surgical resection (partial or total glossectomy)** depending on tumor extent.
     - **Neck dissection (if nodal involvement is detected)**.
     - **Adjuvant radiotherapy and/or chemotherapy** based on stage and histopathology.

Conclusion

This lesion is **highly suspicious for advanced squamous cell carcinoma (SCC) of the tongue**, requiring **urgent biopsy and imaging** to confirm the diagnosis and stage the disease. **Immediate referral to an oncology center is warranted for multidisciplinary management**. Would you like **detailed TNM staging criteria or treatment protocols for advanced OSCC?**

CASE 56

Reference:

https://www.researchgate.net/publication/325996651/figure/fig3/AS:643358636658694@1530400060135/a-Oral-squamous-cell-carcinoma-in-the-floor-of-the-mouth-conventional-white-light.png

Chat-GPT4 answer:

Analysis and Probable Diagnosis

Observations:

1. **Exophytic, ulcerated, and irregular mass on the floor of the mouth**:
   - The lesion appears **erythematous, irregular, and ulcerated with a verrucous or papillary surface**.
   - The mucosa around the lesion is **inflamed and thickened**, suggesting **chronic irritation or infiltration**.
2. **Possible Involvement of the Surrounding Soft Tissues**:
   - The lesion seems to extend **into the floor of the mouth**.
   - The borders are **ill-defined, with areas of necrosis**.
3. **Severe Dental Calculus and Poor Oral Hygiene**:
   - There is **extensive dental plaque, calculus, and periodontal disease**.
   - Chronic **dental and mucosal irritation** may have contributed to the development of the lesion.

Most Probable Diagnosis: Verrucous Carcinoma or Oral Squamous Cell Carcinoma (OSCC)

- **Verrucous carcinoma (VC)** is a **low-grade variant of squamous cell carcinoma** that presents as a **slow-growing, exophytic, and warty lesion**.
- It is **locally aggressive but has a low metastatic potential**.
- **Risk factors** include:
  - **Tobacco (smoking and smokeless), chronic irritation, and poor oral hygiene**.
  - **Human papillomavirus (HPV) association** in some cases.
- **Oral Squamous Cell Carcinoma (OSCC)** must be considered, given the **ulceration, irregular surface, and potential infiltration**.
  - OSCC is **highly invasive** and has a **higher risk of metastasis**.
  - The presence of **necrosis and mucosal destruction** is more suggestive of OSCC rather than VC.

Differential Diagnoses to Consider:

1. **Proliferative Verrucous Leukoplakia (If Multiple Sites Are Affected)**
   - **Slowly progressing, multifocal white patches** that may evolve into carcinoma.
   - Has a **high malignant transformation rate**.
2. **Chronic Traumatic Ulcer (If Related to Mechanical Irritation)**
   - Could be caused by **sharp teeth, prostheses, or constant mechanical trauma**.

- Unlike OSCC, it should **heal after removing the irritant**.

3. **Deep Fungal Infection (If Immunocompromised)**
    - **Histoplasmosis or blastomycosis** can present as **chronic oral ulcerations**.
    - Typically associated with **systemic symptoms**.
4. **Tuberculosis-Associated Oral Ulcer (If Systemic Symptoms Are Present)**
    - Chronic **non-healing ulcer** with **caseating necrosis**.
    - Associated with **cervical lymphadenopathy**.

Recommended Next Steps:

1. **Urgent Biopsy (Incisional)**
    - Histopathological examination to differentiate **verrucous carcinoma, OSCC, or another etiology**.
    - If verrucous carcinoma is suspected, **deep incisional biopsy** is preferred.
2. **Imaging for Staging (CT/MRI of Head and Neck)**
    - Assess for **soft tissue and bone invasion**.
    - If OSCC is confirmed, **PET-CT for metastatic workup** is necessary.
3. **Referral to an Oncology Team**
    - **Wide surgical excision with clear margins** is the primary treatment.
    - **Adjuvant radiation therapy** if high-risk features are present.
4. **Risk Factor Management**
    - **Tobacco cessation and oral hygiene improvement**.
    - **Nutritional support** if oral function is impaired.

Conclusion

This lesion is **highly suspicious for verrucous carcinoma or oral squamous cell carcinoma**, requiring **urgent biopsy and oncologic evaluation**. **Imaging studies should be performed to assess invasion and metastasis**. Would you like **a detailed guide on biopsy protocols and TNM staging for OSCC?**

CASE 57

Reference:

https://www.lsusd.lsuhsc.edu/socs/images/cancer%202.jpg

Chat-GPT4 answer:

Analysis and Probable Diagnosis

Observations:

1. **Exophytic, irregular, and ulcerated mass in the maxillary posterior region**:
   - The lesion appears **proliferative, lobulated, and erythematous**.
   - The surface shows **areas of necrosis, ulceration, and fibrin deposition**.
   - The borders appear **irregular and infiltrative**.
2. **Involvement of the Hard Palate and Alveolar Ridge**:
   - The lesion seems to extend **into the posterior maxillary alveolar mucosa and palate**.
   - Possible **bone destruction** or **soft tissue infiltration**.
3. **Absence of Clear Signs of Infection or Benign Inflammatory Conditions**:
   - No signs of **pseudomembranous exudates** (ruling out candidiasis).
   - The lesion is **not well-demarcated**, making **benign hyperplasia or reactive lesions less likely**.

Most Probable Diagnosis: Squamous Cell Carcinoma (SCC) of the Maxilla

- **Oral squamous cell carcinoma (OSCC)** is the most common malignancy affecting the **oral mucosa**, especially in high-risk sites such as:
  - **Lateral tongue**
  - **Floor of the mouth**
  - **Retromolar and maxillary alveolar ridge**
- The **ulcerated, exophytic, and infiltrative nature** of this lesion is highly suspicious for SCC.
- **Risk factors** include:
  - **Tobacco and alcohol use**.
  - **HPV-related carcinogenesis** (especially for oropharyngeal SCC).
  - **Poor oral hygiene and chronic irritation**.
  - **Betel quid chewing** (common in certain populations).

Differential Diagnoses to Consider:

1. **Verrucous Carcinoma (If a Warty Growth is Present)**
   - A **low-grade variant of SCC**, which appears as a **slow-growing, wart-like, exophytic mass**.
   - Typically, verrucous carcinoma **does not ulcerate early**.
2. **Osteonecrosis of the Jaw (If History of Bisphosphonate or Denosumab Use)**
   - If the patient has a history of **antiresorptive or antiangiogenic therapy**, this could represent **medication-related osteonecrosis of the jaw (MRONJ)**.

- MRONJ presents as **exposed necrotic bone, secondary infection, and inflammatory response**.

3. **Deep Fungal Infection (Histoplasmosis, Blastomycosis)**
    - Fungal infections, particularly in **immunocompromised individuals**, can mimic OSCC.
    - Would require **biopsy and fungal stains (PAS, GMS)** for confirmation.
4. **Tuberculous Ulcer (If History of Systemic Symptoms)**
    - Oral tuberculosis can present as a **chronic, non-healing ulcer or mass**.
    - Consider **TB testing if the patient has systemic signs such as weight loss, night sweats, or chronic cough**.
5. **Syphilitic Gumma (If Risk Factors for Syphilis Exist)**
    - Tertiary syphilis can cause **granulomatous lesions with necrosis**, often mistaken for malignancy.
    - **Serologic testing (VDRL, FTA-ABS) is necessary for confirmation**.

Recommended Next Steps:

1. **Urgent Incisional Biopsy**
    - **Histopathologic confirmation** is necessary to distinguish between SCC, verrucous carcinoma, or another etiology.
    - **Immunohistochemical staining (p16 for HPV, CK5/6 for SCC) can aid diagnosis**.
2. **Imaging (CBCT, CT, or MRI)**
    - Assess for **bony invasion** or **extension to adjacent structures**.
    - MRI is particularly useful for **soft tissue spread**.
3. **Assessment of Regional Lymph Nodes**
    - Palpate for **cervical lymphadenopathy** (suggestive of metastasis).
    - If SCC is confirmed, a **neck ultrasound or CT scan is required** for staging.
4. **Oncology Referral for Management Planning**
    - If SCC is confirmed, treatment typically involves:
        - **Surgical excision with clear margins**.
        - **Neck dissection if nodal involvement is present**.
        - **Adjuvant radiotherapy ± chemotherapy based on staging**.
5. **Comprehensive Risk Assessment**
    - **Smoking cessation counseling** if applicable.
    - **HPV testing if risk factors suggest viral oncogenesis**.

Conclusion

This lesion is **highly suspicious for squamous cell carcinoma (SCC) of the maxilla**, given its **exophytic, ulcerated, and infiltrative characteristics. Urgent biopsy and imaging are mandatory for diagnosis and staging**. Would you like **a detailed SCC staging guide or an oncologic management protocol?**

CASE 58

Reference:

https://www.medigraphic.com/files/adm/od-2023/6/od236i-2.jpg

Chat-GPT4 answer:

Analysis and Probable Diagnosis

Observations:

1. **Exophytic, irregular, and ulcerated mass in the maxillary posterior region**:
   - The lesion appears **proliferative, lobulated, and erythematous**.
   - The surface shows **areas of necrosis, ulceration, and fibrin deposition**.
   - The borders appear **irregular and infiltrative**.
2. **Involvement of the Hard Palate and Alveolar Ridge**:
   - The lesion seems to extend **into the posterior maxillary alveolar mucosa and palate**.
   - Possible **bone destruction** or **soft tissue infiltration**.
3. **Absence of Clear Signs of Infection or Benign Inflammatory Conditions**:
   - No signs of **pseudomembranous exudates** (ruling out candidiasis).
   - The lesion is **not well-demarcated**, making **benign hyperplasia or reactive lesions less likely**.

Most Probable Diagnosis: Squamous Cell Carcinoma (SCC) of the Maxilla

- **Oral squamous cell carcinoma (OSCC)** is the most common malignancy affecting the **oral mucosa**, especially in high-risk sites such as:
  - **Lateral tongue**
  - **Floor of the mouth**
  - **Retromolar and maxillary alveolar ridge**
- The **ulcerated, exophytic, and infiltrative nature** of this lesion is highly suspicious for SCC.
- **Risk factors** include:
  - **Tobacco and alcohol use**.
  - **HPV-related carcinogenesis** (especially for oropharyngeal SCC).
  - **Poor oral hygiene and chronic irritation**.
  - **Betel quid chewing** (common in certain populations).

Differential Diagnoses to Consider:

1. **Verrucous Carcinoma (If a Warty Growth is Present)**
   - A **low-grade variant of SCC**, which appears as a **slow-growing, wart-like, exophytic mass**.
   - Typically, verrucous carcinoma **does not ulcerate early**.
2. **Osteonecrosis of the Jaw (If History of Bisphosphonate or Denosumab Use)**
   - If the patient has a history of **antiresorptive or antiangiogenic therapy**, this could represent **medication-related osteonecrosis of the jaw (MRONJ)**.

- MRONJ presents as **exposed necrotic bone, secondary infection, and inflammatory response**.

3. **Deep Fungal Infection (Histoplasmosis, Blastomycosis)**
   - Fungal infections, particularly in **immunocompromised individuals**, can mimic OSCC.
   - Would require **biopsy and fungal stains (PAS, GMS)** for confirmation.
4. **Tuberculous Ulcer (If History of Systemic Symptoms)**
   - Oral tuberculosis can present as a **chronic, non-healing ulcer or mass**.
   - Consider **TB testing if the patient has systemic signs such as weight loss, night sweats, or chronic cough**.
5. **Syphilitic Gumma (If Risk Factors for Syphilis Exist)**
   - Tertiary syphilis can cause **granulomatous lesions with necrosis**, often mistaken for malignancy.
   - **Serologic testing (VDRL, FTA-ABS) is necessary for confirmation**.

Recommended Next Steps:

1. **Urgent Incisional Biopsy**
   - **Histopathologic confirmation** is necessary to distinguish between SCC, verrucous carcinoma, or another etiology.
   - **Immunohistochemical staining (p16 for HPV, CK5/6 for SCC) can aid diagnosis**.
2. **Imaging (CBCT, CT, or MRI)**
   - Assess for **bony invasion** or **extension to adjacent structures**.
   - MRI is particularly useful for **soft tissue spread**.
3. **Assessment of Regional Lymph Nodes**
   - Palpate for **cervical lymphadenopathy** (suggestive of metastasis).
   - If SCC is confirmed, a **neck ultrasound or CT scan is required** for staging.
4. **Oncology Referral for Management Planning**
   - If SCC is confirmed, treatment typically involves:
     - **Surgical excision with clear margins**.
     - **Neck dissection if nodal involvement is present**.
     - **Adjuvant radiotherapy ± chemotherapy based on staging**.
5. **Comprehensive Risk Assessment**
   - **Smoking cessation counseling** if applicable.
   - **HPV testing if risk factors suggest viral oncogenesis**.

Conclusion

This lesion is **highly suspicious for squamous cell carcinoma (SCC) of the maxilla**, given its **exophytic, ulcerated, and infiltrative characteristics. Urgent biopsy and imaging are mandatory for diagnosis and staging**. Would you like **a detailed SCC staging guide or an oncologic management protocol?**

<u>CASE 59</u>

<u>Reference:</u>
https://cdn.lecturio.com/assets/Oral-carcinoma-with-exophytic-tongue-masses.jpg

<u>Chat-GPT4 answer:</u>

Analysis and Probable Diagnosis

Observations:

1. **Large, necrotic, ulceroproliferative lesion on the dorsal and lateral tongue**:
   - **Irregular, fungating, and ulcerated mass** with mixed **erythematous, keratotic, and necrotic** areas.
   - A large area of **necrosis with a yellowish-black eschar**.
   - **Surrounding leukoplakic and erythroplakic changes**, suggesting **field cancerization**.
2. **Extensive Surface Keratinization and Erosions**:
   - **Irregular thickened white plaques** interspersed with **ulceration and erythema**.
   - The surface appears **rough, exophytic, and infiltrative**, raising suspicion for an aggressive pathology.
3. **High-Risk Features**:
   - **Lateral tongue involvement**, a **high-risk site for oral malignancies**.
   - **Infiltrative growth, necrosis, and possible induration**, suggesting **deep invasion**.
   - **Advanced stage lesion with possible muscle involvement**.

Most Probable Diagnosis: Advanced Squamous Cell Carcinoma (SCC) of the Tongue

- **Oral squamous cell carcinoma (OSCC)** is the **most common oral malignancy**, especially in high-risk sites like the **lateral and ventral tongue**.
- The presence of:
  - **Ulceration**
  - **Irregular proliferative growth**
  - **Mixed leukoplakic and erythroplakic areas**
  - **Necrosis and deep infiltration** strongly suggests **advanced-stage OSCC**.

Differential Diagnoses to Consider:

1. **Verrucous Carcinoma (If Slow-Growing, Less Invasive)**
   - A **low-grade variant of SCC**, often associated with **chronic tobacco use**.
   - Presents as a **slow-growing, exophytic, cauliflower-like lesion**.
   - **Less tendency for deep invasion** compared to SCC.
2. **Chronic Traumatic Ulcer (Less Likely Given the Mass-Like Growth)**
   - Would have a **clear history of repeated trauma (sharp teeth, dentures, or tongue biting)**.
   - Typically **smaller, less infiltrative, and resolves after removal of the trauma**.
   - **Biopsy is mandatory for ulcers persisting >2 weeks**.
3. **Necrotizing Sialometaplasia (If Self-Limiting and Non-Indurated)**
   - A **benign, ischemic lesion of minor salivary glands**.
   - Mimics malignancy but **resolves spontaneously within weeks**.

    - **Biopsy is essential to rule out carcinoma**.
4. **Tuberculous Ulcer (If Associated with Pulmonary TB)**
    - Presents as a **chronic, non-healing ulcer with undermined edges**.
    - Requires **histopathological confirmation of granulomas and acid-fast bacilli (AFB staining)**.

Recommended Next Steps:

1. **Urgent Incisional Biopsy**
    - **Histopathological confirmation is critical** for staging and treatment planning.
    - If SCC is diagnosed, **immunohistochemistry (p16, CK5/6, p63) can help differentiate subtypes**.
2. **Imaging Studies (Essential for Staging)**
    - **Contrast-enhanced MRI or CT scan** to assess:
        - **Tumor size and depth of invasion**.
        - **Involvement of deep structures (muscle, bone, nerves)**.
    - **PET-CT scan** if SCC is confirmed to evaluate **lymph node and distant metastasis**.
3. **TNM Staging (AJCC Staging System)**
    - Based on:
        - **Tumor size (T)**
        - **Nodal involvement (N)**
        - **Distant metastasis (M)**
    - If lymph nodes are involved, a **neck dissection** may be necessary.
4. **Multidisciplinary Treatment Planning**
    - Referral to a **Head and Neck Oncologic Team** for staging and treatment decisions.
    - Treatment is likely to include:
        - **Surgical resection (partial or total glossectomy)** depending on tumor extent.
        - **Neck dissection (if nodal involvement is detected)**.
        - **Adjuvant radiotherapy and/or chemotherapy** based on stage and histopathology.

Conclusion

This lesion is **highly suspicious for advanced squamous cell carcinoma (SCC) of the tongue**, requiring **urgent biopsy and imaging** to confirm the diagnosis and stage the disease. **Immediate referral to an oncology center is warranted for multidisciplinary management**. Would you like **detailed TNM staging criteria or treatment protocols for advanced OSCC?**

CASE 60

Reference:

https://media.springernature.com/lw685/springer-static/image/chp%3A10.1007%2F978-981-15-1346-6_81/MediaObjects/462947_1_En_81_Fig13_HTML.jpg

Chat-GPT4 answer:

Analysis and Probable Diagnosis

Observations:

1. **Exophytic, irregular, and ulcerated mass on the lateral tongue and floor of the mouth**:
    - The lesion is **erythematous with areas of white, necrotic sloughing**.
    - The surface appears **irregular, friable, and infiltrative**.
    - The lesion is **indurated**, suggesting **deep tissue invasion**.
2. **Possible Involvement of the Adjacent Soft Tissues**:
    - The lesion extends **along the lateral border of the tongue and the floor of the mouth**.
    - The surrounding mucosa appears **erythematous and edematous**.
3. **Poor Oral Hygiene and Presence of Dental Plaque and Calculus**:
    - The patient has **severe periodontal disease, with heavy calculus deposits and poor oral hygiene**.
    - Chronic irritation from **dental factors could contribute to persistent mucosal injury**.

Most Probable Diagnosis: Oral Squamous Cell Carcinoma (OSCC)

- **Oral squamous cell carcinoma (OSCC)** is the **most common malignancy of the oral cavity**, frequently occurring on the **lateral tongue, floor of the mouth, and buccal mucosa**.
- **Risk factors**:
    - **Tobacco and alcohol use** (strongest association).
    - **Poor oral hygiene and chronic irritation**.
    - **Human papillomavirus (HPV) infection** (less common in this location).
    - **History of leukoplakia or erythroplakia**.
- **Clinical features**:
    - **Non-healing ulcer >2 weeks**.
    - **Exophytic growth with irregular borders**.
    - **Pain, dysphagia, or referred ear pain (if advanced).**

Differential Diagnoses to Consider:

1. **Verrucous Carcinoma (If Growth is Slow and Non-Ulcerated)**
    - A **slow-growing, warty lesion** that may resemble OSCC but lacks ulceration initially.
    - Has **low metastatic potential but requires excision**.
2. **Chronic Traumatic Ulcer (If Related to Mechanical Irritation)**
    - Caused by **sharp teeth, prostheses, or constant mechanical trauma**.
    - Unlike OSCC, it should **heal within 2-3 weeks after removing the irritant**.

3. **Deep Fungal Infection (If Immunocompromised)**
    - **Histoplasmosis or blastomycosis** can present as **chronic oral ulcerations**.
    - More common in **HIV-positive or immunosuppressed patients**.
4. **Tuberculosis-Associated Oral Ulcer (If Systemic Symptoms Are Present)**
    - Chronic **non-healing ulcer** with **caseating necrosis**.
    - Associated with **cervical lymphadenopathy**.

Recommended Next Steps:

1. **Urgent Biopsy (Incisional)**
    - Histopathological examination to confirm **OSCC**.
    - Immunohistochemistry may be used for **differentiation if needed**.
2. **Imaging for Staging (CT/MRI of Head and Neck)**
    - Assess for **soft tissue, bone invasion, or lymph node involvement**.
    - If OSCC is confirmed, **PET-CT for metastatic workup** is necessary.
3. **Referral to a Head and Neck Oncology Team**
    - **Wide surgical excision with clear margins** if feasible.
    - **Adjuvant radiation and/or chemotherapy** based on TNM staging.
4. **Risk Factor Management**
    - **Tobacco and alcohol cessation**.
    - **Nutritional support** if oral intake is compromised.

Conclusion

This lesion is **highly suspicious for oral squamous cell carcinoma (OSCC)** and requires **urgent biopsy for confirmation**. **Advanced imaging and multidisciplinary oncologic evaluation are essential**. Would you like a **detailed protocol for biopsy and TNM staging?**

CASE 61

Reference:

https://www.dentaljuku.net/wp-content/uploads/2016/12/8ec6a9c9f17f674d2b5aa83ddff080e1.jpg

Chat-GPT4 answer:

Analysis and Probable Diagnosis

Observations:

1. **Extensive, irregular ulcerated lesion on the lateral tongue**:
   - The lesion presents as a **large, non-homogeneous area with a mixture of ulceration, keratosis, and erythematous regions**.
   - The surface appears **indurated, verrucous, and necrotic in some areas**.
   - The lesion is **non-wipeable**, indicating a deep epithelial or submucosal process.
2. **High-Risk Site: Lateral Border of the Tongue**:
   - The **lateral tongue is one of the most common locations for oral squamous cell carcinoma (OSCC)**.
   - This site is **prone to chronic irritation from dental restorations, occlusal trauma, tobacco, and alcohol**.
3. **Irregular Borders and Non-Healing Ulceration**:
   - The lesion demonstrates **hallmark malignant features**, including:
     - **Irregular, infiltrative borders**
     - **Persistent ulceration with necrosis**
     - **Mixed white (keratotic) and red (erythroplakic) components**
     - **Possible perilesional induration**.

Most Probable Diagnosis: Oral Squamous Cell Carcinoma (OSCC)

- The lesion exhibits **key characteristics of OSCC**, including:
  - **Non-healing ulceration**
  - **Induration and irregular margins**
  - **Mixed leukoplakic and erythroplakic patterns**
  - **Lateral tongue involvement, a high-risk site for malignancy**.
- OSCC accounts for **over 90% of oral malignancies**, strongly linked to:
  - **Tobacco and alcohol consumption**
  - **Human papillomavirus (HPV-16)**
  - **Chronic irritation or mechanical trauma**.

Differential Diagnoses to Consider:

1. **Verrucous Carcinoma (If Slow-Growing and Exophytic)**
   - A **low-grade SCC variant**, presenting as a **warty, slow-growing lesion**.
   - Requires **biopsy for histological differentiation**.
2. **Severe Epithelial Dysplasia / Carcinoma in Situ (If No Invasion on Biopsy)**

    - If **no invasion is detected histologically**, the lesion may represent a **precancerous state**.
    - **Requires excision due to high malignant potential**.
3. **Chronic Hyperplastic Candidiasis (Less Likely but Possible Superimposed Infection)**
    - Persistent **white plaque that does not wipe off**, sometimes associated with **Candida overgrowth**.
    - **PAS stain on biopsy** can confirm **Candida superinfection**, but it would not explain the induration.
4. **Traumatic Ulcerative Granuloma (If No Malignant Features on Biopsy)**
    - Chronic ulcers with **deep tissue inflammation**.
    - Differentiation from SCC requires **biopsy with histopathologic examination**.

Urgent Recommended Next Steps:

1. **Immediate Incisional Biopsy**:
    - **Histopathological confirmation is essential** to assess for **invasive squamous cell carcinoma**.
    - **Immunohistochemistry (p16, CK5/6, p63) may help differentiate HPV-associated SCC from conventional SCC**.
2. **Imaging Studies (CT/MRI) for Local Invasion**:
    - Evaluate **bone involvement, perineural spread, and lymph node metastasis**.
3. **TNM Staging and Multidisciplinary Management**:
    - If SCC is confirmed, **staging determines treatment strategy**.
    - Possible treatments include:
        - **Surgical resection with clear margins (if localized)**
        - **Neck dissection (if lymph node involvement)**
        - **Radiotherapy or chemoradiotherapy (for advanced cases)**.

Conclusion

This lesion is **highly suspicious for oral squamous cell carcinoma (OSCC)** and requires **urgent biopsy and imaging** for definitive diagnosis and staging. Early diagnosis is **critical for prognosis**, and immediate referral to **oral surgery, head and neck oncology, or maxillofacial surgery is advised**. Would you like assistance with **TNM staging, treatment guidelines, or surgical approaches for OSCC?**

CASE 62

Reference:

https://postimg.cc/jnQfdsgM

Chat-GPT4 answer:

Analysis and Probable Diagnosis

Observations:

1. **Exophytic, Ulcerated, and Indurated Lesion on the Dorsal Tongue:**
   - The lesion presents as an **irregular, proliferative mass with ulceration and induration**.
   - The surface exhibits **an irregular, verrucous texture with areas of erosion and leukoplakia**.
   - These features raise a strong suspicion of **a malignant process, likely squamous cell carcinoma (SCC)**.
2. **Localization on the Mid-Dorsal Tongue:**
   - While SCC is most common on the **lateral and ventral tongue**, the dorsal tongue can also be affected.
   - The lesion's **non-healing nature, ulceration, and infiltrative characteristics** make malignancy highly probable.
3. **Leukoplakic and Erythroplakic Areas:**
   - **Presence of mixed white and red patches** suggests **epithelial dysplasia or carcinoma in situ**.
   - **Erythroleukoplakia carries a significantly higher malignant transformation potential** than leukoplakia alone.
4. **Verrucous-Like Features:**
   - The lesion displays **verrucous and exophytic growth**, which could indicate:
     - **Verrucous carcinoma** (if slow-growing, non-ulcerative, and well-differentiated).
     - **Squamous cell carcinoma** (if ulcerated, infiltrative, and rapidly progressing).

Most Probable Diagnosis:

Oral Squamous Cell Carcinoma (OSCC)

- **Key clinical features supporting SCC diagnosis include:**
  - **Non-healing ulceration with induration**
  - **Exophytic and infiltrative growth pattern**
  - **Presence of erythroleukoplakia**
  - **Irregular, roughened, and verrucous surface**

Given these features, **OSCC is the leading differential diagnosis and must be ruled out with a biopsy**.

Differential Diagnoses to Consider:

1. **Verrucous Carcinoma**
    - Typically presents as a **slow-growing, exophytic lesion** with **less ulceration** than SCC.
    - **Histopathology is necessary to differentiate between SCC and verrucous carcinoma.**
2. **Chronic Traumatic Ulcer or Hyperplasia**
    - Consider if there is **a history of persistent mechanical irritation (e.g., sharp teeth, dental prosthesis)**.
    - **Unlike SCC, reactive lesions are typically non-indurated and resolve once the source of trauma is removed.**
3. **Chronic Infectious Ulcers (Tuberculosis, Syphilis, Deep Fungal Infections)**
    - These conditions can cause **persistent ulcerations**, but typically lack the exophytic, verrucous component seen here.

Recommended Next Steps:

1. Urgent Incisional Biopsy for Histopathological Diagnosis

- A **deep incisional biopsy with adequate sampling of the lesion's central and peripheral areas** is mandatory.
- **Preferred biopsy site:** The most representative area, avoiding necrotic or superficial regions.
- The **histopathological report should assess:**
    - Presence of **keratin pearls, dysplastic changes, and invasive epithelial nests** (indicative of SCC).
    - **Differentiation grade** (well, moderately, or poorly differentiated).
    - **Depth of invasion** (critical for staging and prognosis).
    - **Presence of lymphovascular or perineural invasion**.

2. Imaging for Tumor Staging

- If SCC is confirmed, **imaging is required to evaluate local invasion and regional metastasis.**
- **Recommended imaging modalities:**
    - **MRI of the tongue and floor of the mouth** – to assess tumor extent and muscular infiltration.
    - **CT scan (head, neck, and chest)** – to evaluate lymph node involvement and distant metastases.
    - **Ultrasound with fine-needle aspiration biopsy (FNAB)** – if cervical lymphadenopathy is detected.

3. Comprehensive Oncologic Evaluation

- Referral to a **head and neck cancer multidisciplinary team (MDT)**.
- **TNM Staging (AJCC 8th Edition) should be established** to guide treatment planning.
- **If SCC is confirmed, a treatment plan must be discussed based on:**
  - **Tumor size (T staging).**
  - **Lymph node involvement (N staging).**
  - **Presence of distant metastasis (M staging).**

4. Risk Factor Assessment and Patient Optimization

- **Evaluate for risk factors contributing to malignant transformation:**
  - **Tobacco use (smoking or smokeless tobacco).**
  - **Heavy alcohol consumption.**
  - **HPV-associated carcinogenesis (p16 immunohistochemistry if suspected).**
- **Management of predisposing factors should be part of the long-term strategy.**

5. Definitive Treatment (Based on Staging)

- If SCC is confirmed and **localized (T1-T2, N0), surgery is the preferred treatment**:
  - **Wide local excision with adequate margins (≥1 cm).**
  - **Neck dissection (if nodal involvement is suspected).**
- If SCC is **advanced (T3-T4, N+), multimodal therapy is indicated:**
  - **Surgery + adjuvant radiation ± chemotherapy**.
  - **Definitive chemoradiotherapy if unresectable.**

Conclusion:

- **This lesion exhibits high-risk clinical features for oral squamous cell carcinoma (OSCC).**
- **Immediate incisional biopsy is mandatory for histopathological confirmation.**
- **If SCC is confirmed, comprehensive staging with imaging and multidisciplinary oncologic assessment must follow.**
- **Treatment planning should be based on TNM staging, patient comorbidities, and functional outcomes.**

Given the aggressive nature of OSCC, early diagnosis and timely intervention are critical for optimizing prognosis.

CASE 63

Reference:

https://www.mdanderson.org/content/mda/en/patients-family/diagnosis-treatment/cancer-types/oral-cancer/jcr:content/mainparsys/columns_2/column1/imageslideshow/slides/3/image.resize.j1054829665.jpg

Chat-GPT4 answer:

Analysis and Probable Diagnosis

Observations:

1. **Erythematous, irregularly bordered lesion on the lateral tongue**:
   - The lesion appears **well-demarcated, erythematous, and atrophic**.
   - There are **areas of epithelial thinning with a whitish periphery**.
   - The lesion does **not show ulceration, induration, or exophytic growth**.
2. **Location on the Lateral Border of the Tongue**:
   - The **lateral tongue** is a **high-risk area** for both **trauma-related lesions and precancerous changes**.
3. **No Clear Features of Infection, Trauma, or Malignancy**:
   - The lesion is **not ulcerated, not nodular, and lacks necrosis**.
   - There are **no vesicles or pseudomembranes**, making an infectious etiology less likely.

Most Probable Diagnosis: Erythroleukoplakia (Possible Epithelial Dysplasia)

- **Erythroleukoplakia** is a **potentially malignant disorder (PMD)** that combines **erythroplakia (red atrophic areas) and leukoplakia (whitish hyperkeratotic areas)**.
- **Key concerns:**
  - **Erythroplakia has the highest malignant transformation rate** (~40%).
  - **Biopsy is mandatory to rule out epithelial dysplasia or carcinoma in situ**.

Differential Diagnoses to Consider:

1. **Geographic Tongue (If Migratory and Asymptomatic)**
   - Similar **erythematous atrophic patches with white borders**.
   - **Migratory nature** is the key distinguishing feature.
2. **Lichenoid Mucositis (If Associated with Reticular White Striae)**
   - If the lesion has a **fine reticular pattern (Wickham's striae)**, it could indicate **oral lichen planus or a lichenoid reaction**.
3. **Traumatic Erythema (If History of Chronic Irritation)**
   - Could result from **repetitive tongue irritation** (sharp restorations, bruxism).
   - **Would resolve once trauma is removed**.
4. **Early Squamous Cell Carcinoma (If Persistent and Indurated)**
   - If the lesion is **persistent, non-healing, or associated with nodularity**, biopsy is essential.

- **High-risk factors: Smoking, alcohol, HPV infection**.

Recommended Next Steps:

1. **Mandatory Biopsy**:
    - **Incisional biopsy** to assess for **epithelial dysplasia, carcinoma in situ, or SCC**.
2. **Evaluate Risk Factors**:
    - **History of tobacco, alcohol, or HPV exposure?**
    - **Chronic trauma from dental restorations or prostheses?**
3. **Regular Follow-Up**:
    - If biopsy confirms **dysplasia, close monitoring every 3–6 months is required**.
4. **Management Based on Biopsy Results**:
    - **Mild dysplasia** → Conservative management and risk factor modification.
    - **Severe dysplasia or carcinoma in situ** → Surgical excision.

Conclusion

This lesion is **most concerning for erythroleukoplakia with a high risk of epithelial dysplasia**. **An urgent biopsy is required to determine histologic changes and guide management**. Would you like **a biopsy protocol or a guideline on managing potentially malignant oral disorders (PMDs)?**

CASE 64

Reference:

https://ars.els-cdn.com/content/image/1-s2.0-S1607551X11000349-gr1.jpg

Chat-GPT4 answer:

Analysis and Probable Diagnosis

Observations:

1. **Solitary ulcerated lesion on the ventral tongue**:
   - The lesion appears **well-defined, round, and covered with a fibrinous yellowish-white pseudomembrane**.
   - The surrounding mucosa is **erythematous, indicating inflammation**.
2. **No Evidence of Exophytic Growth or Induration**:
   - The lesion does not appear **nodular or proliferative**.
   - No signs of **malignant transformation or extensive tissue destruction**.
3. **Localized and Superficial Presentation**:
   - The lesion is **limited to the ventral tongue, without systemic involvement**.

Most Probable Diagnosis: Minor Aphthous Ulcer

- **Minor aphthous ulcers (canker sores)** are the **most common oral ulcerations**, typically presenting as:
  - **Painful, round or oval lesions** with a **necrotic pseudomembranous base**.
  - **Erythematous margins**, indicating localized inflammation.
  - **Self-limiting**, healing within **7–14 days without scarring**.
- **Etiology**:
  - **Immune-mediated response** (possibly triggered by stress, trauma, or nutritional deficiencies).
  - Can be **idiopathic or associated with systemic conditions**.

Differential Diagnoses to Consider:

1. **Herpetic Ulcer (If Multiple Small Vesicles Precede the Ulcer)**
   - **Primary or recurrent HSV** presents as **clusters of vesicles that rupture into ulcers**.
   - **Painful and typically involve the gingiva or lips**.
2. **Traumatic Ulcer (If History of Mechanical Injury)**
   - Can be caused by **biting, sharp teeth, or dental appliances**.
   - Typically **heals quickly once the irritant is removed**.
3. **Behçet's Disease (If Multiple Recurrent Ulcers + Genital Ulcers)**
   - **Chronic, recurrent aphthous ulcers** may suggest **Behçet's disease**.
   - Look for **genital ulcers, ocular involvement, or skin lesions**.
4. **Oral Syphilis (If Painless and Indurated)**
   - **Primary syphilis chancre** is typically a **painless, firm ulcer**.
   - Requires **serologic testing (VDRL, FTA-ABS)** if suspected.

5. **Oral Tuberculosis (If Chronic and Non-Healing)**
   - **Persistent, non-healing ulcer** in an immunocompromised patient could indicate **tuberculosis**.
   - Requires **biopsy and AFB staining**.

Recommended Next Steps:

1. **Symptomatic Treatment for Aphthous Ulcers**:
   - **Topical corticosteroids** (clobetasol gel or triamcinolone) to reduce inflammation.
   - **Chlorhexidine mouthwash** to prevent secondary infection.
   - **Lidocaine gel** for pain management.
2. **Monitor for Resolution Within 10–14 Days**:
   - If the ulcer **heals spontaneously**, no further action is needed.
   - If the ulcer **persists >3 weeks, biopsy is mandatory** to rule out malignancy or systemic disease.
3. **Investigate Systemic Conditions if Recurrent**:
   - If the patient has **frequent aphthous ulcers**, consider:
     - **Nutritional deficiencies (iron, B12, folate)**.
     - **Gastrointestinal disorders (Crohn's, celiac disease)**.
     - **Autoimmune conditions (Behçet's, lupus)**.

Conclusion

This lesion is **most likely a minor aphthous ulcer**. **No biopsy is required unless it persists beyond 3 weeks or recurs frequently**. **First-line management includes topical corticosteroids, pain relief, and supportive care**. Would you like **a differential guide for chronic oral ulcers or a systemic evaluation checklist for recurrent aphthous stomatitis?**

CASE 65

Reference:
https://enfamilia.aeped.es/sites/enfamilia.aeped.es/files/images/articulos/mucocele.jpg

Chat-GPT4 answer:

Analysis and Probable Diagnosis

Observations:

1. **Well-circumscribed, fluid-filled swelling on the lower lip**:
   - The lesion appears **soft, dome-shaped, and bluish-red**.
   - There is **no ulceration or induration**.
   - The surrounding mucosa is **intact and mildly erythematous**.
2. **Localized, non-tender lesion**:
   - The lesion does not appear to be associated with **systemic symptoms or widespread oral involvement**.
3. **History Suggestive of Trauma or Lip Biting**:
   - Lower lip lesions of this nature are **commonly associated with chronic mechanical irritation**.

Most Probable Diagnosis: Mucocele (Mucous Extravasation Cyst)

- Mucoceles occur due to **rupture of minor salivary gland ducts**, leading to **mucin accumulation in soft tissues**.
- Common features:
  - **Fluctuant, translucent, bluish swelling**.
  - **Recurrent history of rupture and refilling**.
  - Most common in the **lower lip, followed by the floor of the mouth (ranula)**.
  - **Painless unless secondarily infected**.

Differential Diagnoses to Consider:

1. **Fibroma (If Firm and Non-Fluctuant)**
   - A **firm, well-circumscribed, sessile lesion**.
   - **Does not fluctuate or rupture**.
   - More likely if the lesion is **chronic and non-translucent**.
2. **Hemangioma (If Compressible and Blanching)**
   - A **vascular lesion that blanches under pressure**.
   - Typically present from **childhood**.
3. **Minor Salivary Gland Tumor (If Persistent and Non-Fluctuant)**
   - **Mucoepidermoid carcinoma** can mimic a mucocele.
   - Unlike mucoceles, **tumors do not regress or fluctuate**.
4. **Herpetic Vesicles (If Multiple and Painful)**
   - Primary **HSV-1 presents as multiple vesicles that ulcerate**.
   - **Painful, unlike mucoceles**.

Recommended Next Steps:

1. **Observation if Recurrent and Self-Resolving**:
    - If the lesion **spontaneously resolves and recurs**, it is likely a **mucocele**.
2. **Surgical Excision if Persistent**:
    - **Mucoceles require complete excision with removal of the affected minor salivary gland lobules** to prevent recurrence.
    - Simple **aspiration is not effective** due to high recurrence rates.
3. **Histopathologic Examination if Atypical Features Are Present**:
    - If the lesion is **firm, fixed, or growing over time**, a **biopsy is mandatory** to rule out neoplasia.

Conclusion

This lesion is **most consistent with a mucocele (mucous extravasation cyst)**, a **benign and self-limiting salivary gland disorder**. If **persistent or recurrent, excision with histopathology is recommended**. Would you like **a procedural guide for mucocele excision or a differential diagnosis table for salivary gland lesions?**

CASE 66

Reference:

https://www.pcds.org.uk/imager/gallery/clinical/mucocoele/12501/Muco_1_fee391183f15cb4d62773032fe0be92d.jpg

Chat-GPT4 answer:

Analysis and Probable Diagnosis

Observations:

1. **Well-defined, dome-shaped, fluid-filled lesion on the lower lip**:
    - The lesion is **smooth, translucent, and bluish in appearance**.
    - It appears **soft, fluctuant, and compressible**.
2. **No Signs of Induration, Ulceration, or Erythema**:
    - There is **no surrounding inflammation or signs of infection**.
    - The lesion appears **localized without spreading or induration**.
3. **Common Location on the Lower Lip**:
    - **Most mucocele cases occur on the lower lip**, often due to **minor salivary gland duct obstruction or trauma**.

Most Probable Diagnosis: Mucocele (Mucous Retention Cyst)

- **Mucoceles** are **benign, self-limiting lesions** caused by the **extravasation or retention of saliva** due to:
    - **Trauma (lip biting, sucking habits, or accidental injury)**.
    - **Obstruction of minor salivary gland ducts**.
- They appear as **painless, fluctuant, dome-shaped swellings**.
- **Commonly found on the lower lip, buccal mucosa, and ventral tongue**.

Differential Diagnoses to Consider:

1. **Mucoepidermoid Carcinoma (If Persistent or Growing)**
    - A **rare but important differential** for a persistent, firm, and progressively enlarging lesion.
    - Requires **biopsy if the lesion does not resolve or recurs after excision**.
2. **Fibroma (If the Lesion is Firm and Not Fluid-Filled)**
    - Fibromas are **firm, non-fluctuant, well-defined nodules**.
    - Typically arise due to **chronic irritation or trauma**.
3. **Hemangioma (If the Lesion Blanches on Pressure)**
    - Hemangiomas are **vascular lesions that blanch with pressure**.
    - Unlike mucoceles, they do not contain mucin.
4. **Salivary Duct Cyst (If True Epithelial-Lined Cyst is Present)**
    - Similar to mucoceles but involves a **true epithelial-lined cyst**.
    - Diagnosed via **histopathology after excision**.

Recommended Next Steps:

1. **Observation and Conservative Management**:
   - Many mucoceles **spontaneously rupture and resolve**.
   - If the lesion is **small, asymptomatic, and not recurrent, no treatment is necessary**.
2. **Surgical Excision if Persistent or Recurrent**:
   - **Complete excision with removal of affected minor salivary glands is recommended** to prevent recurrence.
   - **Cryotherapy, laser ablation, or marsupialization** are alternative treatments.
3. **Biopsy if the Lesion Persists Beyond 3 Months**:
   - If the lesion **fails to resolve, recurs, or exhibits unusual features**, histopathologic evaluation is necessary.

Conclusion

This lesion is **most likely a mucocele**, a benign salivary gland lesion caused by trauma or duct obstruction. **Observation is advised, but excision is warranted if it persists, enlarges, or recurs.** Would you like a **surgical excision guide or management protocol for recurrent mucoceles?**

CASE 67

Reference:

https://www.odontoespacio.net/img/noticia/noticia_368.jpg

Chat-GPT4 answer:

Analysis and Probable Diagnosis

Observations:

1. **Midline, well-defined, nodular swelling on the hard palate**:
   - Appears **bilobed or rounded**, symmetrical, and firm.
   - Mucosa is **intact, normal in color, without ulceration or inflammation**.
   - No signs of **necrosis, exophytic growth, or rapid expansion**.
2. **Absence of Induration, Erythema, or Ulceration**:
   - No features suggesting **malignancy or inflammatory processes**.
   - The lesion appears **slow-growing, consistent with a benign condition**.
3. **No Evidence of Acute Infection or Reactive Hyperplasia**:
   - No **pus, discharge, or fluctuance**, ruling out abscesses or cystic infections.

Most Probable Diagnosis: Torus Palatinus

- **Torus palatinus** is a **benign, bony exostosis** that occurs on the midline of the hard palate.
- **Key features**:
  - **Hard, non-tender, midline palatal swelling**.
  - **Slow-growing and asymptomatic** unless traumatized.
  - More common in **females and certain ethnic groups (Asian and Native American populations)**.

Differential Diagnoses to Consider:

1. **Pleomorphic Adenoma (If Growth is Progressive)**
   - **Most common benign salivary gland tumor**.
   - If the lesion is **painless but growing**, biopsy should be considered.
2. **Fibrous Hyperplasia (If Associated with Dentures or Trauma)**
   - If the patient wears **ill-fitting dentures**, it could be a **reactive hyperplasia**.
3. **Osteoma (If Slow Growing and Hard on Palpation)**
   - A **benign bony tumor**, but less common in the palate.
4. **Nasopalatine Duct Cyst (If Soft and Midline)**
   - If the lesion has **fluctuance or is compressible**, imaging (CBCT) can confirm a cystic origin.
5. **Malignant Neoplasm (If Rapidly Expanding or Ulcerated)**
   - **Mucoepidermoid carcinoma** or **adenoid cystic carcinoma** may rarely arise in the palate.
   - **Biopsy is needed if the lesion is asymmetrical, ulcerated, or rapidly enlarging**.

Recommended Next Steps:

1. **Clinical Observation (If Consistent with Torus Palatinus)**
    - **No treatment is necessary unless it interferes with speech, eating, or denture fitting**.
2. **Imaging (CBCT or Panoramic X-ray If Needed)**
    - If the diagnosis is uncertain, imaging can confirm **bony density and exclude cystic lesions**.
3. **Biopsy Indications**:
    - If the lesion is **rapidly growing, ulcerated, or asymmetrical**.
    - If **pain or functional impairment** develops.
4. **Surgical Excision (Only If Necessary)**
    - Excision is considered if the **torus interferes with prosthetic appliances or is frequently traumatized**.

Conclusion

This lesion is **most likely a torus palatinus**, a **benign bony exostosis** requiring no intervention unless symptomatic. If there are **rapid changes in size, pain, or ulceration, further evaluation with imaging or biopsy is warranted**. Would you like **a guideline on surgical removal or a differential diagnosis for palatal masses?**

CASE 68

Reference:

https://www.codental.com.br/blog/wp-content/uploads/2023/06/torus-palatino.jpg

Chat-GPT4 answer:

Analysis and Probable Diagnosis

Observations:

1. **Prominent, well-defined midline swelling on the posterior hard palate**:
   - The lesion appears **smooth, dome-shaped, and symmetrical**.
   - It is **covered with intact mucosa, without ulceration or induration**.
   - No apparent **erythema, necrosis, or signs of infection**.
2. **No Evidence of Malignancy or Inflammation**:
   - The lesion is **well-circumscribed** and does not exhibit **irregular borders or rapid growth**.
   - There are **no exophytic growths or ulcerative changes** that would suggest a malignant process.
3. **Location on the Midline of the Hard Palate**:
   - A **bony or soft tissue origin** is suspected, given the midline placement.

Most Probable Diagnosis: Torus Palatinus

- **Torus palatinus** is a **benign, slow-growing bony exostosis** of the midline hard palate.
- Common characteristics:
  - **Hard, non-tender swelling**.
  - **Midline location on the hard palate**.
  - **Symmetric, well-defined borders**.
  - Typically **asymptomatic unless ulcerated from trauma**.
- **Etiology**:
  - Considered a **developmental anomaly**.
  - More common in **females** and **certain ethnic groups** (e.g., Asian and Native American populations).
  - May be **associated with parafunctional habits (bruxism, clenching).**

Differential Diagnoses to Consider:

1. **Palatal Cyst (If Soft and Fluctuant)**
   - If the lesion were **soft, compressible, or had a bluish hue**, a **palatal cyst (nasopalatine duct cyst, mucous retention cyst)** would be considered.
   - **Radiographic imaging (CBCT or occlusal X-ray) would confirm a cystic nature.**
2. **Minor Salivary Gland Tumor (If Progressive Growth)**
   - **Pleomorphic adenoma** is the most common benign salivary gland tumor in the palate.

- Typically presents as a **slow-growing, firm mass** that can eventually **ulcerate or cause discomfort**.
- **Biopsy is needed if the lesion is enlarging.**

3. **Fibrous Hyperplasia (If Soft and Mobile)**
   - If the lesion were **soft and mobile**, reactive fibrous hyperplasia from **chronic irritation** could be suspected.
4. **Osteoma (If Associated with Gardner's Syndrome)**
   - If the patient has **multiple bony growths, colonic polyps, or epidermoid cysts**, consider **Gardner's syndrome**.

Recommended Next Steps:

1. **Clinical Palpation**:
   - If the lesion is **hard and non-tender**, it strongly supports a diagnosis of **torus palatinus**.
2. **Radiographic Confirmation (If Needed)**:
   - **Occlusal X-ray or CBCT** to confirm a bony exostosis if there is any diagnostic uncertainty.
3. **No Treatment Required (Unless Symptomatic)**:
   - **Observation is sufficient** unless the torus interferes with:
     - **Speech or prosthetic appliance fitting (e.g., dentures).**
     - **Frequent trauma and ulceration.**
   - Surgical removal is rarely needed.

Conclusion

This lesion is **most consistent with torus palatinus**, a **benign bony growth with no malignant potential**. **No treatment is necessary unless it causes functional issues**. Would you like a **surgical removal guideline for large tori or a differential diagnosis guide for midline palatal swellings?**

CASE 69

Reference:

https://www.pathologyoutlines.com/imgau/oralcavitylichenplanusMintline01.jpg

Chat-GPT4 answer:

Analysis and Probable Diagnosis

Observations:

1. **White, reticulated striations on the buccal mucosa**:
    - The lesion exhibits a **bilateral, lace-like, white pattern (Wickham's striae)**.
    - The surrounding mucosa appears **erythematous but non-ulcerated**.
2. **No Signs of Induration, Ulceration, or Exophytic Growth**:
    - The lesion is **flat, reticular, and follows a characteristic network-like pattern**.
    - No evidence of **necrosis, nodularity, or hyperkeratotic plaques**.
3. **Asymptomatic or Mild Discomfort**:
    - Patients with this condition often **report a burning sensation**, but many are asymptomatic.

Most Probable Diagnosis: Oral Lichen Planus (OLP) – Reticular Variant

- **Oral lichen planus (OLP)** is a **chronic inflammatory mucocutaneous disorder** with an autoimmune basis.
- **Reticular OLP is the most common form**, characterized by:
    - **White, lace-like striations (Wickham's striae)**.
    - **Symmetrical and bilateral distribution**.
    - **Commonly affects the buccal mucosa, tongue, and gingiva**.
    - **Asymptomatic or mild burning sensation**.

Differential Diagnoses to Consider:

1. **Lichenoid Drug Reaction (If History of Medication Use)**
    - Can mimic **OLP**, but often **asymmetric and associated with recent drug exposure** (NSAIDs, antihypertensives, antimalarials).
    - Resolves after **drug withdrawal**.
2. **Lichenoid Contact Reaction (If Localized to Dental Restorations)**
    - White striations or plaques **adjacent to amalgam or metal restorations**.
    - Resolves after **removal of the irritant**.
3. **Leukoplakia (If Homogeneous and Non-Reticular)**
    - **Leukoplakia is typically isolated and does not exhibit Wickham's striae**.
    - May be **precancerous**, requiring **biopsy for dysplasia assessment**.
4. **Lupus Erythematosus (If Systemic Symptoms Present)**
    - Oral lesions of **systemic lupus erythematosus (SLE)** may resemble OLP.
    - **Check for systemic symptoms (malar rash, arthritis, photosensitivity, ANA positivity).**

Recommended Next Steps:

1. **Clinical Monitoring and Symptomatic Management**:
   - If **asymptomatic**, no immediate treatment is required.
   - **Topical corticosteroids (clobetasol or fluocinonide) for symptomatic relief** if burning or discomfort is present.
2. **Biopsy if Atypical Features Are Present**:
   - If the lesion exhibits **ulceration, induration, or asymmetry**, biopsy is required to rule out **dysplasia or malignancy**.
3. **Regular Follow-Up (Every 6–12 Months)**:
   - OLP has a **low but present risk of malignant transformation (~1–2%)**.
   - If erosive changes develop, **biopsy is mandatory**.

Conclusion

This lesion is **most consistent with reticular oral lichen planus (OLP)**. If **asymptomatic, observation is sufficient**. If there is **discomfort, topical corticosteroids can help**. **Biopsy is indicated if ulceration, induration, or asymmetry is present**. Would you like **a treatment protocol for symptomatic OLP or a biopsy guideline for suspected malignant transformation?**

CASE 70

Reference:

https://www.patologia-orale.net/wp-content/uploads/2023/06/Lesioni-potenzialmente-maligne-scaled-e1687247306732.jpg

Chat-GPT4 answer:

Analysis and Probable Diagnosis

Observations:

1. **Thick, white, and corrugated plaques on the lateral borders of the tongue**:
   - The lesion is **bilateral**, non-uniform, and appears **hyperkeratotic**.
   - The white areas do **not appear to be easily scrapable**.
2. **Central Erythematous Zone on the Dorsal Tongue**:
   - The central part of the tongue shows **erythema with atrophic or depapillated areas**.
   - The surface appears **dry and fissured**, indicating possible chronic irritation.
3. **Absence of Ulceration, Nodularity, or Induration**:
   - The lesion appears **thickened and plaque-like**, without ulceration or firm induration.
   - No **exophytic mass or rolled borders**, making malignancy less likely at this stage.

Most Probable Diagnosis: Oral Hairy Leukoplakia (OHL)

- **OHL is a pathognomonic condition caused by Epstein-Barr Virus (EBV)**, often seen in **immunocompromised individuals (e.g., HIV, transplant recipients, chemotherapy patients)**.
- **Key Features:**
   - **Bilateral, corrugated white plaques on the lateral tongue**.
   - **Does not rub off**, unlike pseudomembranous candidiasis.
   - **Often asymptomatic, but some patients report mild irritation**.
   - Strongly associated with **HIV/AIDS and chronic immunosuppression**.

Differential Diagnoses to Consider:

1. **Leukoplakia (If No Immunosuppression History)**
   - **Homogeneous or non-homogeneous white plaques**, potentially dysplastic.
   - **High-risk sites: lateral tongue, floor of the mouth**.
   - **Requires biopsy if persistent beyond 2-3 weeks**.
2. **Pseudomembranous Candidiasis (If Scrapable White Lesions)**
   - Candidiasis presents as **white patches that can be scraped off**, leaving an **erythematous base**.
   - Common in **diabetics, steroid users, or immunocompromised patients**.
   - **Diagnosis: KOH test, PAS stain, or response to antifungal therapy**.
3. **Lichen Planus (If Reticular White Striae Present)**
   - Oral lichen planus presents as **fine white striae (Wickham's striae)** on the buccal mucosa or tongue.

- Biopsy confirms **lichenoid inflammation**.

4. **Oral Squamous Cell Carcinoma (If Ulceration or Induration Develops)**
   - If the lesion **becomes indurated, ulcerated, or progressively enlarges**, biopsy is mandatory.
   - Risk factors: **smoking, alcohol, HPV infection**.

Recommended Next Steps:

1. **HIV and EBV Serology**:
   - **If the patient has risk factors for immunosuppression, an HIV test is warranted**.
   - Epstein-Barr Virus (EBV) PCR or serology can confirm **OHL**.
2. **Biopsy if Atypical Features Appear**:
   - **Persistent, thickened, or nodular lesions require biopsy** to rule out **epithelial dysplasia or carcinoma**.
3. **Trial of Antifungal Therapy (If Superimposed Candidiasis is Suspected)**:
   - **Topical clotrimazole or systemic fluconazole** can help if fungal overgrowth is present.
4. **Regular Follow-Up and Risk Factor Modification**:
   - **Avoid smoking and alcohol**, which can increase the risk of malignant transformation.
   - Close monitoring for **any changes in size, texture, or ulceration**.

Conclusion

This lesion is **highly suggestive of Oral Hairy Leukoplakia (OHL), a classic EBV-related condition in immunosuppressed individuals**. **HIV testing and a thorough immunologic evaluation are recommended**. If atypical features appear, **biopsy should be performed to rule out dysplasia or carcinoma**. Would you like **a guideline on OHL management or biopsy protocols for persistent leukoplakia?**

CASE 71

Reference:

https://media.sciencephoto.com/c0/44/30/64/c0443064-800px-wm.jpg

Chat-GPT4 answer:

Analysis and Probable Diagnosis

Observations:

1. **Generalized hyperpigmentation of the tongue**:
   - The tongue appears **diffusely darkened, particularly in the central region**.
   - The pigmentation is **bilateral, symmetric, and involves most of the dorsal surface**.
2. **Absence of Ulceration, Induration, or Exophytic Growth**:
   - No visible **ulcers, nodules, or signs of inflammation**.
   - The tongue **surface appears slightly dry and coated**.
3. **Potential Lingual Papillary Changes**:
   - There may be some **hypertrophy or atrophy of the filiform papillae**.

Most Probable Diagnosis: Black Hairy Tongue (Lingua Villosa Nigra)

- **Black hairy tongue (BHT)** is a **benign condition** caused by **excessive keratinization and elongation of filiform papillae**.
- **Common triggers:**
  - **Poor oral hygiene**.
  - **Smoking or tobacco use**.
  - **Excessive coffee, tea, or alcohol consumption**.
  - **Antibiotic use (especially tetracyclines or metronidazole)**.
  - **Xerostomia (dry mouth) due to dehydration or medication**.

Differential Diagnoses to Consider:

1. **Physiologic Pigmentation (If the Patient Has Darker Skin)**
   - **Bilateral, symmetric pigmentation** is common in individuals with **darker skin tones**.
   - Unlike BHT, there is **no papillary elongation**.
2. **Smoker's Melanosis (If Associated with Tobacco Use)**
   - Tobacco exposure leads to **reactive melanosis** of the oral mucosa.
   - More commonly affects the **gingiva and buccal mucosa**.
3. **Fungal Overgrowth (If Associated with Burning Sensation)**
   - **Oral candidiasis** can present with a **dark coating that scrapes off**.
   - Fungal culture or KOH examination can confirm **Candida overgrowth**.
4. **Heavy Metal Toxicity (If Related to Occupational Exposure)**
   - **Lead, bismuth, or mercury poisoning** can cause **oral pigmentation**.
   - **Neurological symptoms** and **history of exposure** are key clues.
5. **Hyperpigmented Conditions (If Systemic Symptoms Exist)**

- **Addison's disease**: Associated with **diffuse mucosal pigmentation, fatigue, hypotension**.
- **Peutz-Jeghers syndrome**: Mucosal and lip pigmentation with **gastrointestinal polyps**.

Recommended Next Steps:

1. **Patient History and Risk Factor Assessment**:
   - **Smoking, alcohol, antibiotic use, or poor oral hygiene?**
   - **Recent illness, dehydration, or medication changes?**
   - **Any systemic symptoms like fatigue, weight loss, or GI issues?**
2. **Improve Oral Hygiene and Dietary Modifications**:
   - **Brushing the tongue with a soft toothbrush** or tongue scraper.
   - **Discontinuing smoking, excessive coffee, or alcohol**.
   - **Staying hydrated** to reduce dry mouth.
3. **Consider Antifungal Therapy if Candida is Suspected**:
   - If there is a **burning sensation or white overlay**, antifungal treatment may be indicated.
4. **Monitor for Spontaneous Resolution**:
   - **Black hairy tongue is self-limiting** and improves with good oral hygiene.
   - If persistent beyond **several months**, evaluate for **systemic conditions or heavy metal exposure**.

Conclusion

This lesion is **most consistent with black hairy tongue (BHT), a benign condition associated with poor oral hygiene, smoking, or antibiotic use**. **Management involves improving oral hygiene, reducing risk factors, and monitoring for resolution**. Would you like **a guide on oral hygiene management for BHT or additional differential considerations for oral pigmentation?**

CASE 72

Reference:

https://i0.wp.com/images-prod.healthline.com/hlcmsresource/images/Lifestyle/Thrush-on-the-tongue-1296x728-slide1.jpg?w=1155&h=1528

Chat-GPT4 answer:

Analysis and Probable Diagnosis

Observations:

1. **Thick, white coating covering the dorsum of the tongue**:
   - The lesion appears **diffuse, non-uniform, and thickly adherent**.
   - The coating is **more prominent in the central and posterior tongue**.
   - The tongue papillae appear **erythematous and inflamed at the periphery**.
2. **Erythematous Papillae at the Tip and Lateral Borders**:
   - The presence of **red, inflamed fungiform papillae** suggests **underlying irritation or inflammation**.
3. **No Evidence of Ulceration, Induration, or Exophytic Growth**:
   - This reduces the likelihood of **malignancy or premalignant conditions**.

Most Probable Diagnosis: Oral Candidiasis (Pseudomembranous Candidiasis)

- **Pseudomembranous candidiasis ("thrush")** is a fungal infection caused by **Candida albicans**.
- **Key clinical features:**
  - **White, removable plaques on the oral mucosa**.
  - **Red, inflamed mucosa underneath the plaque if scraped**.
  - **Common in immunocompromised patients, those using inhaled corticosteroids, or with recent antibiotic use**.

Differential Diagnoses to Consider:

1. **Hairy Leukoplakia (If Present on the Lateral Borders of the Tongue)**
   - Caused by **Epstein-Barr virus (EBV) in immunocompromised patients** (e.g., HIV).
   - **Non-removable, corrugated white plaques** along the lateral tongue.
   - Does not wipe off like candidiasis.
2. **Geographic Tongue (If Migratory and Irregular)**
   - **Patchy, depapillated areas with white borders**.
   - Lesions migrate over time.
   - No fungal involvement.
3. **Coated Tongue (If Poor Oral Hygiene and Debris Accumulation)**
   - Thick coating due to **bacteria, food debris, or dehydration**.
   - Can be improved with oral hygiene measures.
4. **Lichen Planus (If White Reticular Striae Present)**
   - **Wickham's striae (lacy white pattern)** may suggest **lichen planus**.
   - Typically presents as **chronic and bilateral**.

Recommended Next Steps:

1. **Confirm Diagnosis with Scraping Test**:
   - **Gently scrape the lesion** with a tongue blade:
     - If it wipes off, leaving an **erythematous surface**, it is likely **candidiasis**.
     - If it does not, consider **hairy leukoplakia or lichen planus**.
2. **Identify Predisposing Factors**:
   - **Recent antibiotic use or corticosteroid inhaler?**
   - **Diabetes, immunosuppression (e.g., HIV, chemotherapy)?**
   - **Poor oral hygiene or dry mouth?**
3. **Initiate Antifungal Therapy**:
   - **Topical antifungals**: Nystatin suspension, clotrimazole troches.
   - **Systemic antifungals**: Fluconazole (if extensive or recurrent).
4. **Improve Oral Hygiene and Manage Risk Factors**:
   - **Good tongue brushing** to reduce plaque accumulation.
   - **Probiotics if post-antibiotic candidiasis is suspected**.
   - **HIV or diabetes screening if immunosuppression is suspected**.

Conclusion

This lesion is **highly suggestive of pseudomembranous candidiasis**, likely due to fungal overgrowth. **Antifungal therapy and risk factor modification are essential**. If the lesion does **not respond to treatment, further evaluation (HIV, diabetes, or biopsy) is recommended**. Would you like **a treatment protocol for candidiasis or a differential diagnosis guide for white oral lesions?**

CASE 73

Reference:

https://static.elsevier.es/multimedia/03650596/0000009800000004/v1_202307031010/S0365059623000648/v1_202307031010/en/main.assets/gr1.jpeg

Chat-GPT4 answer:

Analysis and Probable Diagnosis

Observations:

1. **White, linear, and reticular lesion on the buccal mucosa**:
   - The lesion appears **bilateral, white, and lace-like**.
   - The pattern is **not uniform but has a reticulated or striated appearance**.
   - No evidence of **ulceration, nodularity, or significant erythema**.
2. **Well-Demarcated and Non-Wipeable Lesion**:
   - The lesion does not **appear to be pseudomembranous**, ruling out **candidiasis**.
   - The white areas **cannot be wiped off**, suggesting **keratotic changes**.
3. **Symmetrical Distribution**:
   - Typically, **bilateral and symmetrical** lesions suggest a **chronic immune-mediated condition** rather than an infectious or neoplastic process.

Most Probable Diagnosis: Reticular Oral Lichen Planus (OLP)

- **Oral lichen planus (OLP)** is a **chronic inflammatory mucocutaneous disorder** mediated by **T-cell activation**.
- The **reticular form** is characterized by:
  - **White, lace-like striae (Wickham's striae) on the buccal mucosa**.
  - **Bilateral presentation**, often involving the **tongue and gingiva**.
  - **Asymptomatic or mild burning sensation**.
- **Etiology**:
  - **Autoimmune reaction against basal keratinocytes**.
  - **Associated with systemic conditions (diabetes, hepatitis C, stress)**.

Differential Diagnoses to Consider:

1. **Frictional Keratosis (If Associated with Chronic Trauma)**
   - If due to **mechanical irritation (cheek biting, dental prostheses)**, removing the irritant leads to **lesion resolution**.
   - Typically **not lace-like and more focal**.
2. **Leukoplakia (If Solitary and Asymmetrical)**
   - Leukoplakia is **precancerous**, whereas OLP is **immune-mediated**.
   - **Leukoplakia does not have Wickham's striae** and requires **biopsy if persistent**.
3. **Oral Candidiasis (If Wipeable)**
   - **Pseudomembranous candidiasis (oral thrush)** can mimic OLP but **can be wiped off**, revealing an **erythematous base**.

4. **Lichenoid Drug Reaction (If Associated with Medication Use)**
    - Certain drugs (**NSAIDs, beta-blockers, ACE inhibitors**) can induce **lichenoid mucosal changes**.
    - Discontinuation of the offending drug leads to **resolution**.

Recommended Next Steps:

1. **Confirm Clinical Diagnosis**:
    - **Reticular OLP is typically diagnosed clinically**.
    - **Biopsy is recommended** if there is:
        - **Erosive or ulcerative lesions**.
        - **Persistent pain or burning**.
        - **Unilateral or irregular presentation** (to rule out dysplasia or carcinoma).
2. **Monitor for Malignant Transformation**:
    - **Oral lichen planus has a low but real risk (~1%) of malignant transformation** to **oral squamous cell carcinoma**.
    - **Regular follow-up every 6 months is advised**.
3. **Treatment (If Symptomatic)**:
    - **Asymptomatic OLP does not require treatment**.
    - If symptomatic (burning, discomfort), first-line therapy includes:
        - **Topical corticosteroids (clobetasol or fluocinonide gel)**.
        - **Chlorhexidine mouthwash** to reduce secondary infection.
        - **Systemic therapy** (e.g., systemic corticosteroids or immunosuppressants) in severe cases.
4. **Evaluate for Systemic Associations**:
    - **Screen for hepatitis C**, as it is associated with **OLP**.
    - Assess for **stress-related exacerbations**.

Conclusion

This lesion is **most consistent with reticular oral lichen planus**, a **chronic immune-mediated disorder**. **No biopsy is required unless there are erosive or atypical features**. **If symptomatic, topical corticosteroids are recommended**. Would you like **a follow-up protocol for monitoring OLP or a management guide for symptomatic cases?**

CASE 74

Reference:

https://www.divinedentalcare.com.au/wp-content/uploads/2017/01/1-3.jpg

Chat-GPT4 answer:

Analysis and Probable Diagnosis

Observations:

1. **Generalized gingival inflammation and erythema**:
   - The gingiva appears **swollen, erythematous, and hyperemic**.
   - There is **gingival edema, with marginal and papillary involvement**.
   - The inflammation is **diffuse**, affecting both upper and lower arches.
2. **Accumulation of Dental Plaque and Calculus**:
   - Visible **plaque deposits and calculus formation**, particularly along the **cervical third of the teeth**.
   - The **plaque-induced inflammation** suggests a **chronic periodontal disease** process.
3. **Gingival Bleeding and Possible Early Attachment Loss**:
   - The **gingival margin appears friable**, with potential **spontaneous or provoked bleeding**.
   - There may be **initial attachment loss**, though no clear signs of advanced periodontitis (e.g., recession, deep pockets) are visible.
4. **Enamel Erosion and Tooth Wear**:
   - Evidence of **dental erosion and attrition** on the incisal and occlusal surfaces.
   - Could be related to **bruxism, acidic diet, or poor oral hygiene**.

Most Probable Diagnosis: Chronic Gingivitis with Plaque-Induced Periodontal Disease

- **Chronic gingivitis** is the **most common reversible form of periodontal disease**, characterized by:
  - **Plaque and calculus accumulation** leading to **gingival inflammation**.
  - **Bleeding on probing (BOP), edema, and erythema**.
  - **No irreversible bone loss or deep periodontal pockets** (distinguishing it from periodontitis).
- **Etiology**:
  - **Poor oral hygiene leading to plaque biofilm accumulation**.
  - **Bacterial invasion (e.g., Porphyromonas gingivalis, Fusobacterium nucleatum)**.
  - **Smoking, diabetes, or systemic conditions may exacerbate inflammation**.

Differential Diagnoses to Consider:

1. **Early Periodontitis (If Attachment Loss is Present)**
   - If periodontal probing reveals **pocket depths >4 mm with attachment loss**, it may indicate **progression from gingivitis to periodontitis**.
2. **Gingival Hyperplasia (If Overgrowth is Pronounced)**

- If the gingiva appears **excessively thickened**, consider:
    - **Drug-induced gingival overgrowth (e.g., phenytoin, calcium channel blockers, cyclosporine).**
    - **Leukemic gingival infiltration (if systemic symptoms like fatigue, bruising exist).**

3. **Necrotizing Ulcerative Gingivitis (NUG, If Painful with Ulceration)**
    - **Painful, necrotic interdental papillae** with a **fetid odor**.
    - Often associated with **stress, smoking, or immunosuppression**.
4. **Vitamin Deficiency-Associated Gingivitis (If Systemic Signs Are Present)**
    - **Vitamin C deficiency (scurvy)** can lead to **spontaneous gingival bleeding, swelling, and delayed healing**.

Recommended Next Steps:

1. **Comprehensive Periodontal Evaluation**:
    - **Periodontal probing** to assess for **attachment loss and pocket depths**.
    - **Radiographic evaluation (bitewings or periapical X-rays)** to check for **bone loss**.
2. **Professional Scaling and Root Planing (SRP)**
    - **Ultrasonic scaling** to remove plaque and calculus.
    - **Subgingival debridement** to reduce bacterial load.
3. **Oral Hygiene Reinforcement**:
    - **Twice-daily brushing with fluoridated toothpaste**.
    - **Interdental cleaning (floss or interdental brushes)**.
    - **Antimicrobial mouthwash (e.g., chlorhexidine 0.12%) for short-term control**.
4. **Reevaluation After 4-6 Weeks**:
    - If inflammation persists **despite good oral hygiene**, assess for:
        - **Systemic contributing factors (e.g., diabetes, smoking)**.
        - **Need for additional periodontal therapy (e.g., deeper scaling, local antimicrobials)**.
5. **Address Tooth Wear and Erosion**:
    - Assess for **bruxism (night guard if needed)**.
    - **Dietary counseling (avoid acidic foods, carbonated drinks)**.

Conclusion

This case is **consistent with plaque-induced chronic gingivitis**, with **potential early periodontal involvement**. **Scaling, oral hygiene modification, and follow-up evaluation are essential**. If there is **attachment loss or persistent inflammation, further periodontal therapy should be considered**. Would you like **a step-by-step periodontal treatment protocol or a guideline for monitoring periodontal disease progression?**

CASE 75

Reference:

https://www.pathologyoutlines.com/imgau/oralcavityirritationfibroma_smithbrq03.jpg

Chat-GPT4 answer:

Analysis and Probable Diagnosis

Observations:

1. **Well-defined, sessile, soft-tissue nodule on the buccal mucosa**:
   - The lesion appears **smooth, dome-shaped, and mucosal-colored**.
   - No evidence of **ulceration, necrosis, or inflammation**.
   - The lesion is **not indurated**.
2. **Located on the Posterior Buccal Mucosa Adjacent to the Maxillary Teeth**:
   - Common area for **fibrotic reactive lesions** due to **chronic irritation**.
3. **No Features of Infection, Malignancy, or Systemic Disease**:
   - The lesion does **not exhibit rapid growth, pain, or ulceration**.

Most Probable Diagnosis: Irritation Fibroma (Fibrous Hyperplasia)

- This is a **benign, reactive lesion** caused by **chronic irritation or trauma** (e.g., cheek biting, sharp restorations).
- **Key features:**
  - **Firm, well-defined, slow-growing nodule**.
  - **Asymptomatic unless traumatized**.
  - Typically occurs on the **buccal mucosa, tongue, or labial mucosa**.

Differential Diagnoses to Consider:

1. **Minor Salivary Gland Tumor (If Persistent and Firm)**
   - **Pleomorphic adenoma** or **mucoepidermoid carcinoma** may present as **slow-growing, firm nodules**.
   - If the lesion grows or becomes **fixed**, a **biopsy is required**.
2. **Lipoma (If Soft and Yellowish)**
   - **Soft, compressible mass with a yellowish hue**.
   - More common in **older adults**.
3. **Neurofibroma or Schwannoma (If Associated with Nerve Symptoms)**
   - If **tingling or altered sensation** is present, a **neural tumor should be suspected**.
4. **Mucosal Neuroma (If Multiple and Part of a Syndrome)**
   - **Multiple mucosal neuromas** can indicate **Multiple Endocrine Neoplasia type 2B (MEN 2B)**.

Recommended Next Steps:

1. **Assess for Chronic Irritation**:

    - **History of cheek biting, sharp restorations, or dental prostheses?**
    - If an irritant is identified, **removal may prevent recurrence**.
2. **Observation vs. Biopsy**:
    - If **stable and small**, no immediate intervention is needed.
    - **Excisional biopsy** is recommended if:
        - The lesion is **growing or symptomatic**.
        - There is **concern for a neoplasm**.
3. **Complete Excision if Persistent**:
    - **Simple excision is curative**.
    - **Histopathology confirms fibrous hyperplasia vs. other soft tissue lesions**.

Conclusion

This lesion is **most consistent with an irritation fibroma**, a **benign hyperplastic response to chronic trauma**. If the lesion is **asymptomatic and stable, observation is acceptable**. If it **continues to enlarge, biopsy is advised to exclude neoplastic lesions**. Would you like **a procedural guide for excision or a differential diagnosis guide for oral soft-tissue swellings?**

CASE 76

Reference:

https://www.researchgate.net/publication/318249904/figure/fig5/AS:1086534186999818@1636061345763/Irritation-fibroma-on-the-buccal-mucosa-with-a-smooth-surface-and-dome-shaped-base.jpg

Chat-GPT4 answer:

Analysis and Probable Diagnosis

Observations:

1. **Well-circumscribed, smooth, dome-shaped nodule on the buccal mucosa**:
   - The lesion appears **pinkish, firm, and round**.
   - It is **non-ulcerated, non-fluctuant**, and has a **slightly raised surface**.
2. **Presence of a Foreign Object or Calcified Material Adjacent to the Lesion**:
   - There appears to be **a dark-greenish, possibly necrotic or foreign object near the lesion**.
   - This suggests a possible **reactive or inflammatory etiology**.
3. **No Signs of Ulceration, Induration, or Exophytic Growth**:
   - The lesion does not appear **malignant** at first glance.
   - The **absence of necrosis, irregular borders, or tissue destruction** reduces suspicion for **malignancy**.

Most Probable Diagnosis: Fibroma (Traumatic or Irritational Fibroma)

- **Fibromas** are **benign, reactive connective tissue overgrowths** due to chronic irritation or trauma.
- **Key characteristics:**
  - **Slow-growing, firm, and non-painful nodule**.
  - Common locations: **buccal mucosa, tongue, or lips**.
  - **Does not ulcerate unless secondarily traumatized**.
  - **Does not resolve spontaneously**.
- **Possible cause in this case**:
  - Chronic irritation due to **dental restorations, biting habits, or an embedded foreign object**.

Differential Diagnoses to Consider:

1. **Mucocele (If Fluid-Filled and Fluctuant)**
   - If the lesion is **soft, bluish, and compressible**, it may be a **mucocele**.
   - Mucoceles result from **salivary gland duct rupture**, leading to **mucin accumulation**.
2. **Salivary Gland Tumor (If Slowly Growing and Firm)**
   - A **pleomorphic adenoma** or **low-grade mucoepidermoid carcinoma** could present similarly.
   - Requires **biopsy if the lesion is persistent or growing**.

3. **Granuloma (If Related to Foreign Body or Infection)**
   - A **foreign body granuloma** may develop around **embedded material** (e.g., amalgam tattoo or foreign material).
4. **Peripheral Ossifying Fibroma (If Firm and Calcified)**
   - If **calcification is present**, consider **peripheral ossifying fibroma**, a reactive gingival lesion.
5. **Minor Salivary Gland Tumor (If Persistent and Not Traumatic)**
   - If the lesion **persists despite trauma removal**, a **minor salivary gland neoplasm** should be ruled out.

Recommended Next Steps:

1. **Assess for Local Trauma or Irritants**:
   - **Check for sharp restorations, parafunctional habits (cheek biting), or embedded materials**.
2. **Conservative Excision for Histopathologic Diagnosis**:
   - **Complete excision** is recommended, especially if the lesion **persists, grows, or is symptomatic**.
   - Histopathology will confirm if it is a **fibroma, granuloma, or another lesion**.
3. **Removal of Any Foreign Object or Source of Irritation**:
   - The **greenish mass should be evaluated** to determine if it is **necrotic tissue, dental material, or foreign matter**.
4. **Follow-Up to Monitor for Recurrence**:
   - If excised, monitor for **recurrence**, which is rare unless the irritant persists.

Conclusion

This lesion is **most likely a traumatic or irritational fibroma**, possibly secondary to **chronic irritation from a foreign object or dental material**. **Surgical excision is the preferred treatment, with histopathologic confirmation.** Would you like **a histopathology report guide or a fibroma excision protocol?**

CASE 77

Reference:

https://cosmoderma.org/content/130/2024/4/1/img/CSDM-4-78-g001.png

Chat-GPT4 answer:

Analysis and Probable Diagnosis

Observations:

1. **Solitary, well-defined, dome-shaped lesion on the dorsal tongue**:
   - The lesion appears **smooth, round, and sessile (broad-based)**.
   - Pink, **homogeneous in color**, without ulceration or necrosis.
   - The surrounding mucosa appears **healthy, with no signs of inflammation**.
2. **Soft Tissue Mass Without Induration or Ulceration**:
   - The lesion **does not appear firm, nodular, or infiltrative**, making malignancy **less likely**.
   - No signs of **infection, vascularization, or secondary changes**.
3. **Asymptomatic and Slow-Growing Nature Likely**:
   - The lesion is **likely benign**, considering its **well-defined, non-ulcerated appearance**.

Most Probable Diagnosis: Irritation Fibroma or Mucosal Papule

- **Fibromas** are the most common **benign reactive soft tissue lesions of the oral cavity**.
- **Key Features:**
  - **Smooth, firm, and dome-shaped nodule**.
  - **Commonly caused by trauma or irritation (e.g., tongue biting, friction)**.
  - **Typically asymptomatic unless secondarily ulcerated**.

Differential Diagnoses to Consider:

1. **Squamous Papilloma (If Cauliflower-Like)**
   - **Caused by HPV (Human Papillomavirus, types 6 & 11)**.
   - Appears as a **pedunculated (stalked), verrucous, or finger-like projection**.
2. **Mucocele (If Soft and Fluctuant)**
   - If the lesion is **fluid-filled and compressible**, it could be a **mucocele**.
   - Common in **lower lip but can also occur on the tongue**.
3. **Lipoma (If Yellowish and Soft)**
   - **Soft, compressible, and yellowish lesions**.
   - Rare on the tongue but possible.
4. **Neurofibroma or Schwannoma (If Slow-Growing and Firm)**
   - If the lesion is **firm, enlarging, or associated with nerve symptoms (tingling, pain)**, consider a **nerve-related tumor**.
   - Requires **biopsy for confirmation**.
5. **Granular Cell Tumor (If Firm and Indurated)**
   - Rare but should be considered if the lesion is **firm and non-tender**.

- **Common on the tongue**.

Recommended Next Steps:

1. **Clinical Monitoring (If Small and Asymptomatic)**:
   - **No immediate intervention if the lesion remains stable**.
   - **Re-evaluate in 4–6 weeks** for any changes in size, color, or ulceration.
2. **Biopsy Indications**:
   - If the lesion **enlarges, becomes painful, ulcerates, or changes in texture**.
   - **Excisional biopsy is preferred for small lesions**.
3. **Surgical Excision (If Symptomatic or Aesthetic Concern)**
   - **Simple excision under local anesthesia is curative**.
   - Low recurrence if **traumatic irritants (e.g., tongue biting) are eliminated**.

Conclusion

This lesion is **most likely an irritation fibroma, a benign soft tissue lesion**. **No urgent intervention is required unless the lesion changes in size or becomes symptomatic**. If persistent, **surgical excision with histopathological examination is recommended**. Would you like **a surgical excision protocol or a guide on differentiating tongue papules?**

CASE 78

Reference:

https://images.squarespace-cdn.com/content/v1/5d26b16e3c8b190001ab4906/1611282802075-9KC5FVF2GUFSRNPZ7FFY/oral+tongue+cancer.png

Chat-GPT4 answer:

Analysis and Probable Diagnosis

Observations:

1. **Large, exophytic, and ulcerated mass on the dorsal surface of the tongue**:
   - The lesion is **irregular, lobulated, and partially ulcerated**.
   - It exhibits **white, necrotic areas interspersed with erythematous regions**.
   - The surface appears **indurated, verrucous, and proliferative**.
2. **Significant Enlargement and Asymmetry**:
   - The lesion **disrupts the normal anatomy of the tongue**.
   - There is **visible tissue infiltration and potential deep invasion**.
3. **No Signs of Acute Inflammatory Processes or Benign Reactive Lesions**:
   - The lesion does not present as **a simple ulcer or traumatic lesion**.
   - It does not exhibit a well-demarcated inflammatory border.

Most Probable Diagnosis: Squamous Cell Carcinoma (SCC) of the Tongue

- **Squamous cell carcinoma (SCC) is the most common malignant tumor of the oral cavity**.
- **High-risk factors** include:
  - **Tobacco and alcohol consumption** (synergistic effect).
  - **HPV-related oncogenesis** (particularly HPV-16 and HPV-18).
  - **Chronic irritation and poor oral hygiene**.
  - **Older age, male gender, and genetic predisposition**.
- **Key features suggestive of SCC in this case**:
  - **Exophytic, ulcerated mass with a rough surface**.
  - **Irregular, indurated, and asymmetric lesion**.
  - **White keratotic and necrotic patches (leukoplakic component)**.
  - **Possible deep tissue invasion**.

Differential Diagnoses to Consider:

1. **Verrucous Carcinoma (If Slow-Growing and Less Invasive)**
   - A **low-grade variant of SCC**.
   - Characterized by **wart-like growth without significant ulceration**.
   - Requires **biopsy to distinguish from conventional SCC**.
2. **Chronic Hyperplastic Candidiasis (If Pseudomembranous and Rubs Off)**
   - Presents with **thick white patches that may be scraped off**.
   - Less likely due to **extensive exophytic nature of this lesion**.
   - Confirmed with **PAS stain for fungal elements**.

3. **Proliferative Verrucous Leukoplakia (If Multiple and Slowly Evolving)**
    - A high-risk **premalignant disorder**.
    - Characterized by **multifocal white plaques with a verrucous pattern**.
    - Has a **high transformation rate to SCC**.
4. **Syphilitic Gumma (If Associated with Systemic Symptoms)**
    - **Tertiary syphilis can present with destructive ulcerative lesions**.
    - Confirmed by **serologic testing (VDRL, FTA-ABS).**
5. **Deep Fungal Infection (If Immunosuppression is Present)**
    - Blastomycosis or histoplasmosis can **mimic oral malignancies**.
    - Requires **biopsy and fungal culture** for confirmation.

Recommended Next Steps:

1. **Urgent Incisional Biopsy**:
    - Histopathologic examination with **immunohistochemistry (p16 for HPV, CK5/6 for SCC)**.
    - Differentiation between **keratinizing and non-keratinizing SCC**.
2. **Imaging for Tumor Staging**:
    - **MRI for soft tissue extent**.
    - **CT scan for bony invasion**.
    - **PET-CT if lymph node involvement or metastasis is suspected**.
3. **Multidisciplinary Oncologic Management**:
    - **Surgical resection with negative margins**.
    - **Neck dissection if nodal metastases are confirmed**.
    - **Adjuvant radiotherapy and/or chemotherapy based on TNM staging**.
4. **HPV Testing if Relevant**:
    - **HPV-positive SCC has a better prognosis** and may influence treatment planning.

Conclusion

This lesion is **highly suspicious for invasive squamous cell carcinoma (SCC) of the tongue**. **Urgent biopsy and imaging are necessary for staging and treatment planning**. Would you like **a detailed TNM staging guide for oral SCC or an oncologic treatment flowchart?**

CASE 79

Reference:

https://www.dentisti-italia.it/img_articoli/239/dscn4436.jpg

Chat-GPT4 answer:

Analysis and Probable Diagnosis

Observations:

1. **Exophytic, lobulated, and erythematous gingival mass**:
   - The lesion is **pedunculated or sessile**, protruding from the **gingival margin**.
   - It appears **hyperplastic and vascularized**, with a **smooth but irregular surface**.
   - There is **no visible ulceration or necrosis**.
2. **Localized Gingival Enlargement with Inflammatory Features**:
   - The lesion appears to originate from the **gingiva and interdental papilla**.
   - **Surrounding gingiva appears inflamed**, suggesting a **reactive or inflammatory origin**.
3. **No Evidence of Bony Involvement or Malignant Features**:
   - The lesion is **confined to soft tissues**.
   - There are **no obvious signs of deep invasion, ulceration, or rolled borders**.

Most Probable Diagnosis: Pyogenic Granuloma (Pregnancy Tumor)

- **Pyogenic granuloma (PG)** is a **benign, vascularized, reactive lesion** that occurs due to **chronic irritation or hormonal influences**.
- **Common features:**
  - **Rapidly growing, vascularized mass**.
  - **Soft, friable, and bleeds easily**.
  - Often occurs in **pregnant women (hormonal influence) or due to chronic irritation**.
- **Etiology**:
  - **Chronic irritation from plaque, calculus, or dental appliances**.
  - **Hormonal changes (pregnancy, puberty, oral contraceptives)**.
  - **Minor trauma (e.g., from brushing or dental procedures)**.

Differential Diagnoses to Consider:

1. **Peripheral Giant Cell Granuloma (PGCG) (If Bluish-Purple and Firm)**
   - PGCG appears **bluish-red**, more **firm**, and may show **bony resorption on radiographs**.
   - Histologically, it contains **multinucleated giant cells**.
2. **Peripheral Ossifying Fibroma (POF) (If Calcified)**
   - POF is **more fibrotic, firm, and slow-growing**.
   - Often involves **mineralized deposits**, requiring **radiographic evaluation**.
3. **Squamous Cell Carcinoma (If Ulcerated and Indurated)**

- If the lesion is **persistent, firm, ulcerated, or rapidly growing**, **biopsy is mandatory** to rule out **oral squamous cell carcinoma (OSCC)**.

4. **Leukemic Gingival Hyperplasia (If Diffuse and Associated with Systemic Symptoms)**
   - If the patient has **pancytopenia, fatigue, or bruising**, consider **hematologic disorders like leukemia**.

Recommended Next Steps:

1. **Thorough Clinical and Radiographic Examination**:
   - Evaluate for **calculus, local irritation, or underlying bone involvement** (periapical X-ray or CBCT if needed).
   - **Rule out systemic conditions (e.g., pregnancy, leukemia, hormonal therapy).**
2. **Excisional Biopsy (If Persistent or Large)**:
   - **If the lesion is symptomatic, rapidly growing, or non-resolving, biopsy is necessary** to confirm diagnosis and exclude malignancy.
3. **Oral Hygiene Optimization**:
   - **Professional scaling and root planing (SRP)** to remove plaque/calculus.
   - **Chlorhexidine mouthwash** (0.12%) for inflammation control.
   - **Meticulous home oral hygiene** to prevent recurrence.
4. **Surgical Excision for Definitive Treatment**:
   - **Complete excision down to periosteum** to prevent recurrence.
   - **Laser excision or electrosurgery** can minimize bleeding due to the vascular nature of the lesion.
5. **Follow-Up and Recurrence Monitoring**:
   - Recurrence is **common if the irritant is not eliminated**.
   - Regular follow-up **every 3–6 months** is recommended.

Conclusion

This lesion is **highly suggestive of pyogenic granuloma**, likely due to **chronic irritation or hormonal factors**. **Initial management includes oral hygiene improvement and, if necessary, surgical excision with histopathologic confirmation**. Would you like **a step-by-step excision protocol or histopathology guidelines for differentiating gingival reactive lesions?**

CASE 80

Reference:

https://marianiumberto.it/wp-content/uploads/2018/09/Angiomi/image13.jpeg

Chat-GPT4 answer:

Analysis and Probable Diagnosis

Observations:

1. **Solitary, well-defined, bluish-purple lesion on the lateral tongue**:
   - The lesion is **soft, non-ulcerated, and dome-shaped**.
   - No signs of **induration, necrosis, or surrounding erythema**.
   - The lesion appears **vascular in nature**.
2. **Superficial location, likely a blood-filled lesion**:
   - The bluish hue and smooth surface suggest **vascular congestion or hemorrhage**.
3. **No Features Suggestive of Malignancy**:
   - The lesion does not show **rapid growth, irregular borders, or ulceration**.

Most Probable Diagnosis: Lingual Hemangioma or Venous Lake

- **Hemangiomas** are **benign vascular proliferations** commonly found on the **lips, tongue, and buccal mucosa**.
- **Venous lakes** are **venous ectasias (dilated venules)** that appear as **bluish-purple papules**.
- **Key features:**
   - **Soft, compressible, and blanches on pressure**.
   - **Slow-growing, often asymptomatic**.
   - More common in **older adults**.

Differential Diagnoses to Consider:

1. **Thrombosed Varix (If Firm and Non-Blanching)**
   - A **thrombosed venous lesion** may appear **firmer and non-compressible**.
   - May **persist longer than a typical hematoma**.
2. **Mucocele (If Fluctuant and Recurrent)**
   - More common in the **lower lip and ventral tongue**.
   - **Clear or bluish**, often **transient and recurrent**.
3. **Melanoma (If Irregular and Growing)**
   - **Atypical pigmentation, irregular borders, and progressive growth** would raise suspicion.
   - If present, **biopsy is mandatory**.
4. **Kaposi Sarcoma (If Multiple and Associated with HIV)**
   - **Purple or red macules that progress into nodules**.
   - **HIV/AIDS history or immunosuppression should raise concern**.

Recommended Next Steps:

1. **Perform Diascopy (Blanching Test)**
    - Apply **pressure with a glass slide**:
        - If it **blanches**, it confirms a **vascular lesion (hemangioma or venous lake)**.
        - If it **does not blanch**, consider **melanoma or thrombosed varix**.
2. **Monitor if Small and Asymptomatic**
    - **Venous lakes and small hemangiomas are benign and do not require intervention**.
3. **Consider Treatment if Symptomatic or for Cosmetic Reasons**
    - **Laser therapy (Nd:YAG or pulsed dye laser)** is effective.
    - **Surgical excision if persistent or concerning**.
4. **Biopsy if Atypical Features Are Present**
    - If the lesion is **firm, non-blanching, growing, or irregular**, a **biopsy is mandatory**.

Conclusion

This lesion is **most consistent with a lingual hemangioma or venous lake**, both **benign vascular lesions**. If **blanching is present, observation is sufficient**. If the lesion **persists, enlarges, or has irregular features, biopsy is necessary**. Would you like **a step-by-step guide for vascular lesion management or a differential table for pigmented oral lesions?**

CASE 81

Reference:

https://www.dentistavomero.it/wp-content/uploads/2023/02/papilloma-squamoso-orale-1024x560.jpg?x47274

Chat-GPT4 answer:

Analysis and Probable Diagnosis

Observations:

1. **Exophytic, white, papillary lesion on the hard palate**:
   - The lesion appears **pedunculated or sessile, with a rough, verrucous surface**.
   - The color is **white, indicating hyperkeratosis or epithelial proliferation**.
2. **No Signs of Ulceration, Induration, or Erythema**:
   - The surrounding mucosa appears **healthy with no significant inflammatory response**.
   - There is **no necrosis, bleeding, or infiltrative appearance**.
3. **Common Location (Hard Palate) for HPV-Associated Lesions**:
   - The **hard palate and gingiva** are common sites for **HPV-related oral lesions**.
   - The lesion's **papillary, cauliflower-like growth suggests a viral or benign epithelial proliferation**.

Most Probable Diagnosis: Squamous Papilloma (HPV-Related)

- **Squamous papillomas** are **benign epithelial proliferations caused by human papillomavirus (HPV), most commonly types 6 and 11**.
- **Key characteristics:**
  - **Slow-growing, asymptomatic, and exophytic with a finger-like or verrucous surface**.
  - **Commonly found on the soft palate, uvula, or buccal mucosa**.
  - **Usually solitary, non-painful, and does not ulcerate unless secondarily traumatized**.

Differential Diagnoses to Consider:

1. **Verruca Vulgaris (If Similar Lesions Are Present on the Hands)**
   - **Wart-like lesion caused by HPV**, typically found in **children or immunocompromised individuals**.
   - **Can be transmitted from hand-to-mouth contact**.
2. **Condyloma Acuminatum (If Multiple Lesions or Anogenital HPV History)**
   - Associated with **HPV types 6 and 11**, similar to squamous papilloma.
   - **Typically larger, multiple, and found in sexually active patients**.
3. **Verrucous Carcinoma (If the Lesion Is Large and Persistent)**
   - **Slow-growing but locally aggressive SCC variant**.
   - If the lesion **continues growing, exhibits deep invasion, or becomes indurated, biopsy is mandatory**.
4. **Focal Epithelial Hyperplasia (Heck's Disease – If Multiple Lesions Are Present)**

- **HPV 13 and 32-associated multiple soft, pink papules**.
- More common in **children and immunocompromised individuals**.

5. **Keratoacanthoma (If Rapid Growth Over a Few Weeks)**
    - A rare **rapidly growing epithelial lesion** that may regress spontaneously.
    - Biopsy is necessary for confirmation.

Recommended Next Steps:

1. **Complete Excisional Biopsy for Histopathological Diagnosis**:
    - **Gold standard for diagnosis**.
    - Ensures **HPV typing and exclusion of verrucous carcinoma**.
2. **Assess for Risk Factors (HPV Exposure, Sexual History, Immunosuppression)**
    - If the patient is immunocompromised or has a history of **recurrent warts**, further **HPV screening may be considered**.
3. **Monitor for Recurrence**:
    - **Squamous papillomas rarely recur after excision**.
    - If multiple or persistent, consider **HPV-related immunosuppression**.
4. **Patient Education on HPV and Oral Hygiene**:
    - HPV-related lesions are **benign but contagious through direct contact**.
    - Good oral hygiene reduces irritation and secondary trauma.

Conclusion

This lesion is **most consistent with squamous papilloma, a benign HPV-induced epithelial proliferation**. **Excisional biopsy is recommended to confirm diagnosis and exclude malignancy**. Would you like **a surgical excision guide or HPV-related oral lesion management protocol?**

CASE 82

Reference:

https://media.springernature.com/lw685/springer-static/image/chp%3A10.1007%2F978-3-030-91920-7_34/MediaObjects/470211_4_En_34_Fig1_HTML.jpg

Chat-GPT4 answer:

Analysis and Probable Diagnosis

Observations:

1. **Irregular, exophytic, ulcerative mass on the retromolar trigone and posterior buccal mucosa**:
    - The lesion appears **lobulated, friable, and proliferative**.
    - There is **surface erosion, granulation, and evidence of necrosis**.
    - The surrounding mucosa shows **erythema and possible tissue induration**.
2. **Deep Infiltrative Pattern**:
    - The lesion appears **deeply seated and possibly invading adjacent structures**.
    - There may be **involvement of the pterygomandibular raphe, soft palate, or tongue base**.
3. **High-Risk Location (Retromolar Trigone, Posterior Buccal Mucosa)**:
    - The **retromolar trigone is a high-risk site for malignant transformation**.
    - **Tumors in this region often present late due to minimal early symptoms**.

Most Probable Diagnosis: Squamous Cell Carcinoma (SCC) of the Buccal Mucosa/Retromolar Trigone

- **Oral squamous cell carcinoma (OSCC) is the most common malignancy in this region**.
- **Key features raising suspicion for SCC**:
    - **Exophytic and ulcerative growth with irregular borders**.
    - **Friability and necrosis, suggesting rapid tissue breakdown**.
    - **Potential deep infiltration and fixation to underlying structures**.
- **Risk Factors for SCC**:
    - **Tobacco and betel nut chewing (strongest association for buccal SCC)**.
    - **Chronic alcohol use (synergistic effect with tobacco)**.
    - **HPV-related carcinogenesis (less common in this site)**.
    - **Poor oral hygiene and chronic irritation**.

Differential Diagnoses to Consider:

1. **Verrucous Carcinoma (If Slowly Growing and Less Invasive)**
    - A **low-grade variant of SCC**.
    - Typically presents as a **verrucous (warty) exophytic lesion without deep invasion**.
2. **Necrotizing Sialometaplasia (If Non-Neoplastic and Self-Limiting)**
    - A benign, **self-healing ischemic necrosis of minor salivary glands**.
    - Mimics **SCC but lacks progressive growth or deep invasion**.

3. **Chronic Traumatic Ulcer or Inflammatory Pseudotumor**
    - May arise due to **irritation from sharp teeth or prostheses**.
    - Typically **resolves once the irritant is removed**.
4. **Deep Fungal Infection (If Immunocompromised)**
    - **Histoplasmosis, blastomycosis, or paracoccidioidomycosis** can mimic SCC.
    - Requires **biopsy and fungal stains (PAS, GMS)**.
5. **Tuberculous Ulcer (If Systemic Symptoms Present)**
    - **Chronic, non-healing ulcers with undermined edges**.
    - Requires **TB testing and biopsy with AFB staining**.

Recommended Next Steps:

1. **Urgent Incisional Biopsy for Histopathologic Confirmation**:
    - **H&E staining for cellular atypia, keratinization, and invasion pattern**.
    - **p16 immunohistochemistry for HPV status (if relevant)**.
2. **Imaging for Tumor Staging**:
    - **MRI for soft tissue invasion**.
    - **CT scan for bony involvement**.
    - **PET-CT if nodal or distant metastasis is suspected**.
3. **Multidisciplinary Management**:
    - **Surgical resection with negative margins (wide local excision)**.
    - **Neck dissection if nodal metastases are present**.
    - **Adjuvant radiotherapy/chemotherapy for advanced-stage disease**.

Conclusion

This lesion is **highly suspicious for invasive squamous cell carcinoma (SCC) of the retromolar trigone and posterior buccal mucosa. An urgent biopsy is necessary to confirm malignancy and guide treatment planning**. Would you like **a TNM staging guide or a treatment algorithm for oral SCC?**

CASE 83

Reference:

https://cdnintech.com/media/chapter/71159/1701683619/media/F3.png

Chat-GPT4 answer:

Analysis and Probable Diagnosis

Observations:

1. **Large, ulcerated, exophytic mass involving the tongue**:
    - The lesion is **irregular, lobulated, and infiltrative**.
    - It shows **areas of ulceration, necrosis, and hemorrhage**.
    - The surface appears **heterogeneous, with keratotic and necrotic regions**.
2. **Extension to the Floor of the Mouth and Possible Infiltration into Adjacent Structures**:
    - The lesion appears to **displace the tongue and extend posteriorly**.
    - Possible **invasion into the lateral or ventral tongue and floor of the mouth**.
3. **Severe Dental Plaque and Poor Oral Hygiene**:
    - Suggests a **chronic condition or neglect**, often associated with malignancies.

Most Probable Diagnosis: Advanced Squamous Cell Carcinoma (SCC) of the Tongue

- **Oral squamous cell carcinoma (OSCC)** is the most common malignancy of the oral cavity.
- **Key Risk Factors**:
    - **Tobacco and alcohol consumption** (synergistic effect).
    - **HPV infection (especially HPV-16, HPV-18)**.
    - **Poor oral hygiene and chronic irritation**.
    - **Betel quid chewing in endemic regions**.
- **Clinical Features Supporting SCC**:
    - **Exophytic, ulcerated, and infiltrative lesion**.
    - **Non-healing, indurated, and rapidly enlarging mass**.
    - **Irregular surface with keratinization and necrosis**.
    - **Possible regional lymph node metastasis (requires palpation of cervical nodes)**.

Differential Diagnoses to Consider:

1. **Verrucous Carcinoma (If Slow-Growing and Wart-Like)**
    - A **low-grade variant of SCC** with **pushing rather than infiltrative margins**.
    - Less likely in this case due to **ulceration and necrosis**.
2. **Deep Fungal Infection (Histoplasmosis, Blastomycosis)**
    - Some chronic fungal infections can mimic malignancies.
    - **Biopsy with PAS and GMS staining is required for confirmation**.
3. **Tuberculous Ulcer (If History of Pulmonary TB)**
    - May present as a **chronic, non-healing ulcer**.
    - Requires **TB serology and biopsy** for confirmation.
4. **Syphilitic Gumma (If Risk Factors for Syphilis Exist)**

- Chronic, **painless ulcers with indurated borders**.
- Confirmed with **serologic testing (VDRL, FTA-ABS)**.

Urgent Recommended Next Steps:

1. **Immediate Incisional Biopsy**
    - **Histopathologic confirmation is mandatory** to rule out high-grade SCC.
    - Immunohistochemistry (IHC) with **p16 for HPV-related SCC** can be performed.
2. **Imaging (CT or MRI)**
    - Evaluate **tumor invasion into deep structures (muscles, bone, floor of the mouth)**.
    - Assess for **regional lymph node involvement**.
3. **Cervical Lymph Node Assessment**
    - Palpation of **levels I–III for nodal metastasis**.
    - Consider **ultrasound-guided fine-needle aspiration (FNA) if nodal involvement is suspected**.
4. **Oncology Referral for Staging and Treatment Planning**
    - **Treatment options based on staging**:
        - **Early-stage SCC (T1–T2, N0): Surgery ± radiotherapy**.
        - **Locally advanced SCC (T3–T4, N+): Surgery + adjuvant chemoradiotherapy**.
        - **Metastatic SCC: Palliative chemoradiotherapy (Cisplatin, Cetuximab, or PD-1 inhibitors)**.
5. **Comprehensive Risk Factor Assessment**
    - **HPV testing** if SCC is suspected.
    - **Smoking cessation and alcohol cessation counseling**.

Conclusion

This lesion is **highly suspicious for advanced squamous cell carcinoma (SCC) of the tongue**, given its **ulcerated, exophytic, and infiltrative appearance**. **Urgent biopsy and imaging are required for staging and treatment planning**. Would you like **TNM staging guidelines or oncologic management protocols?**

CASE 84

Reference:

https://i0.wp.com/www.centridentisticiprimo.it/wp-content/uploads/2021/04/afta-quando-preoccuparsi-e-come-curarla.jpg?ssl=1

Chat-GPT4 answer:

Analysis and Probable Diagnosis

Observations:

1. **Well-defined, round ulcerations with a white fibrinous center and erythematous halo**:
   - The lesions are **small, shallow, and localized on the inner labial mucosa**.
   - The surrounding mucosa appears **erythematous but intact**.
2. **No Induration, No Exophytic Growth, and No Vesicles**:
   - The ulcers are **not raised or infiltrative**, reducing concern for malignancy.
   - **No vesicular stage observed**, which is a key feature distinguishing aphthous ulcers from herpes.
3. **Painful and Self-Limited**:
   - Patients often report **burning or tingling before ulceration**.
   - Typically **resolves within 7–14 days without scarring**.

Most Probable Diagnosis: Recurrent Aphthous Stomatitis (RAS)

- Also known as **canker sores**, RAS is a **common, idiopathic, ulcerative disorder of the oral mucosa**.
- **Key features:**
   - **Painful, shallow ulcers with an erythematous border**.
   - **No viral or bacterial cause identified**.
   - **Self-limiting, usually healing within 1–2 weeks**.
- **Etiology and Predisposing Factors**:
   - **Stress, trauma, hormonal changes, and nutritional deficiencies (B12, folate, iron, zinc)**.
   - **Immune dysfunction or systemic conditions (Behçet's disease, inflammatory bowel disease, celiac disease).**

Differential Diagnoses to Consider:

1. **Herpetic Gingivostomatitis (If Vesicular and Clustered)**
   - **HSV-1 presents as vesicles that rupture into ulcers**.
   - Lesions are **more clustered and commonly involve attached gingiva**.
2. **Traumatic Ulcers (If Associated with Mechanical Injury)**
   - **History of lip biting, sharp teeth, or dental work**.
   - **Single lesion**, heals once irritation is removed.
3. **Oral Lichen Planus (If Chronic and Reticular)**
   - **Reticular or erosive white striae (Wickham's striae)** on the buccal mucosa.

    - More **chronic and widespread**.
4. **Behçet's Disease (If Systemic Symptoms Are Present)**
    - **Multiple oral ulcers with genital ulcers and ocular involvement**.
    - Consider if ulcers are **frequent, large, or non-healing**.

Recommended Next Steps:

1. **Symptomatic Management**:
    - **Topical corticosteroids (clobetasol gel, triamcinolone paste)** to reduce inflammation.
    - **Analgesic mouthwashes (benzydamine or lidocaine)** for pain relief.
    - **Avoid spicy, acidic, or rough foods** that may worsen irritation.
2. **Evaluate for Nutritional Deficiencies or Underlying Conditions**:
    - **Check B12, folate, iron, and zinc levels** in recurrent cases.
    - Consider systemic conditions like **celiac disease or inflammatory bowel disease** if ulcers are frequent.
3. **Monitor for Recurrence and Severity**:
    - If lesions **persist beyond 2 weeks, biopsy may be needed** to rule out other etiologies.
    - **Systemic immunomodulators (colchicine, thalidomide) for severe or major aphthous ulcers**.

Conclusion

This lesion is **highly suggestive of minor recurrent aphthous stomatitis**, a **self-limiting inflammatory condition**. **Symptomatic relief with topical steroids and supportive care is usually sufficient**. Would you like **a detailed treatment protocol for RAS or a differential diagnosis chart for oral ulcers?**

CASE 85

Reference:

https://www.scielo.cl/fbpe/img/ijodontos/v10n3/art13_f1.jpg

Chat-GPT4 answer:

Analysis and Probable Diagnosis

Observations:

1. **Multiple well-defined, round, shallow ulcers with erythematous halos**:
   - The lesions appear **painful, oval, and covered by a yellowish-white pseudomembrane**.
   - The surrounding mucosa is **inflamed and erythematous**.
   - The ulcers are located on the **ventral and lateral tongue**, common sites for non-keratinized mucosal lesions.
2. **No Evidence of Vesicles or Extensive Involvement**:
   - The absence of **vesicles or widespread mucosal involvement** makes **herpetic stomatitis or pemphigoid less likely**.
3. **No Induration, Exophytic Growth, or Pigmentation**:
   - This reduces suspicion for **malignant or premalignant lesions**.

Most Probable Diagnosis: Recurrent Aphthous Stomatitis (RAS)

- **Recurrent aphthous ulcers (canker sores)** are the most common **non-infectious oral ulcerative condition**.
- **Key characteristics:**
  - **Round or oval ulcers with erythematous borders**.
  - **Painful, recurrent, and self-limiting**.
  - Typically found on **non-keratinized mucosa (tongue, buccal mucosa, labial mucosa)**.

Differential Diagnoses to Consider:

1. **Herpetic Stomatitis (If Multiple Small Vesicles Preceded Ulcers)**
   - **Primary herpes simplex virus (HSV-1) presents with vesicles that rupture to form ulcers**.
   - More common on **keratinized mucosa (gingiva, palate)**.
   - **Systemic symptoms (fever, malaise)** are often present.
2. **Behçet's Syndrome (If Systemic Symptoms Are Present)**
   - **Recurrent aphthous ulcers + genital ulcers + ocular involvement**.
   - Consider **if ulcers are deep, large, or associated with vasculitis**.
3. **Oral Lichen Planus (If Reticular or Chronic Ulcers Present)**
   - **Chronic, bilateral ulcers with Wickham's striae (white lacy patterns)**.
   - Typically found on **buccal mucosa**.
4. **Erythema Multiforme (If Target Lesions on Skin Present)**
   - Acute **immune-mediated ulcerative lesions** triggered by drugs or infections.

- Associated with **skin target lesions and systemic involvement**.

Recommended Next Steps:

1. **Symptomatic Management (If Diagnosis is Confirmed)**
   - **Topical corticosteroids (e.g., Clobetasol, Fluocinonide gel)**.
   - **Topical analgesics (e.g., benzocaine, lidocaine)**.
   - **Chlorhexidine mouth rinses for secondary infection prevention**.
2. **Identify Possible Triggers**
   - **Recent stress, trauma, food sensitivities, or nutritional deficiencies (iron, B12, folate)?**
   - **Ruling out systemic causes (e.g., celiac disease, Behçet's, HIV, neutropenia)** if ulcers are recurrent.
3. **Biopsy if Lesions Are Persistent or Atypical**
   - If ulcers **persist beyond 2 weeks, are increasing in size, or recur excessively**, biopsy should be considered.

Conclusion

This presentation is **highly suggestive of recurrent aphthous stomatitis (RAS)**. **Symptomatic relief with topical corticosteroids and analgesics is recommended**. If ulcers **persist beyond 2 weeks or are unusually large, a biopsy is necessary**. Would you like **a treatment protocol for aphthous ulcers or a differential diagnosis guide for oral ulcerations?**

CASE 86

Reference:

https://www.pcds.org.uk/imager/gallery/clinical/mucocoele/12503/muc_tongue_fee391183f15cb4d62773032fe0be92d.jpg

Chat-GPT4 answer:

Analysis and Probable Diagnosis

Observations:

1. **Small, well-defined, fluid-filled lesion on the lateral border of the tongue**:
   - The lesion appears **smooth, dome-shaped, and translucent**.
   - The **color is slightly bluish or pinkish**, suggesting a **fluid-filled nature**.
   - No signs of **ulceration, induration, or inflammation**.
2. **No Signs of Malignancy or Infiltration**:
   - The lesion is **not nodular, exophytic, or ulcerated**.
   - It does not exhibit **necrosis, irregular borders, or fixation to deeper tissues**.
3. **Common Location for Salivary or Traumatic Lesions**:
   - The **lateral tongue** is frequently affected by **trauma, salivary gland obstruction, or reactive growths**.

Most Probable Diagnosis: Mucocele (Mucous Extravasation Phenomenon)

- **Mucoceles** are **benign, self-limiting salivary gland lesions** caused by **trauma-induced rupture of minor salivary gland ducts**, leading to **extravasation and accumulation of mucin**.
- **Key characteristics:**
  - **Soft, painless, fluid-filled, and bluish-pink in color**.
  - **Common sites**: lower lip, ventral tongue, buccal mucosa.
  - **History of repetitive trauma or lip/tongue biting** is common.
  - **May spontaneously rupture and recur**.

Differential Diagnoses to Consider:

1. **Traumatic Fibroma (If Firm and Persistent)**
   - Chronic irritation can cause a **firm, fibrotic, and non-fluctuant lesion**.
   - Lacks the **fluid-filled, translucent appearance of a mucocele**.
2. **Squamous Papilloma (If Verrucous or Exophytic)**
   - HPV-related lesions are typically **papillary, rough, or cauliflower-like**.
   - Unlike mucoceles, **they do not fluctuate or rupture**.
3. **Vascular Lesion (If Bluish and Compressible)**
   - **Hemangiomas** or **venous malformations** may appear **blue and compressible**.
   - Unlike mucoceles, **they blanch with pressure**.
4. **Minor Salivary Gland Tumor (If Persistent or Growing)**
   - Low-grade **mucoepidermoid carcinoma** can mimic mucoceles.
   - If the lesion **persists beyond 3 months or recurs frequently**, a **biopsy is required**.

Recommended Next Steps:

1. **Monitor for Spontaneous Resolution**:
   - Many mucoceles **spontaneously rupture and resolve within weeks**.
   - If the lesion **persists or recurs**, further evaluation is warranted.
2. **Avoid Trauma or Habitual Biting**:
   - Repetitive biting can lead to **recurrence and chronicity**.
   - Patient should be educated on **avoiding habitual trauma**.
3. **Consider Surgical Excision if Persistent**:
   - If the lesion **does not resolve within 6–8 weeks or recurs**, **surgical excision with removal of involved minor salivary glands** is recommended.
   - Excision prevents **recurrence and ensures histopathological evaluation**.
4. **Biopsy if the Lesion Is Atypical or Growing**:
   - If the lesion **becomes firm, enlarges, or does not resolve**, a **biopsy is necessary to rule out salivary gland neoplasms**.

Conclusion

This lesion is **most consistent with a mucocele**, a **benign, self-limiting salivary gland lesion**. **Observation is advised**, but if it **persists, recurs, or enlarges, surgical excision and biopsy should be considered**. Would you like **a surgical excision guide or a mucocele management protocol?**

CASE 87

Reference:

https://www.researchgate.net/publication/6287737/figure/fig1/AS:601626641453059@1520450377211/T2-squamous-cell-carcinoma-on-the-ventral-tongue.png

Chat-GPT4 answer:

Analysis and Probable Diagnosis

Observations:

1. **Large, exophytic, ulcerated mass on the posterior tongue and oropharyngeal region**:
   - The lesion appears **irregular, lobulated, and infiltrative**.
   - The surface shows **keratinized white patches, ulcerations, and necrotic areas**.
   - Surrounding mucosa is **erythematous and inflamed**.
2. **Possible Extension into the Oropharynx and Lateral Pharyngeal Wall**:
   - The lesion seems to involve the **posterior tongue**, possibly infiltrating the **oropharynx**.
   - High suspicion for **deep tissue invasion and airway compromise**.
3. **No Clear Evidence of Acute Infection (Abscess) but High Suspicion of Malignancy**:
   - The mass appears **solid rather than fluctuating**, ruling out a simple infectious process.
   - Features are **highly suggestive of a neoplastic process**.

Most Probable Diagnosis: Advanced Squamous Cell Carcinoma (SCC) of the Tongue and Oropharynx

- **Oral and oropharyngeal SCC is the most common malignancy of this region**.
- **High-risk factors**:
  - **Tobacco and alcohol use** (synergistic effect).
  - **HPV-associated oropharyngeal carcinoma (HPV-16, HPV-18)**.
  - **Chronic irritation and poor oral hygiene**.
- **Key Clinical Features Supporting SCC**:
  - **Exophytic and ulcerated appearance**.
  - **Firm, indurated mass with keratotic areas**.
  - **Rapidly growing and infiltrative nature**.
  - **Possible dysphagia, odynophagia, or airway obstruction**.

Differential Diagnoses to Consider:

1. **Verrucous Carcinoma (If Slow-Growing and Papillary)**
   - A **low-grade SCC variant**, typically **pushing rather than infiltrative**.
   - Less likely due to **ulceration and necrotic appearance**.
2. **Deep Fungal Infection (Histoplasmosis, Blastomycosis)**
   - Chronic fungal infections can **mimic carcinoma**.
   - **Biopsy with PAS and GMS staining can differentiate fungal infections**.

3. **Tuberculous Granuloma (If Chronic, Non-Healing, and Associated with TB)**
    - If the patient has a **history of pulmonary TB**, this should be considered.
    - Requires **biopsy and AFB staining**.
4. **Syphilitic Gumma (If Painless and Indurated)**
    - Presents as a **chronic, non-healing ulcer**.
    - **Serologic testing (VDRL, FTA-ABS) is needed**.

Urgent Recommended Next Steps:

1. **Immediate Incisional Biopsy for Histopathologic Confirmation**
    - **Mandatory to confirm SCC and assess for HPV-related etiology (p16 immunostaining).**
    - **Histological markers:** Keratin pearls, dysplasia, invasive carcinoma.
2. **Imaging for Tumor Staging**
    - **MRI or CT scan** to assess **tumor depth and spread**.
    - **PET-CT** if metastatic disease is suspected.
3. **Cervical Lymph Node Examination**
    - **Palpation of levels I–IV for nodal metastases**.
    - **Ultrasound-guided fine-needle aspiration (FNA) for enlarged lymph nodes**.
4. **Multidisciplinary Oncology Consultation**
    - **Treatment plan based on TNM staging**:
        - **Early-stage (T1-T2, N0)** → Surgery ± Radiotherapy.
        - **Locally advanced (T3-T4, N+)** → Surgery + Adjuvant Chemoradiotherapy.
        - **Metastatic (M1, N3)** → Palliative Chemotherapy (Cisplatin, Cetuximab, or PD-1 inhibitors).
5. **HPV Testing and Risk Factor Modification**
    - **HPV-positive SCC has a better prognosis**.
    - **Smoking cessation and alcohol cessation counseling**.

Conclusion

This lesion is **highly suspicious for advanced oropharyngeal squamous cell carcinoma (SCC)**. **Urgent biopsy, imaging, and oncology referral are required for staging and treatment planning**. Would you like **TNM staging guidelines or a management protocol for advanced SCC?**

CASE 88

Reference:

https://marianiumberto.it/wp-content/uploads/2018/09/Mucocele/mucocele16.jpeg

Chat-GPT4 answer:

Analysis and Probable Diagnosis

Observations:

1. **Well-circumscribed, soft, dome-shaped swelling on the floor of the mouth**:
   - The lesion appears **smooth, translucent, and fluctuant**.
   - It is **red to pink in color**, indicating vascularization.
   - The mucosa covering the lesion is **intact, with no ulceration or induration**.
2. **Submucosal Location with Possible Fluid Content**:
   - The swelling appears **fluid-filled, likely originating from a salivary gland**.
   - There is **no obvious sign of infection, malignancy, or inflammatory changes**.
3. **Common Location for Salivary Gland Disorders**:
   - The **floor of the mouth is a frequent site for salivary gland cysts or ductal obstructions**.

Most Probable Diagnosis: Ranula (Oral Mucous Retention Cyst)

- **Ranulas are mucoceles (mucous retention cysts) of the sublingual gland**.
- **Key characteristics**:
  - **Soft, painless, fluctuant swelling in the floor of the mouth**.
  - **May be unilateral or midline**.
  - **May enlarge over time and interfere with speech or swallowing**.
  - **Often caused by trauma or obstruction of the sublingual duct**.

Differential Diagnoses to Consider:

1. **Plunging (Cervical) Ranula (If Extending into the Neck)**
   - A ranula that **extends through the mylohyoid muscle into the neck**.
   - Presents as a **submandibular swelling** in addition to the intraoral lesion.
2. **Dermoid or Epidermoid Cyst (If Midline and Doughy Consistency)**
   - More common in **children and young adults**.
   - **Firm, non-fluctuant, and slow-growing**.
3. **Salivary Gland Neoplasm (If Firm and Progressive)**
   - **Mucoepidermoid carcinoma is a primary concern if the lesion is firm and persistent**.
   - Requires **biopsy if growth is rapid or induration is present**.
4. **Lymphangioma (If Present Since Childhood)**
   - Presents as **soft, compressible, and translucent vesicular swellings**.
   - Typically congenital.

Recommended Next Steps:

1. **Clinical Monitoring if Small and Asymptomatic**:
    - **If the lesion is small and not causing functional impairment**, observation may be sufficient.
2. **Aspiration or Marsupialization for Diagnosis and Treatment**:
    - **Aspiration of mucous confirms the diagnosis**.
    - **Marsupialization (unroofing) is the preferred treatment for smaller ranulas**.
3. **Surgical Excision if Persistent or Large**:
    - **Excision of the ranula with removal of the affected sublingual gland** prevents recurrence.
    - If a **plunging ranula is suspected, a cervical approach may be required**.
4. **Imaging (Ultrasound or MRI) if a Plunging Component is Suspected**:
    - **Ultrasound**: Confirms a cystic nature.
    - **MRI**: Useful for detecting **deep extension into the neck**.

Conclusion

This lesion is **most consistent with a ranula (mucous retention cyst of the sublingual gland)**. **Small, asymptomatic ranulas can be monitored, but larger or persistent lesions require marsupialization or excision**. Would you like **a surgical guide for ranula excision or a differential table for sublingual swellings?**

CASE 89

Reference:

https://dccdn.de/www.doccheck.com/data/ek/2f/sd/42/q6/v7/gingivahyperplasie-uk-front-vertikalansicht_lg.jpg

Chat-GPT4 answer:

Analysis and Probable Diagnosis

Observations:

1. **Generalized gingival enlargement (hyperplasia)**:
   - The gingiva appears **swollen, firm, and erythematous**.
   - It extends over a significant portion of the crowns of the **mandibular anterior teeth**.
   - The overgrowth appears **fibrotic in some areas and inflamed in others**.
2. **Presence of Orthodontic Retainer**:
   - The **fixed lingual retainer** is visible, suggesting **previous orthodontic treatment**.
   - **Plaque accumulation and irritation from the retainer may contribute to the inflammation**.
3. **Signs of Gingivitis and Early Periodontal Involvement**:
   - **Localized bleeding and erythema** suggest **inflammatory gingival enlargement**.
   - Possible **localized gingival recession at the lower central incisors**.

Most Probable Diagnosis: Drug-Induced or Plaque-Induced Gingival Hyperplasia

- **Gingival hyperplasia can have multiple etiologies, but in this case, the most likely causes include**:
  1. **Plaque-Induced Gingival Hyperplasia**:
     - **Orthodontic appliances can increase plaque accumulation**, leading to **chronic irritation**.
     - If **hygiene is compromised**, **localized inflammatory gingival overgrowth** can develop.
  2. **Drug-Induced Gingival Hyperplasia (If the Patient Uses Certain Medications)**:
     - **Common causative drugs**:
       - **Calcium channel blockers** (e.g., nifedipine, amlodipine).
       - **Anticonvulsants** (e.g., phenytoin).
       - **Immunosuppressants** (e.g., cyclosporine).
     - **Drug-induced hyperplasia is fibrotic, firm, and non-tender**.
  3. **Hereditary or Idiopathic Gingival Fibromatosis (Less Likely but Possible)**
     - A **rare genetic condition** that leads to **generalized fibrotic gingival overgrowth**.
     - Usually **presents in childhood or adolescence**.

Differential Diagnoses to Consider:

1. **Leukemic Gingival Infiltration (If Systemic Symptoms Present)**

- Leukemia can cause **diffuse gingival enlargement with spontaneous bleeding**.
- **If systemic symptoms (fatigue, weight loss, easy bruising) are present, hematologic evaluation is needed**.

2. **Hormonal Gingival Hyperplasia (If Adolescent or Pregnant)**
    - **Puberty and pregnancy-related hormonal changes** can exacerbate gingival inflammation.
    - Often **resolves postpartum or with hormonal stabilization**.
3. **Granulomatous Gingivitis (If Associated with Systemic Diseases)**
    - Can be seen in **Crohn's disease or Wegener's granulomatosis**.

Recommended Next Steps:

1. **Professional Debridement and Oral Hygiene Reinforcement**:
    - **Scaling and root planing (SRP) to remove plaque and calculus**.
    - **Antiseptic mouth rinses (chlorhexidine 0.12%)** to control inflammation.
2. **Evaluation for Medication-Induced Hyperplasia**:
    - **Review medical history for drugs associated with gingival overgrowth**.
    - **If drug-induced, consider alternative medications in consultation with the physician**.
3. **Histopathologic Examination (If Fibrotic and Persistent)**
    - **If the overgrowth does not resolve with hygiene improvements**, biopsy should be considered.
4. **Surgical Gingivectomy (If Severe and Persistent)**
    - If the gingival enlargement **persists after plaque control**, **surgical excision (gingivectomy or gingivoplasty) is recommended**.
    - **Laser gingivectomy may be an option for less invasive removal**.
5. **Monitoring for Systemic Conditions**
    - If the patient has **systemic symptoms (fatigue, bruising, weight loss)**, consider **CBC testing to rule out hematologic disorders**.

Conclusion

This case is **highly suggestive of plaque-induced or drug-induced gingival hyperplasia, exacerbated by orthodontic retention. Initial management should focus on professional cleaning, improved oral hygiene, and assessment for contributing medications**. If the overgrowth persists, **biopsy and gingivectomy should be considered**. Would you like **a treatment protocol for gingival overgrowth or a surgical approach guide?**

CASE 90

Reference:

https://www.nejm.org/cms/10.1056/NEJMicm1013092/asset/acc471c9-41da-4b48-b1b3-f1636752bc98/assets/images/large/nejmicm1013092_f1.jpg

Chat-GPT4 answer:

Analysis and Probable Diagnosis

Observations:

1. **Large, fluid-filled, translucent lesion on the ventral tongue**:
   - The lesion appears **bluish, dome-shaped, and well-circumscribed**.
   - The surface is **smooth, thin, and stretched, suggesting fluid accumulation**.
   - The lesion is **fluctuant**, indicating a **cystic or vascular origin**.
2. **No Ulceration, Induration, or Irregular Borders**:
   - The surrounding tissue appears **normal without signs of infection or malignancy**.
   - There is **no erythema, necrosis, or deep invasion**.
3. **Common Location for a Ranula or Mucous Cyst**:
   - The **ventral tongue and floor of the mouth** are typical sites for **salivary gland-related lesions**.

Most Probable Diagnosis: Ranula (Oral or Plunging)

- **Ranulas** are **mucoceles that occur in the floor of the mouth, arising from the sublingual or submandibular salivary glands**.
- **Key characteristics:**
  - **Painless, slow-growing, and fluid-filled**.
  - **Translucent and bluish** in appearance due to mucin accumulation.
  - Can be **simple (confined to the floor of the mouth) or plunging (extending into the neck)**.
  - Often caused by **salivary duct obstruction or trauma**.

Differential Diagnoses to Consider:

1. **Mucocele (If Smaller and More Superficial)**
   - **Similar etiology but usually on the lips or minor salivary glands**.
   - **Mucoceles are smaller and more common than ranulas**.
2. **Hemangioma or Lymphangioma (If Deeply Vascular)**
   - **Bluish, compressible, and may blanch on pressure**.
   - **Doppler ultrasound can differentiate vascular malformations**.
3. **Dermoid or Epidermoid Cyst (If Non-Fluctuant)**
   - These cysts are **firmer, submucosal, and non-translucent**.
4. **Salivary Gland Neoplasm (If Firm and Persistent)**
   - If the lesion **persists despite drainage**, a **low-grade salivary gland tumor should be ruled out**.

Recommended Next Steps:

1. **Confirm Diagnosis with Imaging (Ultrasound or MRI)**
   - **Ultrasound**: Can confirm a **fluid-filled lesion** with no vascularity.
   - **MRI**: Helps differentiate **plunging ranulas extending into the neck**.
2. **Aspiration (If Needed for Diagnosis)**
   - **Fine-needle aspiration (FNA)** can confirm the presence of **mucin**.
3. **Definitive Treatment: Surgical Excision**
   - **Marsupialization (for small, simple ranulas)**.
   - **Complete excision of the ranula and involved sublingual gland (to prevent recurrence)**.
   - **Plunging ranulas require more extensive surgical management**.
4. **Observation if Asymptomatic**
   - Small, **non-enlarging ranulas may not require immediate intervention**.
   - If the lesion **grows, interferes with speech or swallowing, or causes discomfort, surgical excision is indicated**.

Conclusion

This lesion is **most consistent with an oral ranula**, a **salivary gland mucous cyst occurring in the floor of the mouth due to ductal obstruction or rupture. Ultrasound or MRI is recommended to confirm the diagnosis, and definitive treatment involves surgical excision to prevent recurrence**. Would you like **a surgical excision guide or ranula management protocol?**

CASE 91

Reference:

https://assets.nhs.uk/nhsuk-cms/images/S_1217_Leukoplakia_C0235436.original.width-320.jpg

Chat-GPT4 answer:

Analysis and Probable Diagnosis

Observations:

1. **White, slightly raised, irregular plaque on the buccal mucosa near the lower molars**:
   - The lesion appears **non-homogeneous with a rough surface**.
   - No ulceration, but some areas appear **thicker and keratotic**.
2. **Localized to the Buccal Mucosa Along the Occlusal Line**:
   - This is a **common site for frictional or reactive lesions**.
   - No signs of **erythema, nodularity, or exophytic growth**.
3. **No Obvious Signs of Malignancy or Dysplasia**:
   - The lesion does not show **irregular borders, induration, or rapid growth**.

Most Probable Diagnosis: Frictional Keratosis (Chronic Irritation)

- **Frictional keratosis** is a **benign, reactive hyperkeratotic lesion** due to **chronic mechanical irritation**.
- **Key characteristics:**
  - **White plaque-like lesion that cannot be wiped off**.
  - **Commonly found along the occlusal plane due to chronic cheek biting (morsicatio buccarum) or sharp teeth**.
  - Typically **asymptomatic** and does not progress to malignancy.

Differential Diagnoses to Consider:

1. **Leukoplakia (If Persistent Without Obvious Trauma)**
   - **Leukoplakia is a potentially malignant disorder (PMD)**.
   - It presents as a **persistent white plaque with no identifiable cause**.
   - **Requires biopsy if persists beyond 2 weeks**.
2. **Lichen Planus (If Bilateral and Reticular)**
   - **White striations (Wickham's striae) with possible ulcerations**.
   - Often **bilateral and chronic**.
3. **Oral Hairy Leukoplakia (If in Immunocompromised Patients)**
   - **Corrugated, vertical white plaques on the lateral tongue**.
   - Associated with **Epstein-Barr Virus (EBV) and HIV**.
4. **Oral Candidiasis (If Wipeable and Associated with Immunosuppression)**
   - **White patches that can be wiped away, leaving an erythematous base**.
   - Associated with **antibiotic use, diabetes, or immunosuppression**.

Recommended Next Steps:

1. **Identify and Remove the Cause of Friction**
    - **Sharp restorations, rough tooth edges, or chronic cheek biting?**
    - If trauma is present, **removing the source should resolve the lesion**.
2. **Monitor for Changes**
    - If the lesion persists beyond **2-3 weeks after eliminating the cause**, **biopsy is required** to rule out **leukoplakia or dysplasia**.
3. **Consider Biopsy if Any Concerning Features Develop**
    - **Persistent white lesions that do not resolve should be biopsied**.
    - If the lesion **becomes nodular, ulcerated, or enlarges**, consider a **biopsy for dysplasia or carcinoma screening**.

Conclusion

This lesion is **most consistent with frictional keratosis due to chronic irritation**. If the **irritating factor is removed and the lesion resolves, no further action is needed**. If the lesion persists beyond **2-3 weeks or has atypical features, a biopsy is warranted**. Would you like **a management flowchart for white oral lesions or biopsy guidelines for suspicious leukoplakias?**

CASE 92

Reference:

https://media.springernature.com/m685/springer-static/image/art%3A10.1038%2Fs41415-021-3272-9/MediaObjects/41415_2021_3272_Fig1_HTML.jpg

Chat-GPT4 answer:

Analysis and Probable Diagnosis

Observations:

1. **Large, necrotic, ulcerated lesion on the lower vestibular mucosa**:
   - **Yellowish-white pseudomembrane covering the lesion**.
   - **Irregular and indurated borders**, suggesting tissue destruction.
   - Surrounding mucosa appears **erythematous and inflamed**.
2. **Severe Periodontal Disease and Poor Oral Hygiene**:
   - The teeth exhibit **significant plaque, calculus, and discoloration**.
   - There may be **associated periodontal infection**, increasing the risk of necrosis.
3. **No Clear Signs of Vesicles or Fungal Overgrowth**:
   - The lesion is **not vesicular**, ruling out herpetic stomatitis.
   - The **necrotic center and inflammatory border** suggest **bacterial involvement**.

Most Probable Diagnosis: Necrotizing Ulcerative Stomatitis (NUS)

- **Necrotizing ulcerative stomatitis** is a **severe bacterial infection** affecting the oral mucosa, often seen in:
  - **Severely immunocompromised individuals** (HIV/AIDS, leukemia, chemotherapy).
  - **Malnourished or debilitated patients**.
  - Those with **poor oral hygiene and periodontal disease**.
- **Key features:**
  - **Necrotic ulcer with yellow pseudomembrane**.
  - **Foul odor and severe pain**.
  - **Deep tissue destruction** if untreated.

Differential Diagnoses to Consider:

1. **Necrotizing Ulcerative Gingivitis (NUG) (If Localized to Gingiva)**
   - NUG presents with **punched-out interdental papillae**.
   - **Severe halitosis and spontaneous gingival bleeding** are common.
2. **Necrotizing Ulcerative Periodontitis (NUP) (If Bone Involvement)**
   - Similar to NUG but with **attachment loss and alveolar bone necrosis**.
3. **Oral Squamous Cell Carcinoma (If Persistent and Indurated)**
   - If the ulcer is **non-healing, firm, and rapidly enlarging**, **biopsy is required**.
4. **Mucormycosis (If Palatal or Black Necrosis Present)**
   - If the patient is **diabetic or immunocompromised**, consider **fungal invasion**.

Recommended Next Steps:

1. **Urgent Debridement and Antibiotics**:
    - **Chlorhexidine mouthwash (0.12%)** for plaque control.
    - **Systemic antibiotics (Metronidazole 500 mg TID + Amoxicillin 500 mg TID for 7–10 days)**.
    - **Mechanical debridement of necrotic tissue**.
2. **Evaluate Immunocompetence**:
    - **HIV test, CBC, and nutritional assessment (iron, folate, B12, albumin)**.
    - Consider systemic disease (diabetes, malignancy).
3. **Improve Oral Hygiene and Nutritional Support**:
    - Professional **periodontal therapy** to remove plaque and calculus.
    - Nutritional support if **malnutrition is a contributing factor**.
4. **Monitor for Progression or Malignancy**:
    - If the lesion does **not improve within 2 weeks, biopsy is essential**.
    - Monitor for **deep tissue invasion (osteonecrosis, fungal involvement).**

Conclusion

This lesion is **highly suggestive of necrotizing ulcerative stomatitis**, requiring **urgent antimicrobial therapy, debridement, and systemic evaluation**. Would you like **a detailed management protocol or a diagnostic algorithm for oral necrotic ulcers?**

CASE 93

Reference:

https://www.researchgate.net/publication/303974293/figure/fig2/AS:373176073768962@1465983517609/Squamous-cell-carcinoma-of-the-floor-of-the-mouth-in-condition-of-poor-oral-hygiene.png

Chat-GPT4 answer:

Analysis and Probable Diagnosis

Observations:

1. **Irregular ulcerative lesion on the mandibular alveolar ridge/floor of the mouth**:
   - The lesion appears **erythematous, ulcerated, and irregularly shaped**.
   - The surface shows **areas of necrosis and induration**, with **an inflammatory halo**.
2. **Severe Dental Wear and Poor Oral Hygiene**:
   - The adjacent teeth show **significant attrition, calculus, and discoloration**.
   - This suggests **chronic irritation or a possible traumatic component**.
3. **High-Risk Location for Malignant Lesions**:
   - The **floor of the mouth and alveolar ridge are high-risk sites for oral cancer**.
   - Given the **ulcerative and indurated nature**, malignancy should be strongly considered.

Most Probable Diagnosis: Squamous Cell Carcinoma (SCC) of the Floor of the Mouth

- **Oral SCC is the most common malignancy in this region**.
- **Key Risk Factors**:
  - **Tobacco and alcohol use**.
  - **HPV infection (p16 positivity suggests better prognosis)**.
  - **Chronic irritation (ill-fitting dentures, sharp teeth, betel quid use)**.
  - **Poor oral hygiene and systemic immunosuppression**.
- **Clinical Features Suggesting SCC**:
  - **Non-healing ulcer with indurated borders**.
  - **Firm to palpation and may be associated with regional lymphadenopathy**.
  - **Potential for deep tissue invasion**.

Differential Diagnoses to Consider:

1. **Traumatic Ulcer (If Reversible)**
   - If the lesion is **solely due to mechanical irritation**, removing the cause should lead to **resolution within 2-3 weeks**.
2. **Chronic Osteomyelitis (If Deep Bone Involvement)**
   - If **bone pain and radiographic sequestration are present**, consider **chronic infection**.
3. **Necrotizing Ulcerative Stomatitis (If Associated with Systemic Immunosuppression)**
   - Seen in **HIV/AIDS, diabetes, or malnutrition**.
4. **Tuberculous Ulcer (If History of TB Exposure)**

- **Chronic, painless ulcers with granulomatous histology**.

Urgent Recommended Next Steps:

1. **Incisional Biopsy for Histopathological Confirmation**
   - **Essential to rule out SCC and assess dysplasia/malignancy**.
2. **Imaging (MRI or CT Scan)**
   - Evaluate **tumor invasion depth, bone involvement, and lymph node status**.
3. **Palpation of Cervical Lymph Nodes**
   - Check **levels I-III for nodal metastasis**.
   - **Fine-needle aspiration (FNA) for suspicious lymph nodes**.
4. **Multidisciplinary Oncology Consultation**
   - **If SCC is confirmed, treatment options include**:
     - **Early-stage (T1-T2, N0): Surgery ± Radiotherapy**.
     - **Locally advanced (T3-T4, N+): Surgery + Adjuvant Chemoradiotherapy**.
     - **Metastatic disease: Palliative therapy (Platinum-based chemo, PD-1 inhibitors, radiation)**.
5. **HPV Testing and Risk Factor Management**
   - If HPV-positive, prognosis is generally **better**.
   - **Smoking cessation and alcohol reduction** are crucial.

Conclusion

This lesion is **highly suspicious for squamous cell carcinoma (SCC) of the floor of the mouth, requiring urgent biopsy, imaging, and staging**. Would you like **TNM staging guidelines or treatment algorithms for oral SCC?**

CASE 94

Reference:

https://img.medscapestatic.com/pi/meds/ckb/52/28152tn.jpg

Chat-GPT4 answer:

Analysis and Probable Diagnosis

Observations:

1. **Large, ulcerated, and exophytic lesion on the lateral tongue**:
   - The lesion exhibits **irregular surface thickening, ulceration, and areas of necrosis**.
   - The surrounding tissue appears **erythematous and inflamed**, suggesting **chronic irritation or neoplastic invasion**.
   - Some areas have **a white keratotic component**, indicative of **possible dysplasia or hyperkeratosis**.
2. **Firm, Indurated Mass with Infiltrative Borders**:
   - The lesion appears **nodular and raised**, with **an uneven, ulcerated surface**.
   - **Possible deep tissue infiltration**, making malignancy a strong consideration.
3. **High-Risk Location for Malignancy**:
   - The **lateral border of the tongue** is one of the **most common sites for oral squamous cell carcinoma (SCC)**.
   - Given the **ulcerated, indurated nature and chronicity, SCC is the leading differential**.

Most Probable Diagnosis: Squamous Cell Carcinoma (SCC) of the Lateral Tongue

- **Oral SCC is the most frequent malignant tumor of the tongue**.
- **Risk Factors**:
  - **Tobacco and alcohol use (synergistic effect)**.
  - **HPV-associated SCC (p16 positivity suggests better prognosis)**.
  - **Chronic trauma (sharp teeth, ill-fitting dentures)**.
  - **Poor oral hygiene, immunosuppression, and betel quid use**.
- **Clinical Features Suggesting SCC**:
  - **Non-healing ulcer with induration**.
  - **Firm and possibly fixed to underlying structures**.
  - **Potential regional lymphadenopathy**.

Differential Diagnoses to Consider:

1. **Traumatic Ulcer (If Resolving in 2-3 Weeks)**
   - If due to mechanical trauma, healing should occur once the irritant is removed.
2. **Oral Leukoplakia with Dysplasia (If Persistent White Patches)**
   - A **precancerous condition**, especially if **non-homogeneous and persistent**.
3. **Syphilitic Ulcer (If Painless and Chronic)**
   - **Serologic testing (VDRL, FTA-ABS) is needed** to rule out **tertiary syphilis**.
4. **Tuberculous Ulcer (If Associated with Systemic Symptoms)**

- Consider **oral TB in endemic regions or in immunocompromised patients**.

Urgent Recommended Next Steps:

1. **Incisional Biopsy for Histopathological Confirmation**
   - **Essential to confirm SCC and assess dysplasia or invasion**.
2. **MRI/CT for Tumor Staging**
   - To evaluate **tumor depth, local spread, and nodal involvement**.
3. **Cervical Lymph Node Palpation and Imaging**
   - **Ultrasound-guided fine-needle aspiration (FNA) for suspicious nodes**.
4. **Oncology Referral for Multidisciplinary Management**
   - **Early-stage (T1-T2, N0): Surgery ± Radiotherapy**.
   - **Locally advanced (T3-T4, N+): Surgery + Chemoradiotherapy**.
   - **Metastatic disease: Palliative therapy (Immunotherapy, chemotherapy, radiation)**.
5. **HPV Testing and Lifestyle Modification**
   - **HPV-associated SCC has a better prognosis**.
   - **Smoking and alcohol cessation counseling**.

Conclusion

This lesion is **highly suspicious for squamous cell carcinoma (SCC) of the lateral tongue**. **Urgent biopsy, imaging, and staging are required**. Would you like **a TNM staging guide or a detailed treatment protocol?**

CASE 95

Reference:

https://silverhilldental.com/wp-content/uploads/2021/07/Leukoplakia-4.jpg

Chat-GPT4 answer:

Analysis and Probable Diagnosis

Observations:

1. **White, well-demarcated patch on the dorsal tongue**:
   - The lesion appears **non-removable, irregularly shaped, and slightly raised**.
   - The surrounding mucosa looks **healthy with no significant inflammation**.
2. **No Ulceration, Erythema, or Induration**:
   - The lesion is **not erosive or necrotic**.
   - No **deep infiltration, mass effect, or exophytic growth**.
3. **Common Location for Leukoplakia or Frictional Hyperkeratosis**:
   - The **dorsal tongue and lateral borders** are prone to **chronic irritation, trauma, and epithelial hyperplasia**.

Most Probable Diagnosis: Leukoplakia (Possibly Benign or Dysplastic)

- **Leukoplakia is a white patch or plaque that cannot be wiped off and is not attributable to another specific disease.**
- **Key characteristics:**
  - **Non-painful, persistent, and non-removable**.
  - **Can be benign, dysplastic, or early squamous cell carcinoma**.
  - **Risk factors**: tobacco use, alcohol consumption, chronic irritation.

Differential Diagnoses to Consider:

1. **Frictional Hyperkeratosis (If History of Chronic Trauma)**
   - If the patient has a **history of tongue biting, rough restorations, or excessive brushing**, **hyperkeratosis is likely**.
   - Usually **reversible if the irritant is removed**.
2. **Oral Hairy Leukoplakia (If Associated with HIV or Immunosuppression)**
   - **Caused by Epstein-Barr virus (EBV), typically bilateral, and corrugated**.
   - Common in **immunocompromised patients (e.g., HIV/AIDS, transplant recipients).**
3. **Candidiasis (If Wipes Off with Gauze)**
   - **Pseudomembranous candidiasis (oral thrush) presents as a removable white coating**.
   - Usually **associated with immunosuppression or antibiotic use**.
4. **Lichen Planus (If Reticular or Erosive Pattern)**
   - **Wickham's striae (lacy white lesions) and chronic course suggest lichen planus**.
   - Biopsy is needed for **definitive diagnosis**.
5. **Oral Squamous Cell Carcinoma (If Indurated or Growing)**

- If the lesion is **persistent, growing, or nodular, biopsy is mandatory**.
- SCC often appears as a **non-healing white or red-white lesion**.

Recommended Next Steps:

1. **Remove Potential Irritants (If Present)**
   - If **associated with trauma (e.g., sharp teeth, smoking, or chronic irritation), remove the irritant and reassess in 2–3 weeks**.
2. **Biopsy If the Lesion Persists or Grows**
   - Any **leukoplakia persisting beyond 2–4 weeks should be biopsied** to assess for **dysplasia or carcinoma**.
3. **Monitor for Changes in Size, Texture, or Symptoms**
   - **Regular follow-up is essential**.
   - If the lesion **becomes ulcerated, indurated, or enlarges, immediate biopsy is needed**.
4. **Educate on Risk Factor Modification**
   - Encourage **smoking cessation, alcohol reduction, and proper oral hygiene**.

Conclusion

This lesion is **most consistent with leukoplakia, which may be benign or dysplastic**. **Biopsy is recommended if the lesion persists beyond 2–4 weeks, enlarges, or exhibits surface changes**. Would you like **a leukoplakia biopsy protocol or management guide?**

CASE 96

Reference:

https://cdn.medizzy.com/fIKI89ICbG0HH8aaSAaWxclk_nI=/img/posts/8979bef0-483d-40e9-b9be-f0483d60e925

Chat-GPT4 answer:

Analysis and Probable Diagnosis

Observations:

1. **Extensive maxillary bone exposure with necrosis and sequestration**:
    - There is **significant necrotic bone exposure bilaterally in the maxilla**.
    - The affected regions appear **devoid of soft tissue coverage**, with **a yellowish necrotic appearance**.
2. **Presence of Metallic Reconstruction Plate with Bone Screws**:
    - The patient has undergone **maxillofacial reconstruction with a fixation plate**.
    - The metallic plate appears **well-positioned but surrounded by necrotic tissue**, suggesting **possible osteonecrosis**.
3. **Surrounding Soft Tissue Inflammation and Secondary Infection**:
    - **Erythematous mucosa**, with **visible areas of dehiscence and potential purulent exudate**.

Most Probable Diagnosis: Medication-Related Osteonecrosis of the Jaw (MRONJ) or Osteoradionecrosis (ORN)

- Given the **exposed, necrotic maxillary bone with hardware involvement**, the most likely diagnosis is **severe MRONJ or osteoradionecrosis**.
- **Key Risk Factors for MRONJ:**
    - **History of bisphosphonate or denosumab therapy** for osteoporosis or malignancy (multiple myeloma, bone metastases).
    - **Previous dental extractions, implant placement, or trauma** in a patient on antiresorptive therapy.
- **Key Risk Factors for ORN:**
    - **History of radiotherapy (>50 Gy to the maxillofacial region)**.
    - **Progressive bone necrosis without history of bisphosphonates**.
    - **Delayed healing and chronic non-healing wound post-surgery or trauma**.

Differential Diagnoses to Consider:

1. **Post-Surgical Necrosis (If Recent Maxillary Surgery)**
    - Could be **a non-healing surgical wound in a compromised host**.
    - However, the **extent of necrosis and exposed bone favors MRONJ/ORN**.
2. **Chronic Suppurative Osteomyelitis (If Infectious Component Present)**
    - Typically associated with **diffuse bone destruction, sinus tract formation, and pus drainage**.

    - Requires **bone biopsy and culture** to differentiate from MRONJ/ORN.
3. **Mucormycosis (If Immunosuppression Present)**
    - If the patient is **diabetic, neutropenic, or post-COVID-19**, invasive fungal infection should be ruled out.
    - **Biopsy with PAS/GMS staining is necessary for confirmation**.

Urgent Recommended Next Steps:

1. **Comprehensive Imaging (CBCT or MRI)**
    - Assess **extent of bone destruction, sequestration, and sinus involvement**.
    - **Rule out oroantral communication or secondary maxillary sinusitis**.
2. **Review Medication and Radiation History**
    - **Bisphosphonates, denosumab, chemotherapy?**
    - **History of radiation therapy to the head and neck?**
3. **Microbiological and Histopathological Evaluation**
    - **Biopsy and culture (aerobic, anaerobic, fungal) to rule out secondary infections or malignancy**.
4. **Management Approach**
    - **Conservative Therapy (If Early Stage)**:
        - **Antibiotic therapy (Amoxicillin-Clavulanate or Clindamycin)**.
        - **Chlorhexidine 0.12% mouth rinses**.
        - **Pain management (NSAIDs, opioids if severe)**.
    - **Surgical Therapy (If Advanced, Non-Responsive to Conservative Measures)**:
        - **Sequestrectomy and removal of necrotic bone**.
        - **Hardware removal (if loose or infected)**.
        - **Reconstructive options (free flap if major defect remains)**.

Conclusion

This case is **highly suggestive of advanced MRONJ or osteoradionecrosis**. **Urgent imaging, medication history review, and potential surgical intervention are necessary**. Would you like **detailed staging criteria for MRONJ or ORN surgical management protocols?**

CASE 97

Reference:

https://www.bioral.it/main3/images/docs/Correnti/tatuaggio.jpg

Chat-GPT4 answer:

Analysis and Probable Diagnosis

Observations:

1. **Blue-gray macular lesion on the buccal mucosa and gingiva**:
   - The lesion appears **well-demarcated, flat, and pigmented**.
   - It is **asymptomatic with no signs of ulceration, inflammation, or induration**.
   - No evidence of **exophytic growth, mass effect, or surface changes**.
2. **Presence of Dental Restorations in Adjacent Teeth**:
   - The lesion is in close proximity to **amalgam dental fillings**.
   - There are no signs of **mucosal damage, vascular involvement, or melanocytic proliferation**.
3. **Common Location for Amalgam Tattoo or Foreign Body Implantation**:
   - The **gingiva, buccal mucosa, and alveolar ridge** are common sites for **pigment deposition due to dental materials**.

Most Probable Diagnosis: Amalgam Tattoo

- **Amalgam tattoos occur due to the implantation of dental amalgam particles into the mucosa, leading to localized pigmentation**.
- **Key characteristics:**
   - **Blue-gray to black pigmentation, well-defined but non-palpable**.
   - **Asymptomatic and persistent over time**.
   - **No blanching on compression (rules out vascular lesions)**.
   - **Commonly seen near teeth with amalgam restorations or after dental procedures**.

Differential Diagnoses to Consider:

1. **Melanotic Macule (If No History of Amalgam Placement)**
   - If the patient has **no history of amalgam restorations**, a **melanotic macule should be considered**.
   - **Brown or black pigmentation, often on the lips or gingiva**.
2. **Oral Melanoacanthoma (If Enlarging)**
   - A rare **benign reactive proliferation of melanocytes**.
   - Typically **expands rapidly but resolves spontaneously**.
3. **Melanocytic Nevus (If Well-Demarcated and Congenital)**
   - A benign **pigmented lesion due to melanocyte proliferation**.
   - Typically appears **brown or black rather than blue-gray**.
4. **Oral Malignant Melanoma (If Irregular and Expanding)**

- If the lesion **changes in size, has irregular borders, or exhibits ulceration**, melanoma should be ruled out.
- A **biopsy is mandatory for suspicious pigmented lesions**.

Recommended Next Steps:

1. **Confirm Diagnosis with Radiographic Evaluation (Periapical X-ray or CBCT)**
   - Amalgam tattoos **often contain radiopaque particles**, confirming their etiology.
   - If **radiopacity is absent, biopsy may be needed**.
2. **Monitor for Changes in Size, Shape, or Symptoms**
   - **Amalgam tattoos remain stable over time**.
   - If the lesion **expands, changes color, or ulcerates, biopsy is warranted**.
3. **Biopsy if Atypical Features Are Present**
   - If the lesion has **irregular borders, heterogenous pigmentation, or rapid growth**, biopsy is required to exclude **melanoma or melanocytic neoplasms**.

Conclusion

This lesion is **most consistent with an amalgam tattoo, a benign pigmented lesion caused by dental amalgam deposition in oral mucosa**. **Radiographic evaluation can confirm the diagnosis, and no treatment is needed unless atypical features are present**. Would you like **a biopsy protocol or pigmentary disorder management guide?**

CASE 98

Reference:

https://assets.nhs.uk/nhsuk-cms/images/C0142463-Leukoplakia_on_the_inner_cheek-SPL..max-600x600.jpg

Chat-GPT4 answer:

Analysis and Probable Diagnosis

Observations:

1. **Well-defined white plaque on the lateral border of the tongue**:
   - The lesion appears **thick, adherent, and non-homogeneous**.
   - It does not seem to be easily wipeable.
   - The lateral tongue is a **high-risk site for oral potentially malignant disorders (PMDs)**.
2. **No Signs of Ulceration, Induration, or Erythema**:
   - The lesion does not exhibit **obvious nodularity, ulceration, or surrounding inflammation**.
   - However, non-homogeneous white lesions **warrant careful evaluation for dysplasia**.
3. **No Obvious Vesicles or Fungal Pseudomembrane**:
   - This reduces the likelihood of **oral candidiasis or herpetic lesions**.

Most Probable Diagnosis: Leukoplakia (Potentially Malignant Disorder)

- **Leukoplakia** is a **clinically defined white plaque that cannot be scraped off and has no obvious cause**.
- It is considered a **potentially malignant disorder (PMD)**, particularly if:
  - **Located on the lateral tongue, floor of the mouth, or ventral tongue**.
  - **Non-homogeneous (mixed white and red areas, nodular, verrucous)**.

Differential Diagnoses to Consider:

1. **Oral Lichen Planus (If Bilateral and Reticulated)**
   - Lichen planus often appears with **Wickham's striae (white lace-like patterns)**.
   - Typically **bilateral and chronic**, often involving the buccal mucosa.
2. **Hyperkeratosis from Chronic Trauma (If Frictional or Reactive)**
   - Chronic irritation from **sharp teeth, dentures, or smoking** can cause **localized frictional keratosis**.
   - Unlike leukoplakia, **this should regress after removing the irritant**.
3. **Oral Hairy Leukoplakia (If Immunosuppressed Patient)**
   - **Vertical corrugated white plaques on the lateral tongue**.
   - Associated with **Epstein-Barr Virus (EBV) and HIV**.
4. **Oral Candidiasis (If Wipeable)**
   - **Pseudomembranous candidiasis ("thrush") presents as a white patch that can be scraped off**.
   - More common in **immunocompromised individuals**.

Recommended Next Steps:

1. **Assess for Risk Factors**
    - **Smoking, alcohol consumption, betel nut chewing, or HPV infection?**
    - **Chronic trauma from sharp restorations or dentures?**
2. **Biopsy is Required for Confirmation**
    - Any **persistent leukoplakic lesion, especially on high-risk sites like the lateral tongue, should be biopsied**.
    - Biopsy will determine whether **dysplasia, carcinoma in situ, or squamous cell carcinoma (SCC) is present**.
3. **Monitor for Changes**
    - **Non-homogeneous leukoplakia has a higher risk of malignant transformation**.
    - If biopsy shows **mild dysplasia, close follow-up is needed**.
    - If **moderate to severe dysplasia is present, excision is recommended**.

Conclusion

This lesion is **most concerning for leukoplakia, a potentially malignant disorder**. **A biopsy is strongly recommended** to determine if **dysplasia or carcinoma is present**. Would you like **a flowchart for managing leukoplakia or histopathologic criteria for biopsy interpretation?**

CASE 99

Reference:

https://exodontia.info/wp-content/uploads/2021/07/Erythroplakia_of_Palate-1017x686-1.jpg

Chat-GPT4 answer:

Analysis and Probable Diagnosis

Observations:

1. **Well-demarcated, erythematous lesion on the buccal mucosa**:
   - The affected area appears **red, inflamed, and atrophic**.
   - The lesion has a **smooth, shiny surface with some areas of hyperemia**.
   - The mucosa looks **thinned with no evidence of exophytic growth**.
2. **Presence of a Dental Restoration Adjacent to the Lesion**:
   - A **metallic restoration** is visible on the upper molar.
   - This suggests **a possible localized reaction to the restoration material or chronic irritation**.
3. **No Induration, Ulceration, or Exophytic Growth**:
   - The lesion appears **localized, non-nodular, and non-ulcerative**.
   - No **deep infiltration or malignant features**.

Most Probable Diagnosis: Contact Mucositis or Allergic Stomatitis

- This lesion is likely **a contact reaction to dental materials (e.g., amalgam, gold, nickel, composite resins) or a reaction to chemical irritants (e.g., toothpaste, mouthwash, food additives)**.
- **Key characteristics:**
  - **Erythematous, atrophic, or hyperemic mucosa adjacent to a dental restoration**.
  - **Burning or irritation sensation in response to certain foods or products**.
  - **No ulceration or deep tissue involvement**.

Differential Diagnoses to Consider:

1. **Chronic Irritative Trauma (If Due to Mechanical Friction)**
   - If **adjacent to sharp dental restorations, ill-fitting prostheses, or excessive brushing**.
   - Typically **reversible upon removal of the irritant**.
2. **Erythematous Candidiasis (If Associated with Burning or Antibiotic Use)**
   - **Candidiasis often presents as diffuse erythema with burning sensation**.
   - Common in **immunocompromised individuals, denture wearers, or those on corticosteroids/antibiotics**.
3. **Lichen Planus (If Reticular or Chronic)**
   - **Erythematous or erosive oral lichen planus can mimic mucositis**.
   - Often associated with **Wickham's striae (white lacy pattern)**.
4. **Plasma Cell Gingivostomatitis (If Associated with Diffuse Hyperemia)**

- A rare **idiopathic inflammatory condition** presenting as **erythematous mucosa with a burning sensation**.
- Diagnosed with **biopsy showing plasma cell infiltrate**.

5. **Oral Squamous Cell Carcinoma (If Persistent and Indurated)**
   - If the lesion **persists for more than 3 weeks, enlarges, or becomes indurated, a biopsy is necessary**.
   - SCC often presents as **erythematous, non-healing, or ulcerated mucosa**.

Recommended Next Steps:

1. **Remove Possible Irritants or Allergens**:
   - **Assess for potential triggers (e.g., dental materials, oral hygiene products, spicy foods).**
   - If **associated with a dental restoration, replacing it with a non-metallic alternative may help**.
2. **Topical Corticosteroids for Symptomatic Relief**:
   - **Low-dose corticosteroids (e.g., clobetasol gel, dexamethasone rinse) can help reduce inflammation.**
   - If **burning or discomfort is present, consider antihistamines or anesthetic gels**.
3. **Fungal Testing (If Suspicious for Candidiasis)**:
   - **Culture or KOH preparation** can confirm the presence of **Candida albicans**.
   - If positive, **antifungal treatment (e.g., nystatin, fluconazole) is needed**.
4. **Biopsy if the Lesion Persists Beyond 3 Weeks**:
   - If the lesion **fails to resolve after removal of the irritant or treatment, a biopsy is warranted to exclude dysplasia or malignancy**.

Conclusion

This lesion is **most consistent with contact mucositis or allergic stomatitis, likely due to an adjacent dental restoration or chemical irritant**. **If the lesion resolves after removing the irritant, no further treatment is needed. However, persistent lesions beyond 3 weeks require a biopsy**. Would you like **a mucositis management guide or biopsy protocol?**

CASE 100

Reference:

https://exodontia.info/wp-content/uploads/2021/07/Oral-cancer9-877x729-1.jpg

Chat-GPT4 answer:

Analysis and Probable Diagnosis

Observations:

1. **White Plaque-Like Lesion on the Lateral Border of the Tongue**:
    - The lesion appears **irregular, non-homogeneous, and keratotic**.
    - It has **well-demarcated white areas with some underlying erythema**.
    - There are **both smooth and rough (verrucous) textures** within the lesion.
2. **Non-Removable and Asymmetric Distribution**:
    - The lesion does **not appear to be wipeable**, which **rules out pseudomembranous candidiasis**.
    - The **asymmetric, localized presentation** suggests **a chronic pathology**.
3. **Localized Erythema and Induration**:
    - Some regions appear **erythematous and slightly thickened**, which could indicate **dysplasia**.
    - The lesion **does not have clear ulceration but appears chronic**.
4. **Common Location for Leukoplakia or Dysplasia**:
    - The **lateral tongue is a high-risk site for leukoplakia, epithelial dysplasia, and early oral squamous cell carcinoma (OSCC)**.

Most Probable Diagnosis: Non-Homogeneous Leukoplakia with Possible Dysplasia

- **Leukoplakia is a potentially malignant disorder (PMD) characterized by white plaques that cannot be scraped off and have no identifiable cause**.
- **Key characteristics:**
    - **Non-homogeneous leukoplakia (irregular, verrucous, or nodular features) has a higher risk of malignant transformation**.
    - **Occurs in high-risk sites such as the lateral tongue, floor of the mouth, and soft palate**.
    - **Associated with tobacco use, alcohol consumption, HPV, and chronic irritation**.

Differential Diagnoses to Consider:

1. **Oral Lichen Planus (If Bilateral and Reticular)**
    - If the lesion has **bilateral distribution with Wickham's striae, lichen planus is a consideration**.
    - However, **this lesion appears unilateral, favoring leukoplakia**.
2. **Epithelial Dysplasia or Early OSCC (If Indurated or Ulcerated)**
    - **If the lesion is firm, thickened, or has ulcerations, biopsy is mandatory to rule out carcinoma**.

- **Squamous cell carcinoma often arises from pre-existing leukoplakia, especially in high-risk locations**.

3. **Hyperplastic Candidiasis (If Antifungal-Responsive)**
   - If the lesion **responds to antifungal treatment (fluconazole/nystatin), candidiasis is the primary cause**.
   - **This lesion does not appear to have a fungal overgrowth pattern**.

Recommended Next Steps:

1. **Urgent Biopsy to Rule Out Malignancy**
   - **Non-homogeneous leukoplakia has a 5-25% risk of malignant transformation**.
   - **Histopathology will determine the presence of dysplasia or early carcinoma**.
2. **Evaluate Risk Factors**
   - **Smoking, alcohol consumption, HPV status, and chronic irritation should be assessed**.
3. **Monitor Closely If Benign, But Consider Excision If Dysplasia Is Present**
   - **If mild dysplasia is found, conservative monitoring is possible**.
   - **If moderate/severe dysplasia is present, excisional biopsy is recommended**.

Conclusion

This lesion is **most consistent with non-homogeneous leukoplakia, which carries a high risk of malignant transformation**. **A biopsy is mandatory to rule out dysplasia or early squamous cell carcinoma**. Would you like **a detailed biopsy protocol or a management guide for PMDs?**
