# Supplementary material for: Diagnostic Performance of ChatGPT-4o in Analyzing Oral Mucosal Lesions: A Comparative Study with Experts
Source: Medicina (Kaunas). 2025 Jul 30;61(8):1379. doi: 10.3390/medicina61081379 (PMC12388129; doi:10.3390/medicina61081379)
Supplement: Supplementary file 1 [file medicina-61-01379-s001.zip › Supplementary document S4.pdf]

Supplementary document S4: ChatGPT-4o responses on oral mucosal lesions – Case-by-Case analysis			
**Case number**	**ChatGPT4o diagnosis**	**Expert panel diagnosis**	**Comparative evaluation**
1	Oral lichen planus	Oral leukoplakia	Plausible
2	Oral leukoplakia	Oral leukoplakia	Correct
3	Pyogenic granuloma	Pyogenic granuloma	Correct
4	Torus palatinus	Torus palatinus	Correct
5	Oral squamous cell carcinoma	Oral squamous cell carcinoma	Correct
6	Vascular malformation	Vascular malformation	Correct
7	Ranula	Ranula	Correct
8	Gingivitis	Gingivitis	Correct
9	Oral lichen planus	Oral lichen planus	Correct
10	Mucocele	Mucocele	Correct
11	Torus palatinus	Minor salivary gland adenoma	Not plausible
**12**	Oral lichen planus, erosive or atrophic form	Oral lichen planus erosive form	Correct
13	Herpes virus infection	Hereditary telangiectasia	Plausible
14	Medication related osteonecrosis	Medication related osteonecrosis	Correct
15	Oral squamous cell carcinoma	Melanoma	Plausible
16	Oral squamous cell carcinoma	Oral squamous cell carcinoma	Correct
17	Oral submucous fibrosis	Mandibular tori	Not plausible
18	Oral squamous cell carcinoma	Oral squamous cell carcinoma	Correct
19	Aftous stomatitis	Aftous stomatitis	Correct
20	Oral squamous cell carcinoma	Melanoma	Plausible
21	Oral squamous cell carcinoma	Oral squamous cell carcinoma	Correct
22	Oral squamous cell carcinoma	Oral squamous cell carcinoma	Correct
**23**	**Erosive oral lichen planus**	Erythroplakia	Plausible
**24**	**Gingival hyperplasia**	**Gingival hyperplasia**	Correct
**25**	**Benign migratory glossitis**	**Benign migratory glossitis**	Correct
**26**	**Oral candidiasis**	**Oral candidiasis**	Correct
**27**	**Gingival hyperplasia**	**Exostosis**	Plausible
28	Pyogenic granuloma	Mandibular torus	Not plausible
29	Black hairy tongue	Black hairy tongue	Correct
30	Oral squamous cell carcinoma	Oral squamous cell carcinoma	Correct
31	Oral squamous cell carcinoma	Oral squamous cell carcinoma	Correct
32	Oral squamous cell carcinoma	Oral squamous cell carcinoma	Correct
33	Oral linea alba	Oral linea alba	Correct
34	Oral leukoplakia	Oral leukoplakia	Correct
35	Oral leukoplakia	Oral leukoplakia	Correct
36	Oral squamous cell carcinoma	Oral squamous cell carcinoma	Correct
37	Oral squamous cell carcinoma	Oral squamous cell carcinoma	Correct
38	Oral lichen planus	Dental decubitus	Plausible
39	Aphtha	Aphtha	Correct
40	Oral squamous cell carcinoma	Oral squamous cell carcinoma	Correct
41	Oral lichen planus	Oral lichen planus	Correct

42	Oral lichen planus	Oral lichen planus	Correct
43	Oral squamous cell carcinoma	Oral squamous cell carcinoma	Correct
44	Medication related osteonecrosis	Medication related osteonecrosis	Correct
45	Medication related osteonecrosis	Oral squamous cell carcinoma	Not plausible
46	Oral squamous cell carcinoma	Oral squamous cell carcinoma	Correct
47	Pyogenic Granuloma	Pyogenic Granuloma	Correct
48	Pyogenic Granuloma	Pyogenic Granuloma	Correct
49	Oral squamous cell carcinoma	Oral squamous cell carcinoma	Correct
50	Oral squamous cell carcinoma	Oral squamous cell carcinoma	Correct
51	Oral squamous cell carcinoma	Oral squamous cell carcinoma	Correct
52	Oral squamous cell carcinoma	Oral squamous cell carcinoma	Correct
53	Medication related osteonecrosis	Oral squamous cell carcinoma	Not plausible
54	Oral squamous cell carcinoma	Oral squamous cell carcinoma	Correct
55	Oral squamous cell carcinoma	Oral squamous cell carcinoma	Correct
56	Oral squamous cell carcinoma	Oral squamous cell carcinoma	Correct
57	Oral squamous cell carcinoma	Oral squamous cell carcinoma	Correct
58	Oral squamous cell carcinoma	Oral squamous cell carcinoma	Correct
59	Oral squamous cell carcinoma	Oral squamous cell carcinoma	Correct
60	Oral squamous cell carcinoma	Oral squamous cell carcinoma	Correct
61	Oral squamous cell carcinoma	Oral squamous cell carcinoma	Correct
62	Erythroleukoplakia	Oral squamous cell carcinoma	Plausible
63	Oral squamous cell carcinoma	Oral squamous cell carcinoma	Correct
64	Oral squamous cell carcinoma	Oral squamous cell carcinoma	Correct
65	Aphtha	Mucocele	Plausible
66	Mucocele	Mucocele	Correct
67	Mucocele	Mucocele	Correct
68	Torus palatinus	Torus palatinus	Correct
69	Torus palatinus	Torus palatinus	Correct
70	Oral lichen planus	Oral lichen planus	Correct
71	Oral leukoplakia	Oral leukoplakia	Correct
72	Black hairy tongue	Black hairy tongue	Correct
73	Oral candidiasis	Oral candidiasis	Correct
74	Oral lichen planus	Oral lichen planus	Correct
75	Chronic Gingivitis	Chronic Gingivitis	Correct
76	Fibroma	Fibroma	Correct
77	Fibroma	Fibroma	Correct
78	Fibroma	Fibroma	Correct
79	Oral squamous cell carcinoma	Oral squamous cell carcinoma	Correct
80	Pyogenic granuloma	Pyogenic granuloma	Correct
81	Angioma	Angioma	Correct
82	Oral papilloma	Oral papilloma	Correct
83	Oral squamous cell carcinoma	Oral squamous cell carcinoma	Correct
84	Oral squamous cell carcinoma	Oral squamous cell carcinoma	Correct

85	Aphtha	Aphtha	Correct
86	Aphtha	Aphtha	Correct
87	Mucocele	Mucocele	Correct
88	Oral squamous cell carcinoma	Oral squamous cell carcinoma	Correct
89	Mucocele	Mucocele	Correct
90	Gingival hyperplasia	Gingival hyperplasia	Correct
91	Ranula	Ranula	Correct
92	Frictional keratosis	Frictional keratosis	Correct
93	Oral squamous cell carcinoma	Oral squamous cell carcinoma	Correct
94	Oral squamous cell carcinoma	Oral squamous cell carcinoma	Correct
95	Oral leukoplakia	Oral leukoplakia	Correct
96	Medication related osteonecrosis	Medication related osteonecrosis	Correct
97	Amalgam tattoo	Amalgam tattoo	Correct
98	Oral leukoplakia	Oral leukoplakia	Correct
99	Contact Mucositis	Erythroplakia	Plausible
100	Oral leukoplakia	Oral leukoplakia	Correct
